# Supplementary material for: Constrained G4 structures unveil topology specificity of known and new G4 binding proteins
Source: Sci Rep. 2021 Jun 29;11:13469. doi: 10.1038/s41598-021-92806-8 (PMC8241873; doi:10.1038/s41598-021-92806-8)
Supplement: Supplementary file 1 — Supplementary Information. [file 41598_2021_92806_MOESM1_ESM.pdf]

Supplementary Figures

Supplementary Table1

MS-based proteomic analyses of proteins interacting with constrained structures **1a**, **2** and the control **8** (duplex).

Supplementary Table2

List of 425 proteins enriched on constrained G4 structures **1a-2** compared to duplex control **8** (fold change $\geq$ 2 and p-value < 0.05, allowing to reach a false discovery rate (FDR) inferior to 5%).

Supplementary Table3

List of 214 out of 425 proteins enriched on G4 structures that have been described as nucleic acid interacting factors, as indicated by terms from GO Molecular functions data analyses.

Supplementary Table4.1

List of 31 proteins found enriched on system 1a relatively to system 2

Supplementary Table4.2

List of 204 proteins found enriched on system 2 relatively to system 1a

Supplementary Table4.3

List of 190 proteins Common to system 1a and 2

Supplementary Figure S1

Schematic representation of the constrained molecules used in this study

Supplementary Figure S2

CD spectra of G-quadruplex-forming conjugate 1-7 at 1 µM (5) or 2 µM (1a, 1b, 2, 3, 4, 6, 7) in 10 mM Tris-HCl, pH 7.4, 20°C in the presence of 100 mM KCl

Supplementary Figure S3

Western-blotting analysis and quantification of molecular interactions of known-G4 interacting factors with constrained G4 structures adopting different topologies (see Supplementary Figure S1). The relative quantity was obtained from the ratio between the signal observed from a

particular construction and the sum of signals from all wells. Digital data were processed and quantified using ImageLab (Biorad) or ImageJ softwares. Green, purple and oranges boxes indicate interaction with Parallel **1a**, antiparallel **2** or duplex DNA **8** respectively.

Supplementary Figure S4

Enriched KEGG pathways for the 214 nuclear proteins interacting with nucleic acids found enriched on constrained G4 structures **1a** and **2**.

Supplementary Figure S5

Most significant pathways and processes covered by constrained G4 interacting proteins. Enriched KEGG pathways of (**A**) the 190 proteins present in the Common group and (**B**) the 203 proteins enriched on construction **2** (*i.e.* antiparallel topology with two lateral loops). KEGG analysis of Common group proteins (**1a** and **2**) and proteins enriched on construction **2** showed similar significant enriched pathways compared to those covered by total constrained G4 interacting proteins (see Figure 2A).

Supplementary Figure S6

A. Western-blot detection of NELFE and NELFA proteins immunoprecipitated from a HeLa cell line overexpressing an ectopic Flag-tagged form of the NELF-E protein (Flag-eluted material). **B**. Uncropped and unprocessed versions of Western-blot signals. Chemiluminescence signals were acquired using the ChemyDoc Touching Imaging System (Bio-Rad). When additional signals corresponding to previous hybridizations with other antibodies are present, they are indicated in Italics. Not used lanes for hybridization are indicated by a red square.

Supplementary Figure S7A-E

Uncropped and unprocessed versions of Western-blot signals from Figure 5. Chemiluminescence signals were acquired using the ChemyDoc Touching Imaging System (Bio-Rad). Exposition times are indicated. When additional signals corresponding to previous hybridizations with other antibodies are present, they are indicated in Italics.

Supplementary Figure S8

Uncropped and unprocessed versions of Western-blot signals from Figure 6C. Chemiluminescence signals were acquired using the ChemyDoc Touching Imaging System (Bio-Rad).

Supplementary Figure S9A and S9B

Method used to quantify immunofluorescence from DAPI (**S9A**, blue) and γH2AX signals (**S9B**, red) from Figure 6C. Raw images were processed as described in Supplementary Figure S9A. Raw and Modified Images used for quantification and for the final figure are shown.

Supplementary Figure S10A-10B

Uncropped and unprocessed versions of Western-blot signals from Supplementary Figure S3A. Chemiluminescence signals were acquired using the ChemyDoc Touching Imaging System (Bio-Rad).

Supplementary Table 1

Protein names	Gene names	Razor + unique peptides	system 2 vs system B Significant	system 2 vs system B p-value	system 2 vs system B log2(Fold Change)	system 1a vs system B Significant	system 1a vs system B p-value	system 1a vs system B log2(Fold Change)	system 2 vs system 1a Significant	system 2 vs system 1a p-value	system 2 vs system 1a log2(Fold Change)	Intensity B-1	Intensity B-2	Intensity B_3	Intensity 2-1	Intensity 2-2	Intensity 2-3	Intensity 1a_1	Intensity 1a_2	Intensity 1a_3	Majority protein IDs	Mol. weight kDa	Unique + razor sequence coverage	id
Thymidylate kinase	DTYMK	19	+	3,49E-11	8,38	+	1,13E-12	12,18	+	4,59E-08	-3,80	17,12	17,34	17,34	25,39	25,86	25,68	29,35	29,60	29,38	P23919;H7BZ20;H7C312	75,9	23,819	737
Echinoderm microtubule-associated protein-like 3	EML3	13	+	7,84E-11	8,30		1,00E+00	0,09	+	8,70E-11	8,21	17,12	17,34	17,34	25,26	25,68	25,76	17,34	17,63	17,11	B7WPE2;Q32P44;G3V195;9	18,1	95,283	206
Tight junction protein ZO-1	TJP1	10	+	7,81E-06	6,53	+	2,19E-02	2,02	+	1,50E-04	4,51	17,12	17,34	17,34	23,78	23,87	23,72	20,03	20,63	17,18	A0A087X0K9;G5E9E7;G3V1	8,4	187,85	43
Sorting nexin-27	SNX27	5	+	4,30E-10	6,39		1,00E+00	0,09	+	4,93E-10	6,30	17,12	17,34	17,34	23,70	23,55	23,72	17,34	17,63	17,11	Q96L92;A0A2R8Y8A7	11,6	61,264	1217
Dedicator of cytokinesis protein 5	DOCK5	8	+	2,13E-09	5,52		1,00E+00	0,44	+	4,54E-09	5,08	16,99	16,67	17,08	22,37	22,47	22,46	17,34	17,63	17,11	Q9H7D0	5,5	215,31	1281
UDP-N-acetylglucosamine--peptide N-acetylglucosaminyltransferase 110 kDa subunit	OGT	7	+	2,25E-09	5,51		1,00E+00	0,09	+	2,63E-09	5,42	17,12	17,34	17,34	22,66	22,67	22,99	17,34	17,63	17,11	O15294	9,4	116,92	563
Zinc finger protein 598	ZNF598	6	+	1,46E-05	5,35		6,35E-02	1,36	+	1,50E-04	3,99	17,32	17,45	17,36	22,42	22,79	22,99	20,53	18,22	17,49	H3BQQ2;Q86UK7	9,3	93,287	474
Nuclear receptor coactivator 5	NCOA5	6	+	4,32E-08	5,14	+	1,21E-08	5,92		3,45E-02	-0,79	17,12	17,34	17,34	21,88	22,64	22,68	22,90	23,68	22,99	Q9HCD5	17,4	65,536	1289
pre-mRNA 3' end processing protein WDR33	WDR33	4	+	9,49E-08	4,96	+	3,25E-05	2,50	+	3,80E-05	2,46	17,12	17,34	17,34	22,05	22,15	22,47	19,16	20,43	19,71	Q9C0J8	4,5	145,89	1265
Calcium/calmodulin-dependent protein kinase type II subunit delta;Calcium/calmodulin-dependent protein kinase type II subunit alpha	CAMK2D;CAMK2A;CAMK2B	2	+	4,02E-09	4,83	+	4,68E-09	4,75		7,36E-01	0,08	17,12	17,34	17,34	22,18	22,06	22,04	22,05	22,20	21,79	D6R938;E9PBG7;E9PF82;Q14		56,298	279
Rapamycin-insensitive companion of mTOR	RICTOR	6	+	2,87E-07	4,74		1,00E+00	2,42	+	1,12E-04	2,32	17,12	17,34	17,34	22,01	22,32	21,66	19,13	20,46	19,45	Q6R327	5	192,22	1080
Periplakin	PPL	7	+	1,93E-08	4,69		1,00E+00	1,53	+	6,46E-07	3,15	17,51	17,22	17,64	22,45	22,10	21,89	18,72	19,06	19,19	K7EK18;O60437;K7EQ71	5,3	204,49	523
WD repeat-containing protein 82	WDR82	5	+	7,33E-09	4,63		1,00E+00	2,77	+	2,05E-05	1,86	17,12	17,34	17,34	22,06	21,99	21,63	20,16	19,93	20,02	Q6UXN9	24	35,079	1083
Myotubularin-related protein 5	SBF1	9	+	8,96E-09	4,57		1,00E+00	0,09	+	1,08E-08	4,47	17,12	17,34	17,34	21,78	21,77	21,93	17,34	17,63	17,11	O95248	6,8	208,44	639
cAMP-dependent protein kinase type II-alpha regulatory subunit	PRKAR2A	5	+	7,87E-08	4,55		1,00E+00	3,78		2,96E-02	0,77	16,91	16,59	17,00	21,71	21,57	20,88	20,40	20,95	20,50	P13861;H7C1L0	21	45,518	705
Nck-associated protein 1	NCKAP1	6	+	4,38E-08	4,55		1,00E+00	1,98	+	6,40E-06	2,57	17,12	17,34	17,34	22,30	21,59	21,57	19,52	19,17	19,05	Q9Y2A7	6,6	128,79	1379
Mediator of DNA damage checkpoint protein 1	MDC1	5	+	1,22E-07	4,53		1,00E+00	0,09	+	1,48E-07	4,43	17,12	17,34	17,34	21,16	22,14	22,08	17,34	17,63	17,11	Q14676	3,4	226,66	988
Actin-like protein 6A	ACTL6A	5	+	1,28E-08	4,52		1,00E+00	1,40	+	3,50E-07	3,12	17,98	18,14	18,58	22,69	22,81	22,76	19,58	19,57	19,76	O96019	13,1	47,46	652
Kinesin-like protein KIF2A	KIF2A	6	+	7,63E-06	4,51	+	8,96E-03	1,66	+	2,89E-04	2,85	19,23	18,37	18,24	22,54	23,28	23,54	19,26	21,15	20,42	O00139	9,3	79,954	541
Telomere-associated protein RIF1	RIF1	8	+	3,13E-07	4,45		1,00E+00	1,62	+	1,48E-05	2,83	17,34	17,81	17,50	21,60	21,67	22,75	19,24	18,93	19,35	Q5UIP0;H7BZN3	5	274,46	1059
Serine/threonine-protein kinase WNK1	WNK1	5	+	1,54E-08	4,39		1,00E+00	0,09	+	1,87E-08	4,30	17,12	17,34	17,34	21,55	21,83	21,58	17,34	17,63	17,11	F5GWT4;Q9H4A3	3,6	225,5	363
CLIP-associating protein 1	CLASP1	6	+	3,92E-07	4,39		1,00E+00	2,89	+	1,82E-03	1,50	17,12	17,34	17,34	21,40	21,31	22,25	20,07	20,69	19,70	Q7Z460;F8WA11;H0Y5T1	6,4	169,45	1099
Kinesin-like protein KIF2C;Kinesin-like protein	KIF2C	8	+	3,86E-06	4,36	+	1,22E-03	2,07	+	5,96E-04	2,30	19,60	21,48	20,26	24,81	24,75	24,87	22,54	22,94	22,07	Q99661;Q5JR89;Q5JR91	15,6	81,312	1232
Citron Rho-interacting kinase	CIT	11	+	5,62E-08	4,35		1,00E+00	0,31	+	1,09E-07	4,04	18,82	19,39	19,48	23,87	23,37	23,52	19,72	19,34	19,56	O14578;H7BXI3	7,7	231,43	549
Calcium/calmodulin-dependent protein kinase type II subunit gamma	CAMK2G	9	+	2,73E-07	4,34	+	6,27E-07	3,95		2,64E-01	0,39	21,53	21,22	20,35	25,37	25,19	25,56	24,74	25,17	25,04	Q5SWX3;Q8WU40;A0A2Q3	26,7	57,796	1050
Transformation/transcription domain-associated protein	TRRAP	6	+	3,60E-08	4,32		1,00E+00	0,09	+	4,38E-08	4,23	17,12	17,34	17,34	21,94	21,39	21,44	17,34	17,63	17,11	F2Z2U4;Q9Y4A5;H0Y4W2;A	1,8	436,13	359
Ubiquitin carboxyl-terminal hydrolase 7;Ubiquitin carboxyl-terminal hydrolase	USP7	8	+	7,37E-04	4,30	+	6,87E-04	4,35		9,60E-01	-0,04	15,94	19,93	17,13	21,83	22,16	21,91	21,13	22,79	22,11	Q93009;H3BND8	10,3	128,3	1184
tRNA (cytosine(34)-C(5))-methyltransferase	NSUN2	14	+	9,39E-06	4,17		6,11E-02	1,02	+	8,96E-05	3,15	20,07	20,25	19,88	24,14	24,09	24,49	20,31	22,34	20,61	Q08J23	26,6	86,47	928
Ral GTPase-activating protein subunit beta	RALGAPB	5	+	2,52E-08	4,17		1,00E+00	0,09	+	3,10E-08	4,07	17,12	17,34	17,34	21,35	21,32	21,62	17,34	17,63	17,11	Q86X10;A2A2F0	6,5	166,8	1117
Protein SEC13 homolog	SEC13	6	+	1,09E-05	4,14		1,37E-01	0,79	+	6,03E-05	3,36	19,92	18,25	18,64	23,15	23,23	22,85	19,23	20,55	19,39	P55735;A8MXL6	33,9	35,54	844
Pre-mRNA-splicing factor ATP-dependent RNA helicase PRP16	DHX38	12	+	1,78E-05	4,11	+	3,67E-03	1,97	+	2,15E-03	2,14	19,32	19,10	19,28	23,33	23,16	23,53	20,38	20,63	22,58	Q92620	13,2	140,5	1173
TBC1 domain family member 4	TBC1D4	4	+	3,07E-08	4,11		1,00E+00	2,14	+	1,80E-05	1,97	17,12	17,34	17,34	21,55	21,30	21,27	19,50	19,62	19,10	A0A3B3IRT3;O60343	4,4	119,93	147
PERQ amino acid-rich with GYF domain-containing protein 2	GIGYF2	7	+	4,76E-04	4,11	+	1,32E-02	2,39		5,41E-02	1,72	17,14	19,09	17,48	22,07	21,39	22,58	21,51	20,85	18,52	I1E4Y6;Q6Y7W6;C9IHW1;E	8,6	152,53	493
Protein virilizer homolog	KIAA1429	14	+	2,71E-05	4,06		3,80E-01	-0,49	+	1,07E-05	4,55	19,35	18,87	18,13	22,72	22,41	23,41	17,94	19,42	17,51	Q69YN4	12,4	202,02	1070
Serine hydroxymethyltransferase, mitochondrial;Serine hydroxymethyltransferase	SHMT2	7	+	1,08E-05	4,06		1,00E+00	1,92	+	1,36E-03	2,14	19,58	19,57	19,17	22,54	23,59	24,35	21,05	22,06	20,96	P34897;G3V2Y4;G3V5L0;H	16,7	55,992	764
Cytoplasmic FMR1-interacting protein 1	CYFIP1	10	+	3,52E-06	4,05		1,00E+00	2,14	+	1,16E-03	1,91	18,13	19,82	18,38	22,95	22,60	22,93	21,05	20,84	20,85	Q7L576	9,4	145,18	1091
Rho guanine nucleotide exchange factor 7	ARHGEF7	4	+	5,19E-07	4,00		1,00E+00	0,09	+	6,39E-07	3,91	17,12	17,34	17,34	20,62	21,38	21,80	17,34	17,63	17,11	B1ALK7;A0A2R8YG42;Q14	8,4	79,831	142
Ribonuclease H2 subunit C	RNASEH2C	5	+	1,45E-03	3,97		5,32E-02	1,97		4,97E-02	2,00	20,34	17,76	19,58	23,45	22,90	23,24	22,40	22,08	19,10	H0YEF2;E9PN81;Q8TDP1;H	42,4	17,12	349
Uncharacterized protein KIAA1522	KIAA1522	2	+	7,37E-08	3,96		1,00E+00	0,09	+	9,14E-08	3,87	17,12	17,34	17,34	21,01	21,10	21,56	17,34	17,63	17,11	Q9P206	4,3	107,09	1330
Poly(ADP-ribose) glycohydrolase	PARG	5	+	6,68E-07	3,95	+	6,88E-07	3,93		9,68E-01	0,01	17,52	18,49	18,28	22,09	21,82	22,22	21,72	22,48	21,89	Q86W56	6,2	111,11	1115
Zinc finger CCCH domain-containing protein 13	ZC3H13	3	+	2,67E-07	3,88		1,00E+00	0,09	+	3,31E-07	3,79	17,12	17,34	17,34	20,68	21,14	21,62	17,34	17,63	17,11	Q5T200	2,8	196,63	1051
E3 SUMO-protein ligase RanBP2	RANBP2	3	+	6,82E-04	3,88		1,00E+00	1,38	+	1,01E-02	2,50	17,12	17,34	17,34	19,95	23,37	20,10	19,00	18,57	18,35	P49792	1,2	358,2	818
Engulfment and cell motility protein 2	ELMO2	3	+	1,49E-06	3,83		1,00E+00	0,09	+	1,85E-06	3,74	17,12	17,34	17,34	20,29	21,46	21,54	17,34	17,63	17,11	Q5JVZ5;Q96JJ3;Q5JW01;H	8,4	82,364	1045
RNA-binding protein 33	RBM33	2	+	1,88E-07	3,83		1,00E+00	0,09	+	2,34E-07	3,73	17,12	17,34	17,34	21,32	21,32	20,63	17,34	17,63	17,11	Q96EV2;H7BXM6;A8MTF7;F	3,1	129,98	1200
Ribose-phosphate pyrophosphokinase 1;Ribose-phosphate pyrophosphokinase 2	PRPS1;PRPS2	5	+	2,19E-03	3,82	+	1,14E-02	2,86		3,20E-01	0,95	18,46	20,70	16,09	22,47	22,07	22,17	21,05	21,57	21,21	B1ALA9;P60891;A0A2R8Y7	28,4	31,392	188
Fibroblast growth factor;Fibroblast growth factor 2	FGF2	2	+	5,68E-08	3,81		1,00E+00	2,95		5,80E-03	0,86	17,12	17,34	17,34	20,97	21,19	21,06	20,40	19,88	20,34	A0A0A0MQV6;A0A087WUF	7,3	30,752	16
N-acylneuraminate cytidylyltransferase	CMAS	10	+	4,16E-07	3,81		9,53E-03	0,99	+	5,52E-06	2,82	22,08	22,06	21,09	25,53	25,59	25,53	22,76	22,86	22,58	Q8NFW8	30,2	48,379	1149
CLIP-associating protein 2	CLASP2	12	+	2,87E-03	3,78		8,41E-01	0,19	+	3,95E-03	3,59	19,04	20,77	19,92	23,47	23,63	23,97	21,20	21,58	17,53	E7ERI8;E7EW49	10	165,63	315
SWI/SNF-related matrix-associated actin-dependent regulator of chromatin subfamily B member 1	SMARCB1	2	+	1,77E-06	3,76		1,00E+00	2,11	+	1,07E-03	1,66	17,12	17,34	17,34	20,49	21,53	21,06	18,86	19,93	19,31	G5E975;Q12824;A0A0U1RC	8,1	45,05	427
Exocyst complex component 1	EXOC1	2	+	9,63E-08	3,75		1,00E+00	3,68		8,00E-01	0,07	17,12	17,34	17,34	20,79	21,42	20,83	21,02	20,87	20,95	Q9NV70	2,9	101,98	1309
Myotubularin-related protein 1	MTMR1	4	+	4,89E-07	3,73		1,00E+00	0,75	+	3,36E-06	2,98	17,12	17,34	17,34	20,66	21,42	20,89	17,59	18,41	18,03	Q8NEC6;F8WA39;Q13613;F	12,9	39,822	406
Protein LSM12 homolog	LSM12	4	+	2,08E-05	3,73	+	2,75E-04	2,67		5,12E-02	1,05	21,14	21,50	20,52	24,94	24,90	24,50	22,62	24,37	24,19	K7ELG9;Q3MHD2	29,6	24,955	526
DNA topoisomerase 2-binding protein 1	TOPBP1	2	+	6,82E-07	3,72		1,00E+00	0,09	+	8,52E-07	3,63	17,12	17,34	17,34	21,37	20,36	21,22	17,34	17,63	17,11	A0A2R8YD63;Q92547	1,5	170,13	138
SWI/SNF complex subunit SMARCC2	SMARCC2	6	+	5,56E-05	3,71		1,00E+00	0,60	+	2,17E-04	3,11	19,03	18,98	18,87	22,54	22,73	22,75	20,33	20,28	18,09	F8VXC8;Q8TAQ2	5,5	136,18	394
Deoxynucleotidyltransferase terminal-interacting protein 1	DNTTIP1	2	+	7,22E-08	3,68		1,00E+00	0,09	+	9,10E-08	3,59	17,12	17,34	17,34	20,84	21,11	20,89	17,34	17,63	17,11	H7C1M5;Q9H147;F2Z2A4;H	10,4	31,664	488
Aurora kinase A	AURKA	5	+	7,17E-08	3,68	+	4,30E-09	5,03	+	2,93E-04	-1,35	18,50	18,09	18,55	22,03	22,03	22,12	23,21	23,43	23,59	O14965;A3KFJ0;Q5QPD4	19,6	45,809	556
Microtubule-actin cross-linking factor 1, isoforms 1/2/3/5	MACF1	6	+	6,26E-06	3,68	+	1,77E-06	4,27		1,74E-01	-0,59	17,31	17,00	17,42	20,17	20,85	21,74	21,94	21,42	21,17	H3BPE1;H3BQK9;Q9UPN3	1,1	856,87	470
RNA-binding protein 47	RBM47	4	+	4,50E-07	3,65		1,00E+00	1,58	+	5,23E-05	2,07	17,12	17,34	17,34	20,41	20,88	21,47	18,86	18,84	18,83	A0AV96;B7Z827;D6RBP6;D	13	64,098	160
Cleavage and polyadenylation specificity factor subunit 2	CPSF2	12	+	1,14E-02	3,65		4,69E-01	0,87	+	3,95E-02	2,78	20,05	20,57	17,74	22,73	23,18	23,40	21,80	21,83	17,36	Q9P2I0	23,4	88,486	1333
Protein transport protein Sec16A	SEC16A	25	+	5,14E-03	3,64		4,20E-01	-0,84	+	1,42E-03	4,49	21,66	22,44	22,52	25,85	25,79	25,92	21,93	23,56	18,60	O15027;F1T0I1	19,3	251,89	560
Coatomer subunit alpha;Xenin;Proxenin	COPA	10	+	2,36E-05	3,63		5,60E-01	0,28	+	4,53E-05	3,35	19,27	20,53	18,88	23,35	22,81	23,41	20,26	20,10	19,17	A0A3B3IT15;A0A3B3IS84;P	10,1	136,02	150
Gamma-tubulin complex component 3	TUBGCP3	6	+	1,08E-05	3,62		1,00E+00	-0,65	+	2,67E-06	4,28	17,88	18,20	19,37	21,61	22,63	22,09	17,54	17,98	17,98	Q96CW5;A0A3B3ITE3;A0A0	8,9	103,57	1196

[illegible]

Actin-binding LIM protein 1	ABLIM1	4	+	6,04E-04	-2,68	+	2,77E-02	-1,37	+	3,43E-02	-1,31	22,63	22,80	21,04
Rho guanine nucleotide exchange factor 1	ARHGEF1	16	+	2,54E-08	-7,42	+	1,39E-08	-7,93		1,00E+00	0,51	25,09	25,50	25,28
Poly(rC)-binding protein 2 (Fragment)	PCBP2	2	+	6,15E-10	-6,11	+	3,55E-10	-6,49		1,00E+00	0,38	23,89	23,90	23,74
TATA-box-binding protein;TATA box-binding protein-like protein 2	TBP;TBPL2	4	+	4,52E-08	-5,91	+	2,06E-07	-4,98		3,17E-02	-0,93	22,78	23,41	22,18
DNA mismatch repair protein Msh3	MSH3	32	+	7,72E-06	-5,65	+	6,49E-05	-4,35		6,86E-02	-1,30	27,20	27,90	27,35
RNA-binding protein 3	RBM3	2	+	2,78E-09	-5,35	+	2,81E-05	-1,86		1,00E+00	-3,50	23,24	22,89	23,13
Inosine-5-monophosphate dehydrogenase 2	IMPDH2	11	+	1,47E-05	-4,91	+	2,53E-05	-4,60		6,09E-01	-0,31	26,06	26,47	26,23
A-kinase anchor protein 12	AKAP12	5	+	6,38E-05	-4,90	+	2,95E-05	-5,39		1,00E+00	0,50	22,67	22,77	22,82
Poly(rC)-binding protein 2	PCBP2	16	+	6,37E-09	-4,85	+	5,63E-09	-4,91		7,87E-01	0,07	29,76	30,12	29,71
Nucleolysin TIA-1 isoform p40	TIA1	2	+	3,86E-08	-4,83	+	1,10E-06	-3,31		1,00E+00	-1,53	22,74	22,49	22,47
Recombining binding protein suppressor of hairless	RBPJ	3	+	1,62E-08	-4,81	+	8,16E-09	-5,19		1,00E+00	0,38	22,20	22,88	22,55
Nucleolysin TIAR	TIAL1	12	+	1,15E-08	-4,75	+	4,00E-08	-4,13		3,69E-02	-0,62	27,44	27,61	27,22
Thymocyte nuclear protein 1	THYN1	5	+	1,69E-08	-4,47	+	1,80E-08	-4,44		9,07E-01	-0,03	25,83	26,36	25,85
Methylated-DNA--protein-cysteine methyltransferase	MGMT	2	+	7,38E-07	-4,43	+	3,62E-06	-3,68		7,77E-02	-0,75	22,09	21,87	22,54
X-ray repair cross-complementing protein 5	XRCC5	50	+	1,85E-07	-4,40	+	1,30E-07	-4,58		5,85E-01	0,18	31,53	32,17	31,77
X-ray repair cross-complementing protein 6	XRCC6	44	+	5,59E-07	-4,32	+	3,95E-07	-4,50		6,35E-01	0,17	32,25	32,84	32,31
Flap endonuclease 1	FEN1	19	+	2,62E-08	-4,16	+	6,27E-08	-3,78		1,45E-01	-0,39	30,60	30,97	30,38
TATA-binding protein-associated factor 172	BTAF1	18	+	1,05E-07	-4,11	+	5,87E-08	-4,39		3,47E-01	0,28	24,48	24,97	24,81
Poly(rC)-binding protein 1	PCBP1	12	+	2,38E-07	-4,08	+	7,24E-08	-4,66		8,58E-02	0,59	29,54	29,54	29,33
DNA-directed RNA polymerase III subunit RPC1	POLR3A	12	+	3,53E-05	-3,63	+	4,32E-05	-3,54		8,58E-01	-0,09	23,70	24,29	23,01
UV excision repair protein RAD23 homolog B	RAD23B	2	+	3,87E-07	-3,48	+	1,55E-07	-3,86		1,00E+00	0,38	20,80	21,56	21,28
DNA polymerase subunit gamma-1	POLG	2	+	1,63E-06	-3,44	+	6,60E-07	-3,81		1,00E+00	0,38	21,40	21,60	20,51
Inosine-5-monophosphate dehydrogenase;Inosine-5-monophosphate dehydrogenase 1	IMPDH1	2	+	4,64E-07	-3,34	+	1,80E-07	-3,71		1,00E+00	0,38	20,74	21,44	21,03
DNA-(apurinic or apyrimidinic site) lyase 2	APEX2	3	+	3,90E-07	-3,24	+	8,96E-07	-2,94		1,00E+00	-0,29	21,89	22,54	22,41
COUP transcription factor 2;COUP transcription factor 1	NR2F1;NR2F2	2	+	2,65E-07	-3,20	+	1,22E-08	-4,52		1,00E+00	1,32	21,69	21,85	22,10
Bifunctional polynucleotide phosphatase/kinase;Polynucleotide 3-phosphatase;Polynucleotide 5-hydroxyl-kinase	PNKP	11	+	3,05E-06	-3,08	+	3,54E-05	-2,29		3,04E-02	-0,79	25,89	26,29	25,25
Krueppel-like factor 16	KLF16	2	+	4,61E-05	-3,00	+	3,87E-07	-5,29		1,00E+00	2,29	22,20	23,07	22,67
Replication protein A 32 kDa subunit	RPA2	8	+	5,51E-07	-2,98	+	1,41E-06	-2,68		2,41E-01	-0,31	27,50	27,77	27,53
DNA damage-binding protein 1	DDB1	51	+	7,49E-06	-2,92	+	8,93E-05	-2,15		4,14E-02	-0,77	30,22	30,96	30,47
Replication protein A 70 kDa DNA-binding subunit;Replication protein A 70 kDa DNA-binding subunit, N-terminally processed	RPA1	28	+	2,13E-06	-2,88	+	3,11E-06	-2,75		6,62E-01	-0,13	30,13	30,67	30,22
DNA-directed RNA polymerase III subunit RPC2	POLR3B	8	+	9,81E-07	-2,82	+	8,19E-08	-3,74		4,61E-03	0,92	23,89	23,97	23,58
DNA-directed RNA polymerase III subunit RPC4	POLR3D	4	+	4,09E-04	-2,76	+	1,15E-04	-3,27		3,43E-01	0,51	23,23	23,21	22,52
Replication protein A 14 kDa subunit	RPA3	4	+	6,20E-05	-2,64	+	8,12E-05	-2,55		8,20E-01	-0,09	22,61	23,34	23,85
Poly [ADP-ribose] polymerase 1	PARP1	58	+	7,06E-06	-2,58	+	3,18E-06	-2,84		3,93E-01	0,26	32,22	32,94	32,40
SWI/SNF-related matrix-associated actin-dependent regulator of chromatin subfamily E member 1-related	HMG20B	3	+	2,79E-05	-2,43	+	1,67E-04	-1,93		1,52E-01	-0,50	22,64	23,19	22,58
DNA ligase 3	LIG3	28	+	9,29E-05	-2,41	+	4,48E-05	-2,65		5,38E-01	0,23	27,67	28,37	27,89
DNA repair protein XRCC1	XRCC1	12	+	3,31E-05	-2,29	+	2,23E-05	-2,41		7,18E-01	0,11	26,25	26,88	26,44

[illegible]

Eukaryotic translation initiation factor 2 subunit 3;Putative eukaryotic translation initiation factor 2 subunit 3-like protein	EIF2S3;EIF2S3L	13	8,56E-03	0,98	9,37E-02	0,55	1,78E-01	0,43	26,01	25,64	25,37	26,70	26,70	26,54	26,29	25,76	26,62	P41091;Q2VIR3	43,4	51,109	790
Heterogeneous nuclear ribonucleoprotein U-like protein 1	HNRNPUL1	8	3,22E-02	0,71	3,51E-01	0,28	1,58E-01	0,43	23,51	23,36	23,94	24,10	24,37	24,48	23,90	24,23	23,51	A0A0A0MRA5;B7Z4B8;Q9BL	18,1	85,939	55
Cyclin-T1	CCNT1	7	2,62E-02	0,83	2,39E-01	0,39	1,98E-01	0,43	22,37	23,17	23,41	23,79	23,62	24,03	23,62	23,31	23,20	O60563	15,3	80,684	597
RNA-binding protein 8A	RBM8A	3	1,90E-01	0,74	5,71E-01	0,31	4,29E-01	0,43	22,37	21,48	21,94	23,37	23,09	21,57	23,04	21,86	21,82	Q9Y5S9	32,2	19,889	1400
RNA-binding protein NOB1	NOB1	3	2,15E-01	0,97	4,84E-01	0,53	5,62E-01	0,44	19,05	20,67	19,23	19,09	20,76	22,02	19,81	19,78	20,95	Q9ULX3;H3BUR4	11,4	46,674	1363
60S ribosomal protein L14	RPL14	2	2,37E-01	0,55	8,26E-01	0,10	3,25E-01	0,46	23,61	23,26	22,72	23,95	23,79	23,53	24,30	22,64	22,95	E7EPB3;P50914	17,7	14,558	309
F-actin-capping protein subunit alpha-1	CAPZA1	12	2,50E-02	0,95	2,02E-01	0,49	2,25E-01	0,46	24,50	24,38	23,37	25,12	25,28	24,69	24,78	24,71	24,22	P52907	66,4	32,922	835
Pre-mRNA cleavage complex 2 protein Pcf11	PCF11	7	3,32E-02	0,94	2,34E-01	0,48	2,50E-01	0,46	21,91	22,89	21,73	23,09	22,92	23,34	22,18	23,17	22,62	O94913;E9PKN0;E9PQ01	5,9	173,05	634
Myosin regulatory light chain 12A;Myosin regulatory light polypeptide 9	MYL12A;MYL9	10	3,20E-01	0,70	7,31E-01	0,24	5,04E-01	0,46	25,54	23,34	23,49	25,15	24,80	24,52	25,60	23,56	23,92	J3QRS3;P19105;J3KTJ1;P24	77,4	20,457	514
Thyroid hormone receptor-associated protein 3	THRAP3	16	1,06E-01	0,66	6,13E-01	0,19	2,36E-01	0,47	25,39	26,02	25,08	25,93	26,34	26,21	25,67	26,32	25,09	A0A3B3IT29;Q9Y2W1	19,8	103,52	156
Eukaryotic initiation factor 4A-I	EIF4A1	10	9,71E-01	0,02	3,04E-01	-0,46	2,89E-01	0,47	23,77	25,13	23,78	24,52	24,36	23,85	23,85	24,16	23,30	P60842;J3KT12;J3QR64;J3	31	46,153	850
La-related protein 7	LARP7	9	4,49E-01	0,52	9,50E-01	0,04	4,86E-01	0,48	23,05	23,26	21,60	22,93	23,51	23,02	23,63	23,24	21,16	Q4G0J3;H0YA82	20,6	66,898	1032
Calmodulin-2	CALM2;CALM3;CALM1	2	5,21E-02	1,23	2,04E-01	0,76	4,10E-01	0,48	21,75	19,22	20,34	21,72	21,58	21,71	21,62	20,71	21,24	F8WBR5;M0QZ52;G3V479;F	46,2	7,372	409
60S ribosomal protein L6	RPL6	6	1,83E-01	0,37	6,62E-01	-0,11	9,04E-02	0,48	23,89	23,86	23,60	23,93	24,34	24,18	23,88	23,78	23,34	Q02878	27,4	32,728	912
U3 small nucleolar RNA-associated protein 14 homolog A;U3 small nucleolar RNA-associated protein 14 homolog C	UTP14A;UTP14C	2	1,95E-01	0,33	5,30E-01	-0,15	6,98E-02	0,48	18,19	18,22	18,46	18,79	18,68	18,40	18,02	18,07	18,32	Q9BVJ6;X6RJY0;Q5TAP6	3,1	87,977	1251
60S ribosomal protein L30	RPL30	4	6,89E-01	0,13	3,01E-01	-0,35	1,65E-01	0,49	23,64	23,07	23,35	23,65	23,28	23,53	23,61	22,51	22,88	E5RI99;P62888	35,1	12,656	301
Translational activator GCN1	GCN1L1	13	1,85E-01	-0,48	1,74E-02	-0,97	1,78E-01	0,49	22,74	22,78	23,19	22,94	21,70	22,63	21,97	21,94	21,90	Q92616	7,2	292,75	1172
Pre-mRNA-splicing factor ATP-dependent RNA helicase DHX15	DHX15	27	1,48E-02	0,87	2,25E-01	0,38	1,25E-01	0,49	26,88	26,89	26,71	27,24	27,97	27,87	27,22	27,54	26,85	O43143	42,8	90,932	570
RNA-binding protein 26	RBM26	8	2,39E-01	0,53	9,39E-01	0,03	2,67E-01	0,50	23,02	24,17	23,03	23,70	23,92	24,20	22,71	24,02	23,59	A0A087X0H9;Q5T8P6	9,8	113,79	42
LIM domain and actin-binding protein 1	LIMA1	3	4,59E-01	-0,51	1,55E-01	-1,03	4,57E-01	0,51	22,28	22,50	21,74	21,92	21,71	21,35	22,20	21,92	19,33	Q9UHB6;F8VRN8;F8VS07;F8	5,3	85,225	1343
60 kDa heat shock protein, mitochondrial	HSPD1	5	7,61E-01	0,09	1,78E-01	-0,43	1,09E-01	0,52	21,06	21,65	21,56	21,65	21,56	21,34	21,11	21,34	20,53	P10809;E7EXB4;E7ESH4	15,7	61,054	691
Cytoplasmic dynein 1 intermediate chain 2	DYNC1I2	5	4,95E-01	0,42	8,73E-01	-0,10	4,03E-01	0,52	22,26	22,60	21,65	21,81	23,23	22,74	22,40	23,05	20,76	Q13409;E7EQL5;E9PGG1;E7	13,3	71,456	955
Histone H2A type 1-J;Histone H2A type 1-H;Histone H2A.J;Histone H2A type 2-C;Histone H2A type 2-A;Histone H2A type 1-D;Histone H2A type 1;Histone H2A;Histone H2A type 1-C;Histone H2A type 3;Histone H2A type 1-B/E	HIST1H2AJ;HIST1H2AH;H2AFJ;HIST2H	3	8,79E-01	-0,05	8,20E-02	-0,57	1,05E-01	0,52	23,22	23,21	23,14	23,20	23,11	23,12	23,24	22,31	22,31	A0A0U1RR32;A0A0U1RRH7;	23,1	18,481	106
Mediator of RNA polymerase II transcription subunit 17	MED17	4	1,37E-01	0,78	6,10E-01	0,25	2,99E-01	0,52	20,54	22,23	20,47	21,30	22,13	22,15	21,24	21,59	21,17	E9PM72;A0A1W2PRX4;A0A	11	57,119	119
Ribosome biogenesis protein BRX1 homolog	BRIX1	5	3,87E-01	0,29	4,93E-01	-0,23	1,39E-01	0,53	21,90	22,35	22,13	22,50	22,14	22,63	22,51	21,79	21,39	Q8TDN6	14,7	41,401	1157
Leucine zipper protein 1	LUZP1	7	4,09E-01	0,51	9,78E-01	-0,02	3,95E-01	0,53	21,98	23,13	20,31	22,41	22,25	22,29	22,10	21,51	21,76	Q86V48	9,4	120,27	1111
Death-inducer obliterator 1	DIDO1	10	6,59E-01	-0,14	5,38E-02	-0,66	1,13E-01	0,53	23,52	24,00	24,22	23,89	23,31	24,13	23,41	23,21	23,13	Q9BTC0	8,3	243,87	1245
Uveal autoantigen with coiled-coil domains and ankyrin repeats	UACA	17	3,14E-01	0,62	8,85E-01	0,09	3,83E-01	0,53	24,65	24,61	23,73	25,13	24,87	24,83	25,14	25,20	22,90	H0YNH8;Q9BZF9;F5H2B9	14,6	161,31	465
T-complex protein 1 subunit theta	CCT8	5	3,44E-01	0,41	7,76E-01	-0,12	2,29E-01	0,53	21,34	21,76	21,35	21,99	22,02	21,66	21,41	22,21	20,45	P50990;H7C4C8	11,1	59,62	823
T-complex protein 1 subunit beta	CCT2	5	1,55E-01	0,82	5,94E-01	0,29	3,43E-01	0,53	20,06	22,18	21,72	22,29	22,22	21,92	21,42	22,30	21,11	F8VQ14;F5GWF6;P78371	16,6	44,812	361
Eukaryotic initiation factor 4A-III;Eukaryotic initiation factor 4A-III, N-terminally processed	EIF4A3	13	3,37E-02	0,91	3,25E-01	0,38	1,79E-01	0,53	25,91	25,58	24,64	26,44	26,58	25,85	25,93	25,82	25,52	P38919;I3L3H2	36	46,871	781
Spectrin beta chain, non-erythrocytic 1	SPTBN1	63	1,71E-01	-0,99	4,68E-02	-1,53	4,38E-01	0,54	28,36	28,45	27,87	27,69	27,34	26,67	27,92	27,22	24,94	Q01082;A0A087WUZ3	38,2	274,61	905
Replication factor C subunit 5	RFC5	5	7,89E-01	-0,15	2,30E-01	-0,70	3,39E-01	0,55	22,19	22,23	20,35	21,34	21,56	21,42	21,39	21,28	19,99	P40937;C9JH72;F5H0U6	23,5	38,496	788
RNA-binding protein PNO1	PNO1	4	4,59E-02	0,87	4,24E-01	0,32	1,75E-01	0,56	20,43	20,78	20,31	20,82	21,34	21,99	20,31	21,39	20,78	Q9NRX1	20,2	27,924	1302
60S ribosomal protein L10	RPL10	4	2,45E-01	0,31	3,41E-01	-0,25	5,09E-02	0,56	24,54	24,33	24,26	24,59	24,68	24,79	24,50	23,93	23,95	X1WI28;P27635;F8W7C6;A	26	22,975	749
Unconventional myosin-XVIIIa	MYO18A	24	1,77E-01	0,71	7,79E-01	0,14	2,70E-01	0,57	24,14	24,36	23,84	24,96	24,73	24,77	24,72	25,06	22,99	A0A0D9SFK2;Q92614	18,4	231,1	91
60S ribosomal protein L27a	RPL27A	2	2,48E-01	0,36	4,79E-01	-0,21	7,94E-02	0,57	23,02	23,15	23,00	23,48	23,40	23,35	23,39	22,78	22,36	E9PJD9;E9PLL6;P46776;E9	23,1	10,127	339
Mortality factor 4-like protein 2	MORF4L2	5	9,95E-02	0,85	5,65E-01	0,28	2,47E-01	0,57	22,72	22,74	20,92	22,97	22,93	23,01	22,74	22,19	22,27	A0A0A0MTS9;Q15014	26,6	26,713	66
Myosin light polypeptide 6	MYL6	7	5,58E-01	0,34	6,81E-01	-0,23	3,28E-01	0,57	25,68	24,28	24,00	24,96	25,34	24,66	25,41	24,32	23,51	F8W1R7;G3V1V0;P60660;	51,7	16,29	398
60S ribosomal protein L13	RPL13	5	5,07E-02	0,65	8,41E-01	0,06	7,12E-02	0,59	24,50	25,11	24,57	25,53	25,27	25,31	25,19	24,72	24,44	P26373;J3QSB4	24,2	24,261	746
60S ribosomal protein L13a;Putative 60S ribosomal protein L13a protein RPL13AP3	RPL13A;RPL13a;RPL13AP3	4	2,00E-01	0,51	8,28E-01	-0,08	1,42E-01	0,60	24,34	23,89	23,10	24,49	24,16	24,22	24,25	23,46	23,36	P40429;M0QYS1;Q8J015;Q	20,7	23,577	787
Cirhin	CIRH1A	3	4,22E-02	0,71	7,20E-01	0,11	7,74E-02	0,60	20,03	20,25	20,18	20,87	20,86	20,86	20,29	20,81	19,69	Q969X6;A0A0J9YXV4;A0A0J	6	76,889	1189
60S ribosomal protein L21	RPL21	4	1,71E-01	0,52	8,15E-01	-0,08	1,18E-01	0,60	23,80	23,78	23,12	24,19	23,85	24,21	24,17	23,13	23,15	P46778;G3V1B3;M0R181	28,1	18,565	806
E3 ubiquitin-protein ligase DTX3L	DTX3L	6	8,37E-01	-0,06	4,86E-02	-0,67	6,89E-02	0,61	21,95	21,71	21,64	21,39	21,84	21,89	21,34	21,38	20,58	Q8TDB6	9,7	83,553	1155
S-phase kinase-associated protein 1	SKP1	3	3,88E-01	0,49	8,22E-01	-0,12	2,84E-01	0,61	21,25	21,28	20,08	21,78	21,37	20,93	21,11	21,54	19,59	E5RJR5;P63208;E5RGM3;E7	32,5	18,72	304
CCR4-NOT transcription complex subunit 3	CNOT3	3	3,20E-02	0,92	4,25E-01	0,30	1,25E-01	0,61	21,12	21,15	20,21	21,82	21,65	21,76	20,98	21,75	20,66	B7Z6J7;O75175;H7C148;H	16,4	76,568	210
DnaJ homolog subfamily B member 1	DNAJB1	2	8,52E-01	0,05	6,52E-02	-0,57	4,75E-02	0,63	19,11	18,57	18,72	18,93	18,96	18,66	18,55	18,27	17,86	P25685;M0R080	7,9	38,044	741
Ubiquitin-associated protein 2-like	UBAP2L	13	1,26E-01	0,51	6,94E-01	-0,12	6,56E-02	0,63	25,37	25,76	25,42	25,89	26,00	26,18	25,27	25,96	24,94	F8W726;Q14157	23,2	113,63	402
Eukaryotic translation initiation factor 3 subunit L	EIF3L	2	9,81E-01	-0,02	1,00E+00	-0,65	3,83E-01	0,63	19,78	20,02	19,59	19,26	19,74	20,33	19,18	17,49	20,76	B0QY90;B0QY89;Q9Y262	4,3	55,161	181
Integrator complex subunit 6	INTS6	11	5,39E-02	1,71	1,98E-01	1,08	4,33E-01	0,64	19,54	22,52	21,12	21,89	22,78	23,65	21,37	23,16	21,88	Q9UL03	17,9	100,39	1358
MKI67 FHA domain-interacting nucleolar phosphoprotein	NIFK	2	9,31E-02	0,76	1,00E+00	0,12	1,50E-01	0,64	17,73	18,13	17,92	18,15	19,72	18,19	18,12	18,02	18,01	C9J6C5;Q9BYG3;C9JBD8	16,3	19,553	228
Mediator of RNA polymerase II transcription subunit 4	MED4	3	2,56E-02	0,78	6,48E-01	0,14	5,59E-02	0,64	19,53	18,83	18,79	19,64	19,84	20,00	19,42	19,29	18,85	Q9NPJ6;Q5T911	16,3	29,745	1297
60S ribosomal protein L8	RPL8	5	9,81E-01	0,02	1,00E+00	-0,63	3,52E-01	0,64	22,11	23,16	22,38	23,24	23,54	20,92	22,52	21,86	21,40	E9PKZ0;P62917;G3V1A1;E9	38	22,389	345
Probable 28S rRNA (cytosine(4447)-C(5))-methyltransferase	NOP2	5	8,55E-01	0,09	2,97E-01	-0,55	2,27E-01	0,64	22,52	22,84	22,25	22,73	22,39	22,77	22,67	22,61	20,68	P46087	7,6	89,301	804
Zinc finger protein 638	ZNF638	11	6,31E-02	1,80	2,07E-01	1,16	4,68E-01	0,65	21,92	22,65	20,51	23,37	23,52	23,59	23,41	24,38	20,75	Q14966	7,9	220,62	992
Gamma-adducin	ADD3	9	3,25E-01	-0,32	1,15E-02	-0,98	6,42E-02	0,65	24,67	24,83	24,31	23,94	24,09	24,80	23,28	23,83	23,76	Q9UEY8	19,3	79,154	1341
14-3-3 protein zeta/delta	YWHAZ	4	9,32E-01	-0,03	1,13E-01	-0,69	1,30E-01	0,65	20,65	21,86	21,83	21,52	21,30	21,42	20,51	21,38	20,39	E7EX29;P63104;B0AZS6;B7	22,4	28,036	328
Periodic tryptophan protein 2 homolog	PWP2	2	8,95E-01	-0,04	5,17E-02	-0,70	6,46E-02	0,66	19,44	20,01	19,58	19,13	19,73	20,05	18,76	18,86	19,30	A0A0B4J2E5;Q15269;A0A0	3,2	102,45	75
Rho GTPase-activating protein 1	ARHGAP1	4	7,72E-01	0,18	4,59E-01	-0,48	3,11E-01	0,66	19,96	22,19	20,74	20,30	21,56	21,58	19,69	21,10	20,66	Q07960;H0YE29	13,2	50,435	922
60 kDa SS-A/Ro ribonucleoprotein	TROVE2	3	3,57E-01	-0,28	1,00E+00	-0,96	4,62E-02	0,67	20,23	20,32	20,28	19,46	20,12	20,40	19,37	19,06	19,53	P10155	4,5	60,67	689
Heterogeneous nuclear ribonucleoprotein H2	HNRNPH2	2	1,04E-02	0,85	5,23E-01	0,18	3,09E-02	0,67	19,72	19,81	20,12	20,46	21,17	20,57	20,11	20,05	20,02	P55795	8	49,263	845
Poly(U)-binding-splicing factor PUF60	PUF60	18	3,00E-02	0,63	8,58E-01	-0,05	2,21E-02	0,68	27,32	27,66	27,16	28,01	28,11	27,91	27,37	27,53	27,10	A0A0J9YYL3;A0A0J9YVP6;A	50,7	54,626	100
Catenin delta-1	CTNND1	2	3,64E-01	0,36	4,31E-01	-0,31	1,08E-01	0,68	19,52	20,15	19,50	19,83	20,02	20,41	18,65	19,57	20,01	C9J2R2;O60716;E9PRE2	2,5	104,85	243
Fibrinogen gamma chain	FGG	9	3,26E-02	0,68	9,93E-01	0,00	3,31E-02	0,68	23,43	24,04	23,52	24,44	24,45	24,15	23,47	23,99	23,54	C9JEU5;C9JC84;P02679	29,9	50,322	231
Melanoma-associated antigen D2	MAGED2	7	7,24E-01	-0,13	5,36E-02	-0,82	9,69E-02	0,68	22,27	23,33	22,81	22,70	22,59	22,72	21,96	22,60	21,39	Q5H909;Q9UNF1;Q5H907	15,6	63,138	1038
Telomeric repeat-binding factor 2	TERF2	3	9,71E-01	-0,02	2,15E-01	-0,70	2,27E-01	0,68	19,15	20,84	21,20	19,94	20,84	20,34	20,24	19,35	19,48	U3KQ35;H3BR06;Q15554;	19,3	26,663	476
Probable ATP-dependent RNA helicase DDX17	DDX17	20	2,05E-03	0,99	2,22E-01	0,31	1,63E-02	0,69	26,06	26,46	26,22	27,06	27,29	27,38	26,57	26,68	26,42	A0A1X7SBZ2;A0A1W2PQS	35,9	80,253	118
Unconventional myosin-Ig;Minor histocompatibility antigen HA-2	MYO1G	3	9,54E-01	-0,04	1,00E+00	-0,72	1,77E-01	0,69	19,71	20,01	19,79	20,10	20,73	18,59	19,17	19,19	19,00	A0A3B3IU30;B0I1T2	4,4	110,56	157
Uncharacterized protein C17orf85	C17orf85	4	7,80E-02	0,74	8,96E-01	0,05	9,69E-02	0,69	20,33	21,25	20,22	20,95	21,53	21,56	20,85	21,01	20,10	Q53F19;K7ELP6	7,6	70,592	1035
THO complex subunit 4	ALYREF	8	1,06E-02	0,76	7,78E-01	0,07	1,71E-02	0,69	24,31	24,31	23,82	25,05	24,78	24,90	24,25	24,22	24,17	Q86V81;E9PB61	37,4	26,888	1112
Heat shock cognate 71 kDa protein	HSPA8	21	6,19E-03	0,84	5,72E-01	0,14	1,60E-02	0,70	27,87	27,84	27,66	28,33	28,71	28,84	27,81	27,99	27,99	P11142;E9PKE3;E9PN89;E9	39,2	70,897	694
Histone H3.2;Histone H3;Histone H3.3C;Histone H3.1t;Histone H3.3;Histone H3.1	HIST2H3A;H3F3B;H3F3A;HIST2H3PS2	2	6,98E-02	0,55	5,88E-01	-0,15	2,76E-02	0,71	22,70	22,97	23,19	23,41	23,51	23,60	23,20	22,75	22,46	Q71DI3;K7EMV3;K7EP01;B4	28,7	15,388	1086
40S ribosomal protein S12	RPS12	4	5,99E-01	0,28	4,39E-01	-0,42	2,08E-01	0,71	22,18	22,14	23,66	22,67	22,40	23,76	22,08	21,77	22,87	P25398	40,9	14,515	740
H/ACA ribonucleoprotein complex non-core subunit NAF1	NAF1	3	9,68E-02	1,29	4,26E-01	0,58	3,36E-01	0,71	17,64	17,08	17,59	17,65	19,02	19,51	19,27	16,45	18,34	Q96HR8	8,3	53,716	1206
Barrier-to-autointegration factor;Barrier-to-autointegration factor, N-terminally processed	BANF1	2	7,43E-01	0,22	4,66E-01	-0,49	3,00E-01	0,71	18,98	21,12	21,55	20,94	20,02	21,35	20,62	19,56	19,98	O75531	40,4	10,058	616
Bcl-2-associated transcription factor 1	BCLAF1	4	4,80E-02	1,21	3,74E-01	0,50	2,11E-01	0,71	21,57	22,94	21,20	22,40	23,45	23,49	21,96	23,15	22,09	Q9NYF8;A0A1W2PQ43;E9P	5,8	106,12	1320
Zinc finger CCCH-type antiviral protein 1	ZC3HAV1	3	8,92E-01	-0,05	7,20E-02	-0,77	9,02E-02	0,72	21,06	21,92	21,50	21,36	21,60	21,36	20,48	21,51	20,17	C9J6P4;Q7Z2W4	3,5	114,08	229
60S ribosomal protein L17	RPL17;RPL17-C18orf32	4	4,39E-01	0,32	3,19E-01	-0,41	9,46E-02	0,73	24,30	23,66	23,62	24,10	23,88	24,55	24,24	22,90	23,21	A0A087WXM6;J3QQT2;J3KF	30,2	19,586	32
60S ribosomal protein L31	RPL31	3	5,88E-01	0,17	9,32E-02	-0,56	3,72E-02	0,73	23,00	22,71	22,73	22,90	22,93	23,11	22,83	21,79	22,13	H7C2W9;C9JU56;B7Z4E3;B	29,6	12,701	207
Insulin-like growth factor 2 mRNA-binding protein 3	IGF2BP3	16	1,40E-02	0,83	7,95E-01	0,07	2,17E-02	0,76	25,55	25,96	25,65	26,31	26,60	26,73	25,97	26,03	25,38	O00425	35,4	63,704	545
Histone-binding protein RBBP4	RBBP4;RBBP7	5	1,77E-01	0,54	5,53E-01	-0,23	6,69E-02	0,76	22,80	22,92	21,77	23,16	23,38	22,56	22,00	22,41	22,41	Q09028;H0YCT5;H0YF10;H	15,1	47,655	929
Paraspeckle component 1	PSPC1	4	6,99E-02	1,61	3,09E-01	0,84	3,55E-01	0,76	18,70	21,57	22,30	22,27	22,28	22,82	22,23	21,36	21,50	Q8WXF1;X6RDA4	12,8	58,743	1164
Calcium homeostasis endoplasmic reticulum protein	CHERP	10	6,69E-02	0,82	8,85E-01	0,06	8,53E-02	0,76	24,47	24,79	24,13	25,33	25,24	25,29	24,38	25,41	23,78	J3QK89;Q8IWX8	17,8	104,93	510
T-complex protein 1 subunit zeta	CCT6A	5	6,97E-01	0,33	5,98E-01	-0,44	3,68E-01	0,77	20,54	22,53	20,61	22,15	22,04	20,47	21,00	22,11	19,24	P40227	14,1	58,024	786
Nicotinamide/nicotinic acid mononucleotide adenylyltransferase 1	NMNAT1	3	5,20E-01	0,33	4,03E-01	-0,44	1,56E-01	0,77	20,26	19,98	19,93	20,27	20,42	20,48	20,67	19,84	18,36	Q9HAN9	12,2	31,932	1286
THO complex subunit 2	THOC2	22	7,65E-01	0,09	4,50E-02	-0,68	2,70E-02	0,77	25,17	25,14	25,17	25,39	25,26	25,09	24,58	24,94	23,91	Q8NI27;A0A0C4DG98	17	182,77	1151
Mitotic spindle assembly checkpoint protein MAD1	MAD1L1	3	7,47E-01	-0,23	1,00E+00	-1,00	2,84E-01	0,77	20,76	20,87	20,17	19,01	22,00	20,11	19,22	20,38	19,21	Q9Y6D9;C9JTA2	5,6	83,066	1404
Fatty acid synthase;[Acyl-carrier-protein] S-acetyltransferase;[Acyl-carrier-protein] S-malonyltransferase;3-oxoacyl-[acyl-carrier-protein] synthase;3-oxoacyl-[acyl-carrier-protein] reductase;3-hydroxyacyl-[acyl-carrier-protein] dehydratase;Enoyl-[acyl-carrier-protein] reductase;Oleoyl-[acyl-carrier-protein] hydrolase	FASN	5	7,40E-01	0,22	4,25E-01	-0,55	2,69E-01	0,77	19,50	21,38	19,10	20,90	20,54	19,21	18,84	20,12	19,38	A0A0U1RQF0;P49327	2,6	273,2	105
Probable helicase with zinc finger domain	HELZ	2	1,38E-02	0,87	1,00E+00	0,09	2,37E-02	0,78	17,12	17,34	17,34	18,40	17,63	18,37	17,34	17,63	17,11	J3QS41;P42694	1,3	219,05	515
Chromosome alignment-maintaining phosphoprotein 1	CHAMP1	5	1,86E-02	0,84	8,66E-01	0,05	2,48E-02	0,79	21,34	21,98	21,81	22,46	22,57	22,62	21,67	22,24	21,38	Q96JM3	7,5	89,098	1213
Splicing regulatory glutamine/lysine-rich protein 1	SREK1	3	8,23E-02	0,69	1,00E+00	-0,11	5,05E-02	0,80	19,71	20,82	20,16	20,59	20,88	21,30	20,52	20,06	19,79	Q8WXA9	10,2	59,38	1163
LIM and calponin homology domains-containing protein 1	LIMCH1	4	1,77E-01	0,46	1,00E+00	-0,34	3,18E-02	0,80	20,32	19,86	20,04	20,66	20,62	20,33	19,35	20,38	19,47	D6RD46;Q9UPQ0;E7EPK0;E7	7	109,93	289
rRNA 2'-O-methyltransferase fibrillarin	FBL	7	4,81E-01	0,36	3,90E-01	-0,45	1,36E-01	0,81	25,02	25,21	24,21	25,20	25,15	25,17	25,53	24,11	23,46	P22087;M0R299;M0QXL5;	35,8	33,784	730
Unconventional myosin-Ic	MYO1C	12	1,41E-01	1,14	6,52E-01	0,33	2,81E-01	0,81	23,27	19,90	21,88	23,04	22,56	22,87	22,56	21,72	21,76	F5H6E2;O00159	17,2	118,99	378
Katanin p60 ATPase-containing subunit A1	KATNA1	4	1,16E-01	0,99	7,78E-01	0,17	1,82E-01	0,83	20,51	19,23	18,91	20,41	20,58	20,64	18,47	20,65	20,03	O75449	10,4	55,964	612
ATP synthase subunit alpha, mitochondrial	ATP5A1	4	4,88E-01	0,35	1,00E+00	-0,48	1,18E-01	0,83	20,91	21,12	20,84	21,54	20,66	21,71	19,44	21,39	20,59	P25705;K7EQH4;K7EJP1;K7	11,8	59,75	742
Forkhead box protein K1	FOXK1	2	6,90E-02	0,56	1,00E+00	-0,28	1,29E-02	0,84	21,43	21,49	21,34	21,76	21,98	22,20	20,96	21,60	20,86	P85037;U3KQ26	3,5	75,456	899
Unconventional myosin-Ie	MYO1E	22	5,39E-02	1,37	4,12E-01	0,53	2,09E-01	0,84	25,94	23,66	23,41	25,88	25,74	25,52	25,58	24,37	24,66	Q12965	27,8	127,06	941
Pre-mRNA 3'-end-processing factor FIP1	FIP1L1	7	1,24E-02	0,79	8,54E-01	-0,05	9,13E-03	0,84	23,07	23,32	23,08	23,69	24,14	24,03	23,43	23,02	22,87	Q6UN15;A0A0B4J203	15,8	66,526	1081
TFIIH basal transcription factor complex helicase XPB subunit	ERCC3	5	1,47E-01	0,77	8,74E-01	-0,08	1,14E-01	0,85	21,15	21,76	21,47	21,37	22,73	22,57	20,95	22,34	20,85	A0A2R8Y6W8;P19447	9,5	91,957	130
60S ribosomal protein L27	RPL27	4	4,76E-01	0,35	3,25E-01	-0,50	1,08E-01	0,85	25,06	23,85	24,71	25,03	24,82	24,84	24,92	23,06	24,16	P61353;K7ELC7	36	15,798	853
Myosin-10	MYH10	16	5,23E-01	0,88	9,82E-01	0,03	5,38E-01	0,85	23,85	22,50	18,43	23,48	22,81	21,14	23,46	20,57	20,85	P35580	12,3	229	771
Spectrin alpha chain, non-erythrocytic 1	SPTAN1	99	2,90E-01	-0,87	5,21E-02	-1,73	2,97E-01	0,86	28,81	28,91	28,42	28,23	27,77	27,52	28,32	27,84	24,77	A0A0D9SGF6;Q13813;A0A0	50,9	287,6	93
Poly [ADP-ribose] polymerase 9	PARP9	2	9,12E-01	-0,05	8,14E-02	-0,93	9,77E-02	0,88	21,32	21,22	21,17	20,97	21,46	21,13	20,79	21,08	19,05	Q8IXQ6	3,3	96,342	1130
Histone H4	HIST1H4A	5	4,36E-01	0,43	4,06E-01	-0,46	1,25E-01	0,88	24,85	23,41	25,56	25,16	24,69	25,24	24,44	24,50	23,51	P62805	47,6	11,367	878
AP-2 complex subunit beta	AP2B1	9	4,74E-01	0,34	2,69E-01	-0,54	8,56E-02	0,89	22,60	22,75	22,39	22,60	22,87	23,31	22,56	22,68	20,89	A0A087X2S3;P63010;A0A0	14,1	101,33	49
Heat shock protein beta-1	HSPB1	7	1,11E-02	0,70	4,30E-01	-0,18	3,01E-03	0,89	24,48	24,60	24,37	25,19	25,29	25,07	24,38	24,37	24,13	P04792;F8WE04	44,9	22,782	662
60S ribosomal protein L3	RPL3	9	7,35E-02	0,93	9,48E-01	0,03	8,19E-02	0,90	24,71	24,82	23,72	25,27	25,42	25,35	25,46	24,11	23,78	P39023;G5E9G0;H7C3M2;B	30	46,108	784
TAR DNA-binding protein 43	TARDBP;TDP43	4	2,59E-01	0,37	1,19E-01	-0,53	1,66E-02	0,91	22,94	23,30	22,84	23,18	23,52	23,50	21,89	22,88	22,71	A0A087WX29;Q13148;K7EI	30,9	26,743	29
Origin recognition complex subunit 5	ORC5	2	1,62E-01	0,81	8,54E-01	-0,10	1,21E-01	0,91	18,08	18,19	18,81	18,43	19,42	19,66	17,42	19,42	17,94	O43913;G3V0H0	5,3	50,282	590
ATP-dependent RNA helicase DHX8	DHX8	3	2,89E-03	0,97	8,34E-01	0,05	4,03E-03	0,92	19,38	19,22	19,38	20,32	20,02	20,55	19,37	19,54	19,22	F5H658;Q14562	4,4	134	376
Ubiquitin-associated protein 2	UBAP2	5	2,62E-01	0,46	2,59E-01	-0,46	3,95E-02	0,92	21,20	21,95	21,92	22,15	21,86	22,43	20,47	21,75	21,48	Q5T6F2;A2A305	7,1	117,11	1054
Heterogeneous nuclear ribonucleoprotein M	HNRNPM	23	3,96E-02	0,69	4,18E-01	-0,24	9,79E-03	0,93	26,72	27,01	26,43	27,19	27,42	27,62	26,62	26,77	26,04	A0A087X0X3;P52272;M0R	46,7	77,569	45
Zinc finger protein 787	ZNF787	2	1,27E-01	0,86	8,49E-01	-0,10	9,31E-02	0,96	20,41	21,76	19,51	21,17	21,17	21,92	20,33	20,73	20,32	Q6DD87	6,8	40,428	1071
40S ribosomal protein S6	RPS6	5	6,60E-02	0,91	8,98E-01	-0,06	5,32E-02	0,96	22,86	23,99	24,20	24,53	24,29	24,95	23,81	22,94	24,14	P62753;A2A3R7;A2A3R5	23,3	28,68	877
Myosin phosphatase Rho-interacting protein	MPRIP	19	5,16E-01	-0,47	6,93E-02	-1,44	2,00E-01	0,96	24,47	24,60	23,91	24,20	23,80	23,57	24,26	23,41	21,00	Q6WCQ1;J3KSW8	26	116,53	1084
Procollagen-lysine,2-oxoglutarate 5-dioxygenase 1	PLOD1	6	1,85E-01	0,54	2,82E-01	-0,43	2,94E-02	0,97	21,33	22,45	22,22	22,09	22,64	22,88	21,17	21,96	21,59	Q02809	10,6	83,549	911
60S ribosomal protein L18	RPL18	4	3,58E-02	0,90	8,65E-01	-0,06	2,67E-02	0,97	23,94	24,03	23,18	24,75	24,50	24,61	24,18	23,84	22,95	G3V203;H0YHA7;J3QQ67;Q	29,3	18,756	419

Supplementary table 1 Legend

	Statistically enriched with system 2
	Statistically enriched with system 1a
	Statistically enriched with system 8
	missing in all replicates of the condition, imputed with low value
	missing in one or two replicates of the condition, imputed with slsa algorithm
	Manually invalidated (found differentially abundant but contains two imputed values in the condition in which it was found more abundant)

Supplementary Table 2

Gene ID	Mapped IDs	Gene Name	Go database MF Complete
HUMAN|HGNC=17066|UniProtKB=Q9UKV3	ACIN1	Apoptotic chromatin condensation inducer in the nucleus;ACIN1;ortholog	nucleic acid binding(GO:0003676);RNA binding(GO:0003723);protein binding(GO:0005515);ATPase activity(GO:0016887);enzyme binding(GO:0019899)
HUMAN|HGNC=24124|UniProtKB=O96019	ACTL6A	Actin-like protein 6A;ACTL6A;ortholog	RNA polymerase II proximal promoter sequence-specific DNA binding(GO:0000978);RNA polymerase II distal enhancer sequence-specific DNA binding(GO:0000980);chromatin binding(GO:0003682);transcription coactivator activity(GO:0003713);protein binding(GO:0005515);nucleosomal DNA binding(GO:0031492)
HUMAN|HGNC=225|UniProtKB=P55265	ADAR	Double-stranded RNA-specific adenosine deaminase;ADAR;ortholog	DNA binding(GO:0003677);RNA binding(GO:0003723);double-stranded RNA binding(GO:0003725);double-stranded RNA adenosine deaminase activity(GO:0003726);adenosine deaminase activity(GO:0004000);protein binding(GO:0005515);tRNA-specific adenosine deaminase activity(GO:0008251);metal ion binding(GO:0046872)
HUMAN|HGNC=10648|UniProtKB=Q12904	AIMP1	Aminoacyl tRNA synthase complex-interacting multifunctional protein 1;AIMP1;ortholog	tRNA binding(GO:0000049);cytokine activity(GO:0005125);protein binding(GO:0005515);protein homodimerization activity(GO:0042803);GTPase binding(GO:0051020)
HUMAN|HGNC=20609|UniProtKB=Q13155	AIMP2	Aminoacyl tRNA synthase complex-interacting multifunctional protein 2;AIMP2;ortholog	protein binding(GO:0005515);protein-containing complex scaffold activity(GO:0032947)
HUMAN|HGNC=371|UniProtKB=Q12802	AKAP13	A-kinase anchor protein 13;AKAP13;ortholog	cAMP-dependent protein kinase activity(GO:0004691);MAP-kinase scaffold activity(GO:0005078);Rho guanyl-nucleotide exchange factor activity(GO:0005089);protein binding(GO:0005515);Rho GTPase binding(GO:0017048);protein-containing complex scaffold activity(GO:0032947);metal ion binding(GO:0046872);protein kinase A binding(GO:0051018)
HUMAN|HGNC=25996|UniProtKB=Q6P6C2	ALKBH5	RNA demethylase ALKBH5;ALKBH5;ortholog	RNA binding(GO:0003723);2-oxoglutarate-dependent dioxygenase activity(GO:0016706);metal ion binding(GO:0046872);RNA N6-methyladenosine dioxygenase activity(GO:1990931)
HUMAN|HGNC=24714|UniProtKB=Q8IWZ3	ANKHD1	Ankyrin repeat and KH domain-containing protein 1;ANKHD1;ortholog	RNA binding(GO:0003723);protein binding(GO:0005515)
HUMAN|HGNC=14082|UniProtKB=Q9NQW6	ANLN	Anillin;ANLN;ortholog	actin binding(GO:0003779);protein binding(GO:0005515);GTP-Rho binding(GO:0017049);cadherin binding(GO:0045296)
HUMAN|HGNC=568|UniProtKB=O14617	AP3D1	AP-3 complex subunit delta-1;AP3D1;ortholog	transporter activity(GO:0005215)
HUMAN|HGNC=594|UniProtKB=Q9BZZ5	API5	Apoptosis inhibitor 5;API5;ortholog	RNA binding(GO:0003723);protein binding(GO:0005515);fibroblast growth factor binding(GO:0017134)
HUMAN|HGNC=646|UniProtKB=P10398	ARAF	Serine/threonine-protein kinase A-Raf;ARAF;ortholog	protein kinase activity(GO:0004672);protein serine/threonine kinase activity(GO:0004674);MAP kinase kinase kinase activity(GO:0004709);protein binding(GO:0005515);ATP binding(GO:0005524);metal ion binding(GO:0046872)
HUMAN|HGNC=682|UniProtKB=Q92974	ARHGEF2	Rho guanine nucleotide exchange factor 2;ARHGEF2;ortholog	Rho guanyl-nucleotide exchange factor activity(GO:0005089);protein binding(GO:0005515);microtubule binding(GO:0008017);transcription factor binding(GO:0008134);zinc ion binding(GO:0008270);Rho GTPase binding(GO:0017048);Rac guanyl-nucleotide exchange factor activity(GO:0030676);metal ion binding(GO:0046872);Rac GTPase binding(GO:0048365)
HUMAN|HGNC=15607|UniProtKB=Q14155	ARHGEF7	Rho guanine nucleotide exchange factor 7;ARHGEF7;ortholog	guanyl-nucleotide exchange factor activity(GO:0005085);Rho guanyl-nucleotide exchange factor activity(GO:0005089);protein binding(GO:0005515);protein kinase binding(GO:0019901)
HUMAN|HGNC=10555|UniProtKB=Q99700	ATXN2	Ataxin-2;ATXN2;ortholog	RNA binding(GO:0003723);epidermal growth factor receptor binding(GO:0005154);protein binding(GO:0005515);protein C-terminus binding(GO:0008022)
HUMAN|HGNC=31326|UniProtKB=Q8WWM7	ATXN2L	Ataxin-2-like protein;ATXN2L;ortholog	RNA binding(GO:0003723);protein binding(GO:0005515);cadherin binding(GO:0045296)
HUMAN|HGNC=11393|UniProtKB=O14965	AURKA	Aurora kinase A;AURKA;ortholog	protein kinase activity(GO:0004672);protein serine/threonine kinase activity(GO:0004674);protein serine/threonine/tyrosine kinase activity(GO:0004712);protein binding(GO:0005515);ATP binding(GO:0005524);protein kinase binding(GO:0019901);ubiquitin protein ligase binding(GO:0031625);histone serine kinase activity(GO:0035174);protein heterodimerization activity(GO:0046982)
HUMAN|HGNC=937|UniProtKB=Q99933	BAG1	BAG family molecular chaperone regulator 1;BAG1;ortholog	adenyl-nucleotide exchange factor activity(GO:0000774);protein binding(GO:0005515);ubiquitin protein ligase binding(GO:0031625);chaperone binding(GO:0051087)
HUMAN|HGNC=938|UniProtKB=O95816	BAG2	BAG family molecular chaperone regulator 2;BAG2;ortholog	adenyl-nucleotide exchange factor activity(GO:0000774);protein binding(GO:0005515);heat shock protein binding(GO:0031072);ubiquitin protein ligase binding(GO:0031625);identical protein binding(GO:0042802);ion channel binding(GO:0044325);tau protein binding(GO:0048156);chaperone binding(GO:0051087)
HUMAN|HGNC=971|UniProtKB=P56945	BCAR1	Breast cancer anti-estrogen resistance protein 1;BCAR1;ortholog	protein binding(GO:0005515);SH3 domain binding(GO:0017124);protein kinase binding(GO:0019901)
HUMAN|HGNC=13575|UniProtKB=O60885	BRD4	Bromodomain-containing protein 4;BRD4;ortholog	transcription regulator recruiting activity(GO:0001134);p53 binding(GO:0002039);chromatin binding(GO:0003682);protein binding(GO:0005515);RNA polymerase II CTD heptapeptide repeat kinase activity(GO:0008353);enzyme binding(GO:0019899);lysine-acetylated histone binding(GO:0070577);RNA polymerase II C-terminal domain binding(GO:0099122);P-TEFb complex binding(GO:0106140)
HUMAN|HGNC=1151|UniProtKB=O43684	BUB3	Mitotic checkpoint protein BUB3;BUB3;ortholog	protein binding(GO:0005515);ubiquitin binding(GO:0043130)
HUMAN|HGNC=20127|UniProtKB=Q86SX3	c14orf80	Tubulin epsilon and delta complex protein 1;TEDC1;ortholog	protein binding(GO:0005515)
HUMAN|HGNC=1424|UniProtKB=P27708	CAD	CAD protein;CAD;ortholog	aspartate carbamoyltransferase activity(GO:0004070);carbamoyl-phosphate synthase (glutamine-hydrolyzing) activity(GO:0004088);dihydroorotase activity(GO:0004151);protein kinase activity(GO:0004672);ATP binding(GO:0005524);zinc ion binding(GO:0008270);amino acid binding(GO:0016597);carboxyl- or carbamoyltransferase activity(GO:0016743);hydrolase activity, acting on carbon-nitrogen (but not peptide) bonds(GO:0016810);hydrolase activity, acting on carbon-nitrogen (but not peptide) bonds, in cyclic amides(GO:0016812);enzyme binding(GO:0019899);identical protein binding(GO:0042802);metal ion binding(GO:0046872);aspartate binding(GO:0070335)
HUMAN|HGNC=1462|UniProtKB=Q13557	CAMK2D	Calcium/calmodulin-dependent protein kinase type II subunit delta;CAMK2D;ortholog	protein serine/threonine kinase activity(GO:0004674);calmodulin-dependent protein kinase activity(GO:0004683);protein binding(GO:0005515);calmodulin binding(GO:0005516);ATP binding(GO:0005524);sodium channel inhibitor activity(GO:0019871);titin binding(GO:0031432);identical protein binding(GO:0042802);protein homodimerization activity(GO:0042803);ion channel binding(GO:0044325)
HUMAN|HGNC=1463|UniProtKB=Q13555	CAMK2G	Calcium/calmodulin-dependent protein kinase type II subunit gamma;CAMK2G;ortholog	calmodulin-dependent protein kinase activity(GO:0004683);calcium-dependent protein serine/threonine phosphatase activity(GO:0004723);protein binding(GO:0005515);calmodulin binding(GO:0005516);ATP binding(GO:0005524);identical protein binding(GO:0042802);protein homodimerization activity(GO:0042803)
HUMAN|HGNC=29188|UniProtKB=Q08AD1	CAMSAP2	Calmodulin-regulated spectrin-associated protein 2;CAMSAP2;ortholog	protein binding(GO:0005515);calmodulin binding(GO:0005516);microtubule binding(GO:0008017);spectrin binding(GO:0030507);microtubule minus-end binding(GO:0051011)
HUMAN|HGNC=30237|UniProtKB=Q6P1N0	CC2D1A	Coiled-coil and C2 domain-containing protein 1A;CC2D1A;ortholog	RNA polymerase II proximal promoter sequence-specific DNA binding(GO:0000978);DNA-binding transcription repressor activity, RNA polymerase II-specific(GO:0001227);protein binding(GO:0005515);cadherin binding(GO:0045296)
HUMAN|HGNC=24236|UniProtKB=Q8IX12	CCAR1	Cell division cycle and apoptosis regulator protein 1;CCAR1;ortholog	RNA polymerase II distal enhancer sequence-specific DNA binding(GO:0000980);transcription coactivator activity(GO:0003713);transcription corepressor activity(GO:0003714);RNA binding(GO:0003723);protein binding(GO:0005515);nuclear receptor transcription coactivator activity(GO:0030374)
HUMAN|HGNC=23360|UniProtKB=Q8N163	CCAR2	Cell cycle and apoptosis regulator protein 2;CCAR2;ortholog	RNA polymerase II complex binding(GO:0000993);RNA binding(GO:0003723);enzyme inhibitor activity(GO:0004857);protein binding(GO:0005515);enzyme binding(GO:0019899);nuclear receptor transcription coactivator activity(GO:0030374)
HUMAN|HGNC=18111|UniProtKB=Q8IVM0	CCDC50	Coiled-coil domain-containing protein 50;CCDC50;ortholog	protein binding(GO:0005515);ubiquitin protein ligase binding(GO:0031625)
HUMAN|HGNC=1728|UniProtKB=P30260	CDC27	Cell division cycle protein 27 homolog;CDC27;ortholog	protein binding(GO:0005515);protein phosphatase binding(GO:0019903)
HUMAN|HGNC=1743|UniProtKB=Q99459	CDC5L	Cell division cycle 5-like protein;CDC5L;ortholog	RNA polymerase II regulatory region sequence-specific DNA binding(GO:0000977);DNA-binding transcription factor activity, RNA polymerase II-specific(GO:0000981);transcription corepressor binding(GO:0001222);DNA-binding transcription activator activity, RNA polymerase II-specific(GO:0001228);DNA binding(GO:0003677);RNA binding(GO:0003723);protein binding(GO:0005515);protein phosphatase 1 binding(GO:0008157);protein kinase binding(GO:0019901);leucine zipper domain binding(GO:0043522);WD40-repeat domain binding(GO:0071987)
HUMAN|HGNC=16783|UniProtKB=Q6P1J9	CDC73	Parafibromin;CDC73;ortholog	RNA polymerase II complex binding(GO:0000993);protein binding(GO:0005515)
HUMAN|HGNC=1771|UniProtKB=P24941	CDK2	Cyclin-dependent kinase 2;CDK2;ortholog	magnesium ion binding(GO:0000287);protein kinase activity(GO:0004672);protein serine/threonine kinase activity(GO:0004674);cyclin-dependent protein serine/threonine kinase activity(GO:0004693);protein binding(GO:0005515);ATP binding(GO:0005524);protein domain specific binding(GO:0019904);cyclin binding(GO:0030332);histone kinase activity(GO:0035173);cyclin-dependent protein kinase activity(GO:0097472)
HUMAN|HGNC=18673|UniProtKB=Q96JB5	CDK5RAP3	CDK5 regulatory subunit-associated protein 3;CDK5RAP3;ortholog	protein binding(GO:0005515);protein kinase binding(GO:0019901);cyclin binding(GO:0030332);ubiquitin-like protein ligase binding(GO:0044389);mitogen-activated protein kinase binding(GO:0051019);NF-kappaB binding(GO:0051059);MDM2/MDM4 family protein binding(GO:0097371)
HUMAN|HGNC=1778|UniProtKB=P50613	CDK7	Cyclin-dependent kinase 7;CDK7;ortholog	transcription coactivator activity(GO:0003713);protein kinase activity(GO:0004672);protein serine/threonine kinase activity(GO:0004674);cyclin-dependent protein serine/threonine kinase activity(GO:0004693);protein binding(GO:0005515);ATP binding(GO:0005524);protein C-terminus binding(GO:0008022);DNA-dependent ATPase activity(GO:0008094);RNA polymerase II CTD heptapeptide repeat kinase activity(GO:0008353);kinase activity(GO:0016301);androgen receptor binding(GO:0050681)
HUMAN|HGNC=28920|UniProtKB=Q5SW79	CEP170	Centrosomal protein of 170 kDa;CEP170;ortholog	protein binding(GO:0005515)
HUMAN|HGNC=24511|UniProtKB=Q9Y3Y2	CHTOP	Chromatin target of PRMT1 protein;CHTOP;ortholog	RNA binding(GO:0003723);protein binding(GO:0005515);methyl-CpG binding(GO:0008327)
HUMAN|HGNC=1985|UniProtKB=O14578	CIT	Citron Rho-interacting kinase;CIT;ortholog	protein kinase activity(GO:0004672);protein serine/threonine kinase activity(GO:0004674);protein binding(GO:0005515);ATP binding(GO:0005524);SH3 domain binding(GO:0017124);PDZ domain binding(GO:0030165);metal ion binding(GO:0046872);scaffold protein binding(GO:0097110)
HUMAN|HGNC=17088|UniProtKB=Q7Z460	CLASP1	CLIP-associating protein 1;CLASP1;ortholog	dystroglycan binding(GO:0002162);protein binding(GO:0005515);microtubule binding(GO:0008017);kinetochore binding(GO:0043515);microtubule plus-end binding(GO:0051010)
HUMAN|HGNC=17078|UniProtKB=O75122	CLASP2	CLIP-associating protein 2;CLASP2;ortholog	dystroglycan binding(GO:0002162);protein binding(GO:0005515);microtubule binding(GO:0008017);kinetochore binding(GO:0043515);microtubule plus-end binding(GO:0051010);actin filament binding(GO:0051015);protein tyrosine kinase binding(GO:1990782)
HUMAN|HGNC=23186|UniProtKB=Q14677	CLINT1	Clathrin interactor 1;CLINT1;ortholog	protein binding(GO:0005515);lipid binding(GO:0008289);clathrin binding(GO:0030276);cadherin binding(GO:0045296)
HUMAN|HGNC=18290|UniProtKB=Q8NFW8	CMAS	N-acylneuraminate cytidylyltransferase;CMAS;ortholog	N-acylneuraminate cytidylyltransferase activity(GO:0008781)
HUMAN|HGNC=13164|UniProtKB=P62633	CNBP	Cellular nucleic acid-binding protein;CNBP;ortholog	RNA polymerase II regulatory region sequence-specific DNA binding(GO:0000977);DNA-binding transcription repressor activity, RNA polymerase II-specific(GO:0001227);nucleic acid binding(GO:0003676);DNA-binding transcription factor activity(GO:0003700);RNA binding(GO:0003723);protein binding(GO:0005515);zinc ion binding(GO:0008270)
HUMAN|HGNC=7877|UniProtKB=A5YKK6	CNOT1	CCR4-NOT transcription complex subunit 1;CNOT1;ortholog	RNA binding(GO:0003723);poly(A)-specific ribonuclease activity(GO:0004535);protein binding(GO:0005515);protein domain specific binding(GO:0019904);estrogen receptor binding(GO:0030331);protein-containing complex scaffold activity(GO:0032947);retinoic acid receptor binding(GO:0042974);armadillo repeat domain binding(GO:0070016)
HUMAN|HGNC=23817|UniProtKB=Q9H9A5	CNOT10	CCR4-NOT transcription complex subunit 10;CNOT10;ortholog	protein binding(GO:0005515)
HUMAN|HGNC=14101|UniProtKB=Q9UIV1	CNOT7	CCR4-NOT transcription complex subunit 7;CNOT7;ortholog	3'-5'-exoribonuclease activity(GO:0000175);nucleic acid binding(GO:0003676);transcription corepressor activity(GO:0003714);RNA binding(GO:0003723);exoribonuclease activity(GO:0004532);poly(A)-specific ribonuclease activity(GO:0004535);protein binding(GO:0005515);transcription factor binding(GO:0008134);metal ion binding(GO:0046872)
HUMAN|HGNC=2230|UniProtKB=P53621	COPA	Coatomer subunit alpha;COPA;ortholog	hormone activity(GO:0005179);structural molecule activity(GO:0005198)
HUMAN|HGNC=2232|UniProtKB=P35606	COPB2	Coatomer subunit beta';COPB2;ortholog	structural molecule activity(GO:0005198);protein binding(GO:0005515)
HUMAN|HGNC=2323|UniProtKB=P31327	CPS1	Carbamoyl-phosphate synthase [ammonia], mitochondrial;CPS1;ortholog	carbamoyl-phosphate synthase (ammonia) activity(GO:0004087);carbamoyl-phosphate synthase (glutamine-hydrolyzing) activity(GO:0004088);endopeptidase activity(GO:0004175);calcium ion binding(GO:0005509);protein binding(GO:0005515);ATP binding(GO:0005524);phospholipid binding(GO:0005543);glutamate binding(GO:0016595);protein-containing complex binding(GO:0044877);modified amino acid binding(GO:0072341)
HUMAN|HGNC=2324|UniProtKB=Q10570	CPSF1	Cleavage and polyadenylation specificity factor subunit 1;CPSF1;ortholog	nucleic acid binding(GO:0003676);protein binding(GO:0005515);enzyme binding(GO:0019899);mRNA 3'-UTR AU-rich region binding(GO:0035925)
HUMAN|HGNC=2325|UniProtKB=Q9P2I0	CPSF2	Cleavage and polyadenylation specificity factor subunit 2;CPSF2;ortholog	RNA binding(GO:0003723);protein binding(GO:0005515)
HUMAN|HGNC=2326|UniProtKB=Q9UKF6	CPSF3	Cleavage and polyadenylation specificity factor subunit 3;CPSF3;ortholog	RNA binding(GO:0003723);endoribonuclease activity(GO:0004521);protein binding(GO:0005515);5'-3' exonuclease activity(GO:0008409);metal ion binding(GO:0046872)
HUMAN|HGNC=2327|UniProtKB=O95639	CPSF4	Cleavage and polyadenylation specificity factor subunit 4;CPSF4;ortholog	nucleic acid binding(GO:0003676);RNA binding(GO:0003723);protein binding(GO:0005515);zinc ion binding(GO:0008270);metal ion binding(GO:0046872)
HUMAN|HGNC=15762|UniProtKB=Q9BZJ0	CRNKL1	Crooked neck-like protein 1;CRNKL1;ortholog	RNA binding(GO:0003723);protein binding(GO:0005515)
HUMAN|HGNC=2451|UniProtKB=P48729	CSNK1A1	Casein kinase I isoform alpha;CSNK1A1;ortholog	protein kinase activity(GO:0004672);protein serine/threonine kinase activity(GO:0004674);protein binding(GO:0005515);ATP binding(GO:0005524);kinase activity(GO:0016301)
HUMAN|HGNC=2483|UniProtKB=Q05048	CSTF1	Cleavage stimulation factor subunit 1;CSTF1;ortholog	RNA binding(GO:0003723);protein binding(GO:0005515)
HUMAN|HGNC=2485|UniProtKB=Q12996	CSTF3	Cleavage stimulation factor subunit 3;CSTF3;ortholog	RNA binding(GO:0003723);mRNA binding(GO:0003729);protein binding(GO:0005515)
HUMAN|HGNC=29322|UniProtKB=Q9HCG8	CWC22	Pre-mRNA-splicing factor CWC22 homolog;CWC22;ortholog	RNA binding(GO:0003723);protein binding(GO:0005515)
HUMAN|HGNC=10664|UniProtKB=Q6UX04	CWC27	Peptidyl-prolyl cis-trans isomerase CWC27 homolog;CWC27;ortholog	peptidyl-prolyl cis-trans isomerase activity(GO:0003755)
HUMAN|HGNC=13759|UniProtKB=Q7L576	CYFIP1	Cytoplasmic FMR1-interacting protein 1;CYFIP1;ortholog	RNA 7-methylguanosine cap binding(GO:0000340);protein binding(GO:0005515);translation regulator activity(GO:0045182);Rac GTPase binding(GO:0048365);actin filament binding(GO:0051015)
HUMAN|HGNC=2678|UniProtKB=P14868	DARS	Aspartate--tRNA ligase, cytoplasmic;DARS;ortholog	nucleic acid binding(GO:0003676);RNA binding(GO:0003723);aminoacylase activity(GO:0004046);aspartate-tRNA ligase activity(GO:0004815);protein binding(GO:0005515);ATP binding(GO:0005524)
HUMAN|HGNC=15594|UniProtKB=Q9UK59	DBR1	Lariat debranching enzyme;DBR1;ortholog	RNA binding(GO:0003723);RNA lariat debranching enzyme activity(GO:0008419);hydrolase activity(GO:0016787);metal ion binding(GO:0046872)
HUMAN|HGNC=30915|UniProtKB=P61962	DCAF7	DDB1- and CUL4-associated factor 7;DCAF7;ortholog	protein binding(GO:0005515)
HUMAN|HGNC=2734|UniProtKB=Q92499	DDX1	ATP-dependent RNA helicase DDX1;DDX1;ortholog	nucleic acid binding(GO:0003676);DNA binding(GO:0003677);chromatin binding(GO:0003682);transcription coregulator activity(GO:0003712);RNA binding(GO:0003723);RNA helicase activity(GO:0003724);double-stranded RNA binding(GO:0003725);helicase activity(GO:0004386);nuclease activity(GO:0004518);exonuclease activity(GO:0004527);protein binding(GO:0005515);ATP binding(GO:0005524);ATP-dependent helicase activity(GO:0008026);poly(A) binding(GO:0008143);DNA/RNA helicase activity(GO:0033677)
HUMAN|HGNC=2743|UniProtKB=Q9UHI6	DDX20	Probable ATP-dependent RNA helicase DDX20;DDX20;ortholog	DNA binding(GO:0003677);ATP-dependent RNA helicase activity(GO:0004004);protein binding(GO:0005515);ATP binding(GO:0005524);protein domain specific binding(GO:0019904);protein binding, bridging(GO:0030674);histone deacetylase binding(GO:0042826);repressing transcription factor binding(GO:0070491)
HUMAN|HGNC=17347|UniProtKB=Q9BUQ8	DDX23	Probable ATP-dependent RNA helicase DDX23;DDX23;ortholog	nucleic acid binding(GO:0003676);RNA binding(GO:0003723);ATP-dependent RNA helicase activity(GO:0004004);protein binding(GO:0005515);ATP binding(GO:0005524)
HUMAN|HGNC=2745|UniProtKB=O00571	DDX3X	ATP-dependent RNA helicase DDX3X;DDX3X;ortholog	nucleic acid binding(GO:0003676);DNA binding(GO:0003677);RNA binding(GO:0003723);GTPase activity(GO:0003924);ATP-dependent DNA helicase activity(GO:0004003);ATP-dependent RNA helicase activity(GO:0004004);helicase activity(GO:0004386);protein binding(GO:0005515);ATP binding(GO:0005524);transcription factor binding(GO:0008134);poly(A) binding(GO:0008143);eukaryotic initiation factor 4E binding(GO:0008190);ATPase activity(GO:0016887);nucleoside-triphosphatase activity(GO:0017111);translation initiation factor binding(GO:0031369);RNA strand annealing activity(GO:0033592);RNA stem-loop binding(GO:0035613);ribosomal small subunit binding(GO:0043024);CTPase activity(GO:0043273);protein serine/threonine kinase activator activity(GO:0043539);cadherin binding(GO:0045296);mRNA 5'-UTR binding(GO:0048027)
HUMAN|HGNC=18674|UniProtKB=Q9UJV9	DDX41	Probable ATP-dependent RNA helicase DDX41;DDX41;ortholog	nucleic acid binding(GO:0003676);DNA binding(GO:0003677);RNA binding(GO:0003723);helicase activity(GO:0004386);protein binding(GO:0005515);ATP binding(GO:0005524);metal ion binding(GO:0046872)
HUMAN|HGNC=18676|UniProtKB=Q86XP3	DDX42	ATP-dependent RNA helicase DDX42;DDX42;ortholog	nucleic acid binding(GO:0003676);RNA binding(GO:0003723);helicase activity(GO:0004386);protein binding(GO:0005515);ATP binding(GO:0005524)
HUMAN|HGNC=20038|UniProtKB=Q9Y2R4	DDX52	Probable ATP-dependent RNA helicase DDX52;DDX52;ortholog	RNA binding(GO:0003723);helicase activity(GO:0004386);ATP binding(GO:0005524)
HUMAN|HGNC=2747|UniProtKB=P26196	DDX6	Probable ATP-dependent RNA helicase DDX6;DDX6;ortholog	nucleic acid binding(GO:0003676);RNA binding(GO:0003723);RNA helicase activity(GO:0003724);helicase activity(GO:0004386);protein binding(GO:0005515);ATP binding(GO:0005524);protein domain specific binding(GO:0019904);cadherin binding(GO:0045296)
HUMAN|HGNC=2739|UniProtKB=O60231	DHX16	Pre-mRNA-splicing factor ATP-dependent RNA helicase DHX16;DHX16;ortholog	nucleic acid binding(GO:0003676);RNA binding(GO:0003723);RNA helicase activity(GO:0003724);helicase activity(GO:0004386);protein binding(GO:0005515);ATP binding(GO:0005524);ATPase activity(GO:0016887);ATP-dependent 3'-5' RNA helicase activity(GO:0034459)
HUMAN|HGNC=15815|UniProtKB=Q7Z478	DHX29	ATP-dependent RNA helicase DHX29;DHX29;ortholog	RNA binding(GO:0003723);translation initiation factor activity(GO:0003743);ATP-dependent RNA helicase activity(GO:0004004);ATP binding(GO:0005524);ATP-dependent 3'-5' RNA helicase activity(GO:0034459);ribosomal small subunit binding(GO:0043024);cadherin binding(GO:0045296)
HUMAN|HGNC=16716|UniProtKB=Q7L2E3	DHX30	Putative ATP-dependent RNA helicase DHX30;DHX30;ortholog	G-quadruplex RNA binding(GO:0002151);nucleic acid binding(GO:0003676);chromatin binding(GO:0003682);RNA binding(GO:0003723);double-stranded RNA binding(GO:0003725);ATP-dependent DNA helicase activity(GO:0004003);ATP-dependent RNA helicase activity(GO:0004004);helicase activity(GO:0004386);protein binding(GO:0005515);ATP binding(GO:0005524);ATP-dependent 3'-5' RNA helicase activity(GO:0034459)
HUMAN|HGNC=14410|UniProtKB=Q9H2U1	DHX36	ATP-dependent RNA helicase DHX36;DHX36;ortholog	magnesium ion binding(GO:0000287);RNA polymerase II proximal promoter sequence-specific DNA binding(GO:0000978);G-quadruplex RNA binding(GO:0002151);nucleic acid binding(GO:0003676);single-stranded DNA binding(GO:0003697);RNA binding(GO:0003723);double-stranded RNA binding(GO:0003725);mRNA 3'-UTR binding(GO:0003730);ATP-dependent DNA helicase activity(GO:0004003);ATP-dependent RNA helicase activity(GO:0004004);helicase activity(GO:0004386);protein binding(GO:0005515);ATP binding(GO:0005524);DNA-dependent ATPase activity(GO:0008094);ATP-dependent 3'-5' RNA helicase activity(GO:0034459);mRNA 3'-UTR AU-rich region binding(GO:0035925);histone deacetylase binding(GO:0042826);transcription regulatory region DNA binding(GO:0044212);mRNA 5'-UTR binding(GO:0048027);G-quadruplex DNA binding(GO:0051880);telomerase RNA binding(GO:0070034);pre-miRNA binding(GO:0070883)
HUMAN|HGNC=17211|UniProtKB=Q92620	DHX38	Pre-mRNA-splicing factor ATP-dependent RNA helicase PRP16;DHX38;ortholog	RNA binding(GO:0003723);protein binding(GO:0005515);ATP binding(GO:0005524);ATP-dependent 3'-5' RNA helicase activity(GO:0034459)
HUMAN|HGNC=18018|UniProtKB=Q8IX18	DHX40	Probable ATP-dependent RNA helicase DHX40;DHX40;ortholog	nucleic acid binding(GO:0003676);RNA binding(GO:0003723);helicase activity(GO:0004386);ATP binding(GO:0005524);ATP-dependent 3'-5' RNA helicase activity(GO:0034459)
HUMAN|HGNC=15480|UniProtKB=Q9NSV4	DIAPH3	Protein diaphanous homolog 3;DIAPH3;ortholog	actin binding(GO:0003779);Rho GTPase binding(GO:0017048);cadherin binding(GO:0045296)
HUMAN|HGNC=5229|UniProtKB=P31689	DNAJA1	DnaJ homolog subfamily A member 1;DNAJA1;ortholog	G protein-coupled receptor binding(GO:0001664);ATPase activator activity(GO:0001671);protein binding(GO:0005515);ATP binding(GO:0005524);Hsp70 protein binding(GO:0030544);Tat protein binding(GO:0030957);ubiquitin protein ligase binding(GO:0031625);metal ion binding(GO:0046872);low-density lipoprotein particle receptor binding(GO:0050750);unfolded protein binding(GO:0051082);chaperone binding(GO:0051087);C3HC4-type RING finger domain binding(GO:0055131)
HUMAN|HGNC=14884|UniProtKB=O60884	DNAJA2	DnaJ homolog subfamily A member 2;DNAJA2;ortholog	ATPase activator activity(GO:0001671);protein binding(GO:0005515);ATP binding(GO:0005524);heat shock protein binding(GO:0031072);metal ion binding(GO:0046872);unfolded protein binding(GO:0051082);chaperone binding(GO:0051087)
HUMAN|HGNC=14889|UniProtKB=Q9UBS4	DNAJB11	DnaJ homolog subfamily B member 11;DNAJB11;ortholog	protein binding(GO:0005515);unfolded protein binding(GO:0051082)
HUMAN|HGNC=30343|UniProtKB=O75165	DNAJC13	DnaJ homolog subfamily C member 13;DNAJC13;ortholog	protein binding(GO:0005515)
HUMAN|HGNC=2976|UniProtKB=P26358	DNMT1	DNA (cytosine-5)-methyltransferase 1;DNMT1;ortholog	DNA-binding transcription factor activity, RNA polymerase II-specific(GO:0000981);DNA binding(GO:0003677);RNA binding(GO:0003723);DNA (cytosine-5-)-methyltransferase activity(GO:0003886);protein binding(GO:0005515);methyltransferase activity(GO:0008168);zinc ion binding(GO:0008270);methyl-CpG binding(GO:0008327);DNA-methyltransferase activity(GO:0009008);promoter-specific chromatin binding(GO:1990841)
HUMAN|HGNC=16160|UniProtKB=Q9H147	DNTTIP1	Deoxynucleotidyltransferase terminal-interacting protein 1;DNTTIP1;ortholog	DNA binding(GO:0003677);protein binding(GO:0005515);nucleosome binding(GO:0031491);protein homodimerization activity(GO:0042803)
HUMAN|HGNC=23476|UniProtKB=Q9H7D0	DOCK5	Dedicator of cytokinesis protein 5;DOCK5;ortholog	guanyl-nucleotide exchange factor activity(GO:0005085);protein binding(GO:0005515)
HUMAN|HGNC=3061|UniProtKB=P23919	DTYMK	Thymidylate kinase;DTYMK;ortholog	nucleoside diphosphate kinase activity(GO:0004550);thymidylate kinase activity(GO:0004798);ATP binding(GO:0005524);uridylate kinase activity(GO:0009041);kinase activity(GO:0016301)
HUMAN|HGNC=2961|UniProtKB=Q14204	DYNC1H1	Cytoplasmic dynein 1 heavy chain 1;DYNC1H1;ortholog	RNA binding(GO:0003723);microtubule motor activity(GO:0003777);protein binding(GO:0005515);ATP binding(GO:0005524);ATP-dependent microtubule motor activity, minus-end-directed(GO:0008569);dynein light chain binding(GO:0045503);dynein intermediate chain binding(GO:0045505);dynein light intermediate chain binding(GO:0051959)
HUMAN|HGNC=18745|UniProtKB=Q9Y6G9	DYNC1LI1	Cytoplasmic dynein 1 light intermediate chain 1;DYNC1LI1;ortholog	RNA binding(GO:0003723);microtubule motor activity(GO:0003777);protein binding(GO:0005515);ATP binding(GO:0005524);GDP binding(GO:0019003);dynein heavy chain binding(GO:0045504)
HUMAN|HGNC=2966|UniProtKB=O43237	DYNC1LI2	Cytoplasmic dynein 1 light intermediate chain 2;DYNC1LI2;ortholog	microtubule motor activity(GO:0003777);ATP binding(GO:0005524);dynein heavy chain binding(GO:0045504)
HUMAN|HGNC=17157|UniProtKB=Q6P2E9	EDC4	Enhancer of mRNA-decapping protein 4;EDC4;ortholog	protein binding(GO:0005515)
HUMAN|HGNC=3212|UniProtKB=O43324	EEF1E1	Eukaryotic translation elongation factor 1 epsilon-1;EEF1E1;ortholog	protein binding(GO:0005515)
HUMAN|HGNC=30858|UniProtKB=Q15029	EFTUD2	116 kDa U5 small nuclear ribonucleoprotein component;EFTUD2;ortholog	RNA binding(GO:0003723);GTPase activity(GO:0003924);protein binding(GO:0005515);GTP binding(GO:0005525);U5 snRNA binding(GO:0030623)
HUMAN|HGNC=3266|UniProtKB=P20042	EIF2S2	Eukaryotic translation initiation factor 2 subunit 2;EIF2S2;ortholog	RNA binding(GO:0003723);translation initiation factor activity(GO:0003743);protein binding(GO:0005515);translation factor activity, RNA binding(GO:0008135);metal ion binding(GO:0046872)
HUMAN|HGNC=3293|UniProtKB=O60573	EIF4E2	Eukaryotic translation initiation factor 4E type 2;EIF4E2;ortholog	RNA cap binding(GO:0000339);RNA 7-methylguanosine cap binding(GO:0000340);RNA binding(GO:0003723);translation initiation factor activity(GO:0003743);protein binding(GO:0005515);translation factor activity, RNA binding(GO:0008135);ubiquitin protein ligase binding(GO:0031625)
HUMAN|HGNC=3296|UniProtKB=Q04637	EIF4G1	Eukaryotic translation initiation factor 4 gamma 1;EIF4G1;ortholog	RNA binding(GO:0003723);mRNA binding(GO:0003729);translation initiation factor activity(GO:0003743);protein binding(GO:0005515);ATP binding(GO:0005524);translation factor activity, RNA binding(GO:0008135);eukaryotic initiation factor 4E binding(GO:0008190);translation initiation factor binding(GO:0031369);protein-containing complex scaffold activity(GO:0032947);identical protein binding(GO:0042802)
HUMAN|HGNC=17233|UniProtKB=Q96JJ3	ELMO2	Engulfment and cell motility protein 2;ELMO2;ortholog	protein binding(GO:0005515);SH3 domain binding(GO:0017124);receptor tyrosine kinase binding(GO:0030971)
HUMAN|HGNC=20696|UniProtKB=Q9H9T3	ELP3	Elongator complex protein 3;ELP3;ortholog	RNA polymerase II complex binding(GO:0000993);histone acetyltransferase activity(GO:0004402);protein binding(GO:0005515);phosphorylase kinase regulator activity(GO:0008607);acetyltransferase activity(GO:0016407);metal ion binding(GO:0046872);iron-sulfur cluster binding(GO:0051536)
HUMAN|HGNC=18035|UniProtKB=O95834	EML2	Echinoderm microtubule-associated protein-like 2;EML2;ortholog	signaling receptor binding(GO:0005102);protein binding(GO:0005515);microtubule binding(GO:0008017);protein C-terminus binding(GO:0008022);tubulin binding(GO:0015631)
HUMAN|HGNC=26666|UniProtKB=Q32P44	EML3	Echinoderm microtubule-associated protein-like 3;EML3;ortholog	microtubule binding(GO:0008017)
HUMAN|HGNC=3418|UniProtKB=P07814	EPRS	Bifunctional glutamate/proline--tRNA ligase;EPRS;ortholog	glutamate-tRNA ligase activity(GO:0004818);proline-tRNA ligase activity(GO:0004827);protein binding(GO:0005515);ATP binding(GO:0005524);zinc ion binding(GO:0008270);RNA stem-loop binding(GO:0035613);identical protein binding(GO:0042802);protein homodimerization activity(GO:0042803);GTPase binding(GO:0051020)
HUMAN|HGNC=17072|UniProtKB=Q8IUD2	ERC1	ELKS/Rab6-interacting/CAST family member 1;ERC1;ortholog	protein binding(GO:0005515);Rab GTPase binding(GO:0017137);PDZ domain binding(GO:0030165);leucine zipper domain binding(GO:0043522);cadherin binding(GO:0045296)
HUMAN|HGNC=3508|UniProtKB=Q01844	EWSR1	RNA-binding protein EWS;EWSR1;ortholog	RNA binding(GO:0003723);protein binding(GO:0005515);calmodulin binding(GO:0005516);identical protein binding(GO:0042802);metal ion binding(GO:0046872)
HUMAN|HGNC=30380|UniProtKB=Q9NV70	EXOC1	Exocyst complex component 1;EXOC1;ortholog	protein binding(GO:0005515);phosphatidylinositol-4,5-bisphosphate binding(GO:0005546);GTP-Rho binding(GO:0017049)
HUMAN|HGNC=24968|UniProtKB=Q96KP1	EXOC2	Exocyst complex component 2;EXOC2;ortholog	protein binding(GO:0005515);Ral GTPase binding(GO:0017160);protein kinase binding(GO:0019901);protein N-terminus binding(GO:0047485)
HUMAN|HGNC=30389|UniProtKB=Q96A65	EXOC4	Exocyst complex component 4;EXOC4;ortholog	protein binding(GO:0005515);Ral GTPase binding(GO:0017160);PDZ domain binding(GO:0030165);protein N-terminus binding(GO:0047485)
HUMAN|HGNC=17097|UniProtKB=Q13868	EXOSC2	Exosome complex component RRP4;EXOSC2;ortholog	3'-5'-exoribonuclease activity(GO:0000175);RNA binding(GO:0003723);protein binding(GO:0005515);7S RNA binding(GO:0008312)
HUMAN|HGNC=18189|UniProtKB=Q9NPD3	EXOSC4	Exosome complex component RRP41;EXOSC4;ortholog	3'-5'-exoribonuclease activity(GO:0000175);protein binding(GO:0005515);AU-rich element binding(GO:0017091)
HUMAN|HGNC=13247|UniProtKB=Q9NZB2	FAM120A	Constitutive coactivator of PPAR-gamma-like protein 1;FAM120A;ortholog	RNA binding(GO:0003723)
HUMAN|HGNC=21109|UniProtKB=Q96EK7	FAM120B	Constitutive coactivator of peroxisome proliferator-activated receptor gamma;FAM120B;ortholog	protein binding(GO:0005515)
HUMAN|HGNC=25282|UniProtKB=Q96TA1	FAM129B	Niban-like protein 1;FAM129B;ortholog	transcription coactivator activity(GO:0003713);cadherin binding(GO:0045296)
HUMAN|HGNC=30314|UniProtKB=Q9UK61	FAM208A	Protein TASOR;FAM208A;ortholog	chromatin binding(GO:0003682);RNA binding(GO:0003723);protein binding(GO:0005515)
HUMAN|HGNC=28210|UniProtKB=Q86UY5	FAM83A	Protein FAM83A;FAM83A;ortholog	protein binding(GO:0005515);protein kinase binding(GO:0019901);phosphatidylinositol 3-kinase regulatory subunit binding(GO:0036312)
HUMAN|HGNC=32554|UniProtKB=A6NDJ6	FAM83G	Protein FAM83G;FAM83G;ortholog	protein binding(GO:0005515)
HUMAN|HGNC=24520|UniProtKB=Q8NCA5	FAM98A	Protein FAM98A;FAM98A;ortholog	RNA binding(GO:0003723);protein binding(GO:0005515);protein methyltransferase activity(GO:0008276)
HUMAN|HGNC=26773|UniProtKB=Q52LJ0	FAM98B	Protein FAM98B;FAM98B;ortholog	RNA binding(GO:0003723);protein binding(GO:0005515);protein methyltransferase activity(GO:0008276);identical protein binding(GO:0042802)
HUMAN|HGNC=13593|UniProtKB=Q8NEZ5	FBXO22	F-box only protein 22;FBXO22;ortholog	ubiquitin-protein transferase activity(GO:0004842);protein binding(GO:0005515)
HUMAN|HGNC=3676|UniProtKB=P09038	FGF2	Fibroblast growth factor 2;FGF2;ortholog	fibroblast growth factor receptor binding(GO:0005104);cytokine activity(GO:0005125);integrin binding(GO:0005178);protein binding(GO:0005515);growth factor activity(GO:0008083);heparin binding(GO:0008201);nuclear receptor transcription coactivator activity(GO:0030374);chemoattractant activity(GO:0042056);receptor-receptor interaction(GO:0090722)
HUMAN|HGNC=17905|UniProtKB=Q9Y613	FHOD1	FH1/FH2 domain-containing protein 1;FHOD1;ortholog	actin binding(GO:0003779);protein binding(GO:0005515);protein domain specific binding(GO:0019904);identical protein binding(GO:0042802);protein self-association(GO:0043621)
HUMAN|HGNC=3754|UniProtKB=P21333	FLNA	Filamin-A;FLNA;ortholog	G protein-coupled receptor binding(GO:0001664);RNA binding(GO:0003723);protein binding(GO:0005515);transcription factor binding(GO:0008134);potassium channel regulator activity(GO:0015459);Rho GTPase binding(GO:0017048);Ral GTPase binding(GO:0017160);kinase binding(GO:0019900);small GTPase binding(GO:0031267);mu-type opioid receptor binding(GO:0031852);Fc-gamma receptor I complex binding(GO:0034988);protein homodimerization activity(GO:0042803);ion channel binding(GO:0044325);cadherin binding(GO:0045296);SMAD binding(GO:0046332);Rac GTPase binding(GO:0048365);actin filament binding(GO:0051015);GTPase binding(GO:0051020)
HUMAN|HGNC=19752|UniProtKB=Q8N3X1	FNBP4	Formin-binding protein 4;FNBP4;ortholog	protein binding(GO:0005515)
HUMAN|HGNC=4004|UniProtKB=Q96AE4	FUBP1	Far upstream element-binding protein 1;FUBP1;ortholog	single-stranded DNA binding(GO:0003697);DNA-binding transcription factor activity(GO:0003700);RNA binding(GO:0003723);protein binding(GO:0005515)
HUMAN|HGNC=4005|UniProtKB=Q96I24	FUBP3	Far upstream element-binding protein 3;FUBP3;ortholog	RNA polymerase II proximal promoter sequence-specific DNA binding(GO:0000978);DNA-binding transcription activator activity, RNA polymerase II-specific(GO:0001228);RNA binding(GO:0003723);protein binding(GO:0005515)
HUMAN|HGNC=4010|UniProtKB=P35637	FUS	RNA-binding protein FUS;FUS;ortholog	DNA binding(GO:0003677);chromatin binding(GO:0003682);transcription coactivator activity(GO:0003713);RNA binding(GO:0003723);protein binding(GO:0005515);estrogen receptor binding(GO:0030331);myosin V binding(GO:0031489);ionotropic glutamate receptor binding(GO:0035255);identical protein binding(GO:0042802);metal ion binding(GO:0046872);retinoid X receptor binding(GO:0046965);thyroid hormone receptor binding(GO:0046966)
HUMAN|HGNC=30292|UniProtKB=Q13283	G3BP1	Ras GTPase-activating protein-binding protein 1;G3BP1;ortholog	DNA binding(GO:0003677);RNA binding(GO:0003723);mRNA binding(GO:0003729);ATP-dependent DNA helicase activity(GO:0004003);ATP-dependent RNA helicase activity(GO:0004004);endonuclease activity(GO:0004519);protein binding(GO:0005515);ATP binding(GO:0005524)
HUMAN|HGNC=30291|UniProtKB=Q9UN86	G3BP2	Ras GTPase-activating protein-binding protein 2;G3BP2;ortholog	RNA binding(GO:0003723);mRNA binding(GO:0003729);protein binding(GO:0005515);receptor signaling complex scaffold activity(GO:0030159)
HUMAN|HGNC=9126|UniProtKB=O75420	GIGYF1	GRB10-interacting GYF protein 1;GIGYF1;ortholog	protein binding(GO:0005515)
HUMAN|HGNC=11960|UniProtKB=Q6Y7W6	GIGYF2	GRB10-interacting GYF protein 2;GIGYF2;ortholog	RNA binding(GO:0003723);protein binding(GO:0005515);cadherin binding(GO:0045296);proline-rich region binding(GO:0070064)
HUMAN|HGNC=4272|UniProtKB=Q9Y2X7	GIT1	ARF GTPase-activating protein GIT1;GIT1;ortholog	GTPase activator activity(GO:0005096);protein binding(GO:0005515);protein-containing complex binding(GO:0044877);metal ion binding(GO:0046872)
HUMAN|HGNC=4426|UniProtKB=Q08378	GOLGA3	Golgin subfamily A member 3;GOLGA3;ortholog	transporter activity(GO:0005215);protein binding(GO:0005515);cadherin binding(GO:0045296)
HUMAN|HGNC=30677|UniProtKB=Q92917	GPKOW	G-patch domain and KOW motifs-containing protein;GPKOW;ortholog	RNA binding(GO:0003723);protein binding(GO:0005515)
HUMAN|HGNC=4655|UniProtKB=P32780	GTF2H1	General transcription factor IIH subunit 1;GTF2H1;ortholog	chromatin binding(GO:0003682);protein kinase activity(GO:0004672);protein binding(GO:0005515);DNA-dependent ATPase activity(GO:0008094);RNA polymerase II CTD heptapeptide repeat kinase activity(GO:0008353)
HUMAN|HGNC=31394|UniProtKB=Q6P1K8	GTF2H2C	General transcription factor IIH subunit 2-like protein;GTF2H2C;ortholog	nucleic acid binding(GO:0003676);protein binding(GO:0005515);zinc ion binding(GO:0008270)
HUMAN|HGNC=4658|UniProtKB=Q92759	GTF2H4	General transcription factor IIH subunit 4;GTF2H4;ortholog	ATPase activator activity(GO:0001671);double-stranded DNA binding(GO:0003690);protein kinase activity(GO:0004672);protein binding(GO:0005515);DNA-dependent ATPase activity(GO:0008094);RNA polymerase II CTD heptapeptide repeat kinase activity(GO:0008353);RNA polymerase II general transcription initiation factor activity(GO:0016251)
HUMAN|HGNC=4659|UniProtKB=P78347	GTF2I	General transcription factor II-I;GTF2I;ortholog	DNA binding(GO:0003677);DNA-binding transcription factor activity(GO:0003700);protein binding(GO:0005515)
HUMAN|HGNC=4839|UniProtKB=P51610	HCFC1	Host cell factor 1;HCFC1;ortholog	DNA-binding transcription activator activity, RNA polymerase II-specific(GO:0001228);chromatin binding(GO:0003682);transcription coactivator activity(GO:0003713);protein binding(GO:0005515);protein binding, bridging(GO:0030674);activating transcription factor binding(GO:0033613);identical protein binding(GO:0042802);histone acetyltransferase activity (H4-K5 specific)(GO:0043995);histone acetyltransferase activity (H4-K8 specific)(GO:0043996);cadherin binding(GO:0045296);histone acetyltransferase activity (H4-K16 specific)(GO:0046972);sequence-specific double-stranded DNA binding(GO:1990837)
HUMAN|HGNC=4853|UniProtKB=Q92769	HDAC2	Histone deacetylase 2;HDAC2;ortholog	RNA polymerase II proximal promoter sequence-specific DNA binding(GO:0000978);RNA polymerase II distal enhancer sequence-specific DNA binding(GO:0000980);RNA polymerase II repressing transcription factor binding(GO:0001103);chromatin binding(GO:0003682);RNA binding(GO:0003723);histone deacetylase activity(GO:0004407);protein binding(GO:0005515);transcription factor binding(GO:0008134);deacetylase activity(GO:0019213);enzyme binding(GO:0019899);heat shock protein binding(GO:0031072);nucleosomal DNA binding(GO:0031492);NAD-dependent histone deacetylase activity (H3-K14 specific)(GO:0032041);protein deacetylase activity(GO:0033558);histone deacetylase binding(GO:0042826);sequence-specific DNA binding(GO:0043565);NF-kappaB binding(GO:0051059);promoter-specific chromatin binding(GO:1990841)
HUMAN|HGNC=5031|UniProtKB=P09651	HNRNPA1	Heterogeneous nuclear ribonucleoprotein A1;HNRNPA1;ortholog	single-stranded DNA binding(GO:0003697);RNA binding(GO:0003723);single-stranded RNA binding(GO:0003727);mRNA binding(GO:0003729);protein binding(GO:0005515);protein domain specific binding(GO:0019904);miRNA binding(GO:0035198);pre-mRNA binding(GO:0036002);identical protein binding(GO:0042802);telomeric repeat-containing RNA binding(GO:0061752);G-rich strand telomeric DNA binding(GO:0098505)
HUMAN|HGNC=5033|UniProtKB=P22626	HNRNPA2B1	Heterogeneous nuclear ribonucleoproteins A2/B1;HNRNPA2B1;ortholog	RNA binding(GO:0003723);mRNA binding(GO:0003729);mRNA 3'-UTR binding(GO:0003730);protein binding(GO:0005515);miRNA binding(GO:0035198);identical protein binding(GO:0042802);single-stranded telomeric DNA binding(GO:0043047);pre-mRNA intronic binding(GO:0097157);G-rich strand telomeric DNA binding(GO:0098505);N6-methyladenosine-containing RNA binding(GO:1990247)
HUMAN|HGNC=24941|UniProtKB=P51991	HNRNPA3	Heterogeneous nuclear ribonucleoprotein A3;HNRNPA3;ortholog	RNA binding(GO:0003723);mRNA binding(GO:0003729);protein binding(GO:0005515);RNA transmembrane transporter activity(GO:0051033)
HUMAN|HGNC=5039|UniProtKB=P52597	HNRNPF	Heterogeneous nuclear ribonucleoprotein F;HNRNPF;ortholog	nucleic acid binding(GO:0003676);RNA binding(GO:0003723);single-stranded RNA binding(GO:0003727);protein binding(GO:0005515);TBP-class protein binding(GO:0017025)
HUMAN|HGNC=5041|UniProtKB=P31943	HNRNPH1	Heterogeneous nuclear ribonucleoprotein H;HNRNPH1;ortholog	nucleic acid binding(GO:0003676);RNA binding(GO:0003723);protein binding(GO:0005515);poly(U) RNA binding(GO:0008266)
HUMAN|HGNC=5043|UniProtKB=P31942	HNRNPH3	Heterogeneous nuclear ribonucleoprotein H3;HNRNPH3;ortholog	RNA binding(GO:0003723);protein binding(GO:0005515)
HUMAN|HGNC=5045|UniProtKB=P14866	HNRNPL	Heterogeneous nuclear ribonucleoprotein L;HNRNPL;ortholog	RNA binding(GO:0003723);mRNA 3'-UTR binding(GO:0003730);protein binding(GO:0005515);transcription regulatory region DNA binding(GO:0044212);pre-mRNA intronic binding(GO:0097157);mRNA CDS binding(GO:1990715)
HUMAN|HGNC=5047|UniProtKB=O43390	HNRNPR	Heterogeneous nuclear ribonucleoprotein R;HNRNPR;ortholog	RNA binding(GO:0003723);mRNA binding(GO:0003729);mRNA 3'-UTR binding(GO:0003730);protein binding(GO:0005515)
HUMAN|HGNC=5233|UniProtKB=P0DMV9	HSPA1B	Heat shock 70 kDa protein 1B;HSPA1B;ortholog	virus receptor activity(GO:0001618);G protein-coupled receptor binding(GO:0001664);RNA binding(GO:0003723);signaling receptor binding(GO:0005102);protein binding(GO:0005515);ATP binding(GO:0005524);ATPase activity(GO:0016887);enzyme binding(GO:0019899);heat shock protein binding(GO:0031072);ubiquitin protein ligase binding(GO:0031625);ATPase activity, coupled(GO:0042623);histone deacetylase binding(GO:0042826);protein folding chaperone(GO:0044183);protein N-terminus binding(GO:0047485);unfolded protein binding(GO:0051082);misfolded protein binding(GO:0051787);C3HC4-type RING finger domain binding(GO:0055131)
HUMAN|HGNC=5244|UniProtKB=P38646	HSPA9	Stress-70 protein, mitochondrial;HSPA9;ortholog	RNA binding(GO:0003723);protein binding(GO:0005515);ATP binding(GO:0005524);ATPase activity(GO:0016887);heat shock protein binding(GO:0031072);ubiquitin protein ligase binding(GO:0031625);ATPase activity, coupled(GO:0042623);protein folding chaperone(GO:0044183);unfolded protein binding(GO:0051082);misfolded protein binding(GO:0051787)
HUMAN|HGNC=5330|UniProtKB=P41252	IARS	Isoleucine--tRNA ligase, cytoplasmic;IARS;ortholog	tRNA binding(GO:0000049);aminoacyl-tRNA editing activity(GO:0002161);isoleucine-tRNA ligase activity(GO:0004822);protein binding(GO:0005515);ATP binding(GO:0005524);GTPase binding(GO:0051020)
HUMAN|HGNC=17853|UniProtKB=Q9P2D0	IBTK	Inhibitor of Bruton tyrosine kinase;IBTK;ortholog	protein kinase binding(GO:0019901);protein tyrosine kinase inhibitor activity(GO:0030292)
HUMAN|HGNC=5395|UniProtKB=Q16666	IFI16	Gamma-interferon-inducible protein 16;IFI16;ortholog	RNA polymerase II proximal promoter sequence-specific DNA binding(GO:0000978);DNA-binding transcription repressor activity, RNA polymerase II-specific(GO:0001227);double-stranded DNA binding(GO:0003690);RNA binding(GO:0003723);protein binding(GO:0005515);transcription factor binding(GO:0008134);identical protein binding(GO:0042802)
HUMAN|HGNC=28866|UniProtKB=Q9NZI8	IGF2BP1	Insulin-like growth factor 2 mRNA-binding protein 1;IGF2BP1;ortholog	RNA binding(GO:0003723);mRNA binding(GO:0003729);mRNA 3'-UTR binding(GO:0003730);protein binding(GO:0005515);translation regulator activity(GO:0045182);mRNA 5'-UTR binding(GO:0048027)
HUMAN|HGNC=5958|UniProtKB=Q13123	IK	Protein Red;IK;ortholog	protein binding(GO:0005515);identical protein binding(GO:0042802)
HUMAN|HGNC=5959|UniProtKB=O95163	IKBKAP	Elongator complex protein 1;ELP1;ortholog	RNA polymerase II complex binding(GO:0000993);protein binding(GO:0005515);phosphorylase kinase regulator activity(GO:0008607)
HUMAN|HGNC=6037|UniProtKB=Q12905	ILF2	Interleukin enhancer-binding factor 2;ILF2;ortholog	DNA binding(GO:0003677);RNA binding(GO:0003723);double-stranded RNA binding(GO:0003725);protein binding(GO:0005515);ATP binding(GO:0005524);transferase activity(GO:0016740)
HUMAN|HGNC=6038|UniProtKB=Q12906	ILF3	Interleukin enhancer-binding factor 3;ILF3;ortholog	DNA binding(GO:0003677);RNA binding(GO:0003723);double-stranded RNA binding(GO:0003725);single-stranded RNA binding(GO:0003727);protein binding(GO:0005515);AU-rich element binding(GO:0017091)
HUMAN|HGNC=6402|UniProtKB=O00410	IPO5	Importin-5;IPO5;ortholog	RNA binding(GO:0003723);GTPase inhibitor activity(GO:0005095);protein binding(GO:0005515);nuclear localization sequence binding(GO:0008139);Ran GTPase binding(GO:0008536);protein transporter activity(GO:0008565)
HUMAN|HGNC=6126|UniProtKB=Q9Y4H2	IRS2	Insulin receptor substrate 2;IRS2;ortholog	insulin receptor binding(GO:0005158);protein binding(GO:0005515);protein kinase binding(GO:0019901);protein phosphatase binding(GO:0019903);protein domain specific binding(GO:0019904);phosphatidylinositol 3-kinase binding(GO:0043548);14-3-3 protein binding(GO:0071889)
HUMAN|HGNC=29300|UniProtKB=Q63ZY3	KANK2	KN motif and ankyrin repeat domain-containing protein 2;KANK2;ortholog	protein binding(GO:0005515)
HUMAN|HGNC=6215|UniProtKB=Q15046	KARS	Lysine--tRNA ligase;KARS;ortholog	tRNA binding(GO:0000049);nucleic acid binding(GO:0003676);ATP adenylyltransferase activity(GO:0003877);aminoacyl-tRNA ligase activity(GO:0004812);lysine-tRNA ligase activity(GO:0004824);protein binding(GO:0005515);ATP binding(GO:0005524);amino acid binding(GO:0016597);identical protein binding(GO:0042802);protein homodimerization activity(GO:0042803)
HUMAN|HGNC=17016|UniProtKB=O95251	KAT7	Histone acetyltransferase KAT7;KAT7;ortholog	DNA-binding transcription factor activity, RNA polymerase II-specific(GO:0000981);DNA replication origin binding(GO:0003688);histone acetyltransferase activity(GO:0004402);protein binding(GO:0005515);zinc ion binding(GO:0008270);H4 histone acetyltransferase activity(GO:0010485);histone binding(GO:0042393);transcription regulatory region DNA binding(GO:0044212)
HUMAN|HGNC=18116|UniProtKB=Q07666	KHDRBS1	KH domain-containing, RNA-binding, signal transduction-associated protein 1;KHDRBS1;ortholog	DNA binding(GO:0003677);RNA binding(GO:0003723);SH3/SH2 adaptor activity(GO:0005070);protein binding(GO:0005515);poly(A) binding(GO:0008143);poly(U) RNA binding(GO:0008266);SH3 domain binding(GO:0017124);protein domain specific binding(GO:0019904);identical protein binding(GO:0042802);protein-containing complex binding(GO:0044877)
HUMAN|HGNC=6316|UniProtKB=Q92945	KHSRP	Far upstream element-binding protein 2;KHSRP;ortholog	nucleic acid binding(GO:0003676);DNA binding(GO:0003677);RNA binding(GO:0003723);protein binding(GO:0005515);mRNA 3'-UTR AU-rich region binding(GO:0035925)
HUMAN|HGNC=29301|UniProtKB=Q9P206	KIAA1522	Uncharacterized protein KIAA1522;KIAA1522;ortholog	
HUMAN|HGNC=6392|UniProtKB=Q02241	KIF23	Kinesin-like protein KIF23;KIF23;ortholog	microtubule motor activity(GO:0003777);protein binding(GO:0005515);ATP binding(GO:0005524);microtubule binding(GO:0008017);ATPase activity(GO:0016887)
HUMAN|HGNC=6318|UniProtKB=O00139	KIF2A	Kinesin-like protein KIF2A;KIF2A;ortholog	motor activity(GO:0003774);microtubule motor activity(GO:0003777);protein binding(GO:0005515);ATP binding(GO:0005524);microtubule binding(GO:0008017);ATPase activity(GO:0016887)
HUMAN|HGNC=6393|UniProtKB=Q99661	KIF2C	Kinesin-like protein KIF2C;KIF2C;ortholog	microtubule motor activity(GO:0003777);protein binding(GO:0005515);ATP binding(GO:0005524);microtubule binding(GO:0008017);ATPase activity(GO:0016887);centromeric DNA binding(GO:0019237);microtubule plus-end binding(GO:0051010)
HUMAN|HGNC=13339|UniProtKB=O95239	KIF4A	Chromosome-associated kinesin KIF4A;KIF4A;ortholog	DNA binding(GO:0003677);microtubule motor activity(GO:0003777);protein binding(GO:0005515);ATP binding(GO:0005524);microtubule binding(GO:0008017);ATPase activity(GO:0016887)
HUMAN|HGNC=6324|UniProtKB=P33176	KIF5B	Kinesin-1 heavy chain;KIF5B;ortholog	microtubule motor activity(GO:0003777);protein binding(GO:0005515);ATP binding(GO:0005524);microtubule binding(GO:0008017);JUN kinase binding(GO:0008432);ATPase activity(GO:0016887);identical protein binding(GO:0042802);cadherin binding(GO:0045296);microtubule lateral binding(GO:0099609)
HUMAN|HGNC=25272|UniProtKB=Q8TBB5	KLHDC4	Kelch domain-containing protein 4;KLHDC4;ortholog	protein binding(GO:0005515)
HUMAN|HGNC=6490|UniProtKB=Q13751	LAMB3	Laminin subunit beta-3;LAMB3;ortholog	structural molecule activity(GO:0005198);extracellular matrix structural constituent(GO:0005201);protein binding(GO:0005515);protein-containing complex binding(GO:0044877)
HUMAN|HGNC=29531|UniProtKB=Q6PKG0	LARP1	La-related protein 1;LARP1;ortholog	RNA cap binding(GO:0000339);RNA binding(GO:0003723);mRNA 3'-UTR binding(GO:0003730);protein binding(GO:0005515);eukaryotic initiation factor 4E binding(GO:0008190);translation activator activity(GO:0008494);translation initiation factor binding(GO:0031369);cadherin binding(GO:0045296);mRNA 5'-UTR binding(GO:0048027)
HUMAN|HGNC=24320|UniProtKB=Q71RC2	LARP4	La-related protein 4;LARP4;ortholog	RNA binding(GO:0003723);protein binding(GO:0005515);poly(A) binding(GO:0008143)
HUMAN|HGNC=28987|UniProtKB=Q92615	LARP4B	La-related protein 4B;LARP4B;ortholog	RNA binding(GO:0003723);protein binding(GO:0005515)
HUMAN|HGNC=6512|UniProtKB=Q9P2J5	LARS	Leucine--tRNA ligase, cytoplasmic;LARS;ortholog	aminoacyl-tRNA editing activity(GO:0002161);glutamine-tRNA ligase activity(GO:0004819);leucine-tRNA ligase activity(GO:0004823);GTPase activator activity(GO:0005096);protein binding(GO:0005515);ATP binding(GO:0005524)
HUMAN|HGNC=6701|UniProtKB=P30533	LRPAP1	Alpha-2-macroglobulin receptor-associated protein;LRPAP1;ortholog	amyloid-beta binding(GO:0001540);signaling receptor binding(GO:0005102);protein binding(GO:0005515);heparin binding(GO:0008201);lipase binding(GO:0035473);receptor antagonist activity(GO:0048019);low-density lipoprotein particle receptor binding(GO:0050750);very-low-density lipoprotein particle receptor binding(GO:0070326)
HUMAN|HGNC=15714|UniProtKB=P42704	LRPPRC	Leucine-rich PPR motif-containing protein, mitochondrial;LRPPRC;ortholog	single-stranded DNA binding(GO:0003697);RNA binding(GO:0003723);protein binding(GO:0005515);microtubule binding(GO:0008017);ubiquitin protein ligase binding(GO:0031625);beta-tubulin binding(GO:0048487)
HUMAN|HGNC=21581|UniProtKB=Q5VZK9	LRRC16A	F-actin-uncapping protein LRRC16A;CARMIL1;ortholog	protein binding(GO:0005515);protein-containing complex binding(GO:0044877)
HUMAN|HGNC=26407|UniProtKB=Q3MHD2	LSM12	Protein LSM12 homolog;LSM12;ortholog	
HUMAN|HGNC=24489|UniProtKB=Q8ND56	LSM14A	Protein LSM14 homolog A;LSM14A;ortholog	double-stranded DNA binding(GO:0003690);RNA binding(GO:0003723);double-stranded RNA binding(GO:0003725);single-stranded RNA binding(GO:0003727)
HUMAN|HGNC=24309|UniProtKB=O95232	LUC7L3	Luc7-like protein 3;LUC7L3;ortholog	DNA binding(GO:0003677);RNA binding(GO:0003723);mRNA binding(GO:0003729);protein binding(GO:0005515)
HUMAN|HGNC=13664|UniProtKB=Q9UPN3	MACF1	Microtubule-actin cross-linking factor 1, isoforms 1/2/3/5;MACF1;ortholog	RNA binding(GO:0003723);actin binding(GO:0003779);structural molecule activity(GO:0005198);calcium ion binding(GO:0005509);protein binding(GO:0005515);microtubule binding(GO:0008017);ATPase activity(GO:0016887);cadherin binding(GO:0045296);microtubule minus-end binding(GO:0051011);actin filament binding(GO:0051015)
HUMAN|HGNC=25514|UniProtKB=Q3KQU3	MAP7D1	MAP7 domain-containing protein 1;MAP7D1;ortholog	
HUMAN|HGNC=18752|UniProtKB=Q9BPZ7	MAPKAP1	Target of rapamycin complex 2 subunit MAPKAP1;MAPKAP1;ortholog	protein binding(GO:0005515);phosphatidylinositol-4,5-bisphosphate binding(GO:0005546);phosphatidylinositol-3,4,5-trisphosphate binding(GO:0005547);Ras GTPase binding(GO:0017016);protein kinase binding(GO:0019901);phosphatidylinositol-3,4-bisphosphate binding(GO:0043325);phosphatidic acid binding(GO:0070300);phosphatidylinositol-3,5-bisphosphate binding(GO:0080025)
HUMAN|HGNC=3332|UniProtKB=Q7KZI7	MARK2	Serine/threonine-protein kinase MARK2;MARK2;ortholog	magnesium ion binding(GO:0000287);RNA binding(GO:0003723);protein serine/threonine kinase activity(GO:0004674);protein binding(GO:0005515);ATP binding(GO:0005524);lipid binding(GO:0008289);protein kinase activator activity(GO:0030295);cadherin binding(GO:0045296);tau protein binding(GO:0048156);tau-protein kinase activity(GO:0050321)
HUMAN|HGNC=6897|UniProtKB=P27448	MARK3	MAP/microtubule affinity-regulating kinase 3;MARK3;ortholog	protein serine/threonine kinase activity(GO:0004674);protein binding(GO:0005515);ATP binding(GO:0005524);tau protein binding(GO:0048156);tau-protein kinase activity(GO:0050321)
HUMAN|HGNC=6898|UniProtKB=P56192	MARS	Methionine--tRNA ligase, cytoplasmic;MARS;ortholog	tRNA binding(GO:0000049);aminoacyl-tRNA ligase activity(GO:0004812);methionine-tRNA ligase activity(GO:0004825);ATP binding(GO:0005524)
HUMAN|HGNC=6912|UniProtKB=P43243	MATR3	Matrin-3;MATR3;ortholog	nucleic acid binding(GO:0003676);RNA binding(GO:0003723);structural molecule activity(GO:0005198);protein binding(GO:0005515);zinc ion binding(GO:0008270);miRNA binding(GO:0035198);identical protein binding(GO:0042802)
HUMAN|HGNC=6945|UniProtKB=P25205	MCM3	DNA replication licensing factor MCM3;MCM3;ortholog	DNA binding(GO:0003677);helicase activity(GO:0004386);protein binding(GO:0005515);ATP binding(GO:0005524)
HUMAN|HGNC=6948|UniProtKB=P33992	MCM5	DNA replication licensing factor MCM5;MCM5;ortholog	DNA replication origin binding(GO:0003688);helicase activity(GO:0004386);protein binding(GO:0005515);ATP binding(GO:0005524)
HUMAN|HGNC=21163|UniProtKB=Q14676	MDC1	Mediator of DNA damage checkpoint protein 1;MDC1;ortholog	protein binding(GO:0005515);protein C-terminus binding(GO:0008022);identical protein binding(GO:0042802);FHA domain binding(GO:0070975)
HUMAN|HGNC=9234|UniProtKB=Q15648	MED1	Mediator of RNA polymerase II transcription subunit 1;MED1;ortholog	RNA polymerase II proximal promoter sequence-specific DNA binding(GO:0000978);RNA polymerase II core promoter sequence-specific DNA binding(GO:0000979);chromatin binding(GO:0003682);transcription coregulator activity(GO:0003712);transcription coactivator activity(GO:0003713);transcription corepressor activity(GO:0003714);protein binding(GO:0005515);transcription factor binding(GO:0008134);nuclear receptor binding(GO:0016922);estrogen receptor binding(GO:0030331);nuclear receptor transcription coactivator activity(GO:0030374);thyroid hormone receptor coactivator activity(GO:0030375);chromatin DNA binding(GO:0031490);nuclear hormone receptor binding(GO:0035257);mediator complex binding(GO:0036033);signaling receptor activity(GO:0038023);vitamin D receptor binding(GO:0042809);retinoic acid receptor binding(GO:0042974);peroxisome proliferator activated receptor binding(GO:0042975);thyroid hormone receptor binding(GO:0046966);LBD domain binding(GO:0050693);ubiquitin protein ligase activity(GO:0061630)
HUMAN|HGNC=2370|UniProtKB=O60244	MED14	Mediator of RNA polymerase II transcription subunit 14;MED14;ortholog	transcription coregulator activity(GO:0003712);transcription coactivator activity(GO:0003713);protein binding(GO:0005515);nuclear receptor transcription coactivator activity(GO:0030374);vitamin D receptor binding(GO:0042809)
HUMAN|HGNC=11473|UniProtKB=Q13503	MED21	Mediator of RNA polymerase II transcription subunit 21;MED21;ortholog	transcription coregulator activity(GO:0003712);transcription coactivator activity(GO:0003713);DNA-directed 5'-3' RNA polymerase activity(GO:0003899);protein binding(GO:0005515);ubiquitin protein ligase activity(GO:0061630)
HUMAN|HGNC=22963|UniProtKB=O75448	MED24	Mediator of RNA polymerase II transcription subunit 24;MED24;ortholog	transcription coregulator activity(GO:0003712);transcription coactivator activity(GO:0003713);histone acetyltransferase activity(GO:0004402);protein binding(GO:0005515);nuclear receptor transcription coactivator activity(GO:0030374);vitamin D receptor binding(GO:0042809);thyroid hormone receptor binding(GO:0046966);ubiquitin protein ligase activity(GO:0061630)
HUMAN|HGNC=20247|UniProtKB=Q7L2J0	MEPCE	7SK snRNA methylphosphate capping enzyme;MEPCE;ortholog	RNA binding(GO:0003723);O-methyltransferase activity(GO:0008171);RNA methyltransferase activity(GO:0008173);S-adenosylmethionine-dependent methyltransferase activity(GO:0008757);snRNA binding(GO:0017069)
HUMAN|HGNC=7095|UniProtKB=O15344	MID1	E3 ubiquitin-protein ligase Midline-1;MID1;ortholog	protein binding(GO:0005515);microtubule binding(GO:0008017);zinc ion binding(GO:0008270);transferase activity(GO:0016740);ubiquitin protein ligase binding(GO:0031625);identical protein binding(GO:0042802);protein homodimerization activity(GO:0042803);protein heterodimerization activity(GO:0046982);phosphoprotein binding(GO:0051219)
HUMAN|HGNC=21905|UniProtKB=Q9NXC5	MIOS	GATOR complex protein MIOS;MIOS;ortholog	protein binding(GO:0005515)
HUMAN|HGNC=7107|UniProtKB=P46013	MKI67	Proliferation marker protein Ki-67;MKI67;ortholog	DNA binding(GO:0003677);RNA binding(GO:0003723);protein binding(GO:0005515);ATP binding(GO:0005524);protein C-terminus binding(GO:0008022)
HUMAN|HGNC=18234|UniProtKB=Q96EN8	MOCOS	Molybdenum cofactor sulfurase;MOCOS;ortholog	protein binding(GO:0005515);Mo-molybdopterin cofactor sulfurase activity(GO:0008265);lyase activity(GO:0016829);molybdenum ion binding(GO:0030151);pyridoxal phosphate binding(GO:0030170);molybdenum cofactor sulfurtransferase activity(GO:0102867)
HUMAN|HGNC=7200|UniProtKB=Q9HCE1	MOV10	Putative helicase MOV-10;MOV10;ortholog	RNA binding(GO:0003723);protein binding(GO:0005515);ATP binding(GO:0005524);ATP-dependent 5'-3' RNA helicase activity(GO:0032575)
HUMAN|HGNC=7230|UniProtKB=P49959	MRE11A	Double-strand break repair protein MRE11;MRE11;ortholog	single-stranded DNA endodeoxyribonuclease activity(GO:0000014);DNA binding(GO:0003677);double-stranded DNA binding(GO:0003690);ATP-dependent DNA helicase activity(GO:0004003);nuclease activity(GO:0004518);endonuclease activity(GO:0004519);endodeoxyribonuclease activity(GO:0004520);protein binding(GO:0005515);protein C-terminus binding(GO:0008022);3'-5'-exodeoxyribonuclease activity(GO:0008296);3'-5' exonuclease activity(GO:0008408);5'-3' exonuclease activity(GO:0008409);hydrolase activity(GO:0016787);manganese ion binding(GO:0030145);identical protein binding(GO:0042802);cadherin binding(GO:0045296)
HUMAN|HGNC=18585|UniProtKB=Q96DH6	MSI2	RNA-binding protein Musashi homolog 2;MSI2;ortholog	RNA binding(GO:0003723);protein binding(GO:0005515);poly(U) RNA binding(GO:0008266)
HUMAN|HGNC=7449|UniProtKB=Q13613	MTMR1	Myotubularin-related protein 1;MTMR1;ortholog	phosphatidylinositol-3-phosphatase activity(GO:0004438);protein tyrosine phosphatase activity(GO:0004725);protein homodimerization activity(GO:0042803);phosphatidylinositol-3,5-bisphosphate 3-phosphatase activity(GO:0052629)
HUMAN|HGNC=7546|UniProtKB=Q9BQG0	MYBBP1A	Myb-binding protein 1A;MYBBP1A;ortholog	DNA binding(GO:0003677);transcription corepressor activity(GO:0003714);RNA binding(GO:0003723);transcription factor binding(GO:0008134);sequence-specific DNA binding(GO:0043565);E-box binding(GO:0070888)
HUMAN|HGNC=23386|UniProtKB=O75592	MYCBP2	E3 ubiquitin-protein ligase MYCBP2;MYCBP2;ortholog	guanyl-nucleotide exchange factor activity(GO:0005085);protein binding(GO:0005515);zinc ion binding(GO:0008270);Ran GTPase binding(GO:0008536);protein homodimerization activity(GO:0042803);ubiquitin protein ligase activity(GO:0061630)
HUMAN|HGNC=7609|UniProtKB=Q13459	MYO9B	Unconventional myosin-IXb;MYO9B;ortholog	microfilament motor activity(GO:0000146);motor activity(GO:0003774);actin binding(GO:0003779);GTPase activator activity(GO:0005096);protein binding(GO:0005515);calmodulin binding(GO:0005516);ATP binding(GO:0005524);ATPase activity(GO:0016887);Rho GTPase binding(GO:0017048);ADP binding(GO:0043531);metal ion binding(GO:0046872);Roundabout binding(GO:0048495)
HUMAN|HGNC=23246|UniProtKB=Q86TC9	MYPN	Myopalladin;MYPN;ortholog	actin binding(GO:0003779);protein binding(GO:0005515);cytoskeletal protein binding(GO:0008092);SH3 domain binding(GO:0017124);muscle alpha-actinin binding(GO:0051371);cell-cell adhesion mediator activity(GO:0098632)
HUMAN|HGNC=7652|UniProtKB=O60934	NBN	Nibrin;NBN;ortholog	damaged DNA binding(GO:0003684);ATP-dependent DNA helicase activity(GO:0004003);protein binding(GO:0005515);transcription factor binding(GO:0008134);protein N-terminus binding(GO:0047485)
HUMAN|HGNC=24305|UniProtKB=Q15021	NCAPD2	Condensin complex subunit 1;NCAPD2;ortholog	chromatin binding(GO:0003682);protein binding(GO:0005515);histone binding(GO:0042393)
HUMAN|HGNC=7658|UniProtKB=Q09161	NCBP1	Nuclear cap-binding protein subunit 1;NCBP1;ortholog	RNA cap binding(GO:0000339);RNA 7-methylguanosine cap binding(GO:0000340);RNA binding(GO:0003723);protein binding(GO:0005515)
HUMAN|HGNC=7666|UniProtKB=Q9Y2A7	NCKAP1	Nck-associated protein 1;NCKAP1;ortholog	protein binding(GO:0005515);Rac GTPase binding(GO:0048365)
HUMAN|HGNC=15909|UniProtKB=Q9HCD5	NCOA5	Nuclear receptor coactivator 5;NCOA5;ortholog	chromatin binding(GO:0003682);RNA binding(GO:0003723);protein binding(GO:0005515)
HUMAN|HGNC=7723|UniProtKB=Q8NHV4	NEDD1	Protein NEDD1;NEDD1;ortholog	protein binding(GO:0005515)
HUMAN|HGNC=12768|UniProtKB=Q9H3P2	NELFA	Negative elongation factor A;NELFA;ortholog	chromatin binding(GO:0003682);protein binding(GO:0005515)
HUMAN|HGNC=24324|UniProtKB=Q8WX92	NELFB	Negative elongation factor B;NELFB;ortholog	RNA binding(GO:0003723);protein binding(GO:0005515)
HUMAN|HGNC=15934|UniProtKB=Q8IXH7	NELFCD	Negative elongation factor C/D;NELFCD;ortholog	RNA binding(GO:0003723);protein binding(GO:0005515)
HUMAN|HGNC=13974|UniProtKB=P18615	NELFE	Negative elongation factor E;NELFE;ortholog	chromatin binding(GO:0003682);RNA binding(GO:0003723);protein binding(GO:0005515)
HUMAN|HGNC=7765|UniProtKB=P21359	NF1	Neurofibromin;NF1;ortholog	GTPase activator activity(GO:0005096);protein binding(GO:0005515);phosphatidylethanolamine binding(GO:0008429);phosphatidylcholine binding(GO:0031210)
[illegible]	[illegible]	[illegible]	[illegible]

HUMAN|HGNC=5187|UniProtKB=Q99873	PRMT1	Protein arginine N-methyltransferase 1;PRMT1;ortholog	RNA binding(GO:0003723);protein binding(GO:0005515);methyltransferase activity(GO:0008168);N-methyltransferase activity(GO:0008170);protein methyltransferase activity(GO:0008276);methyl-CpG binding(GO:0008327);histone-arginine N-methyltransferase activity(GO:0008469);protein-arginine N-methyltransferase activity(GO:0016274);enzyme binding(GO:0019899);protein-arginine omega-N monomethyltransferase activity(GO:0035241);protein-arginine omega-N asymmetric methyltransferase activity(GO:0035242);histone methyltransferase activity(GO:0042054);identical protein binding(GO:0042802);histone methyltransferase activity (H4-R3 specific)(GO:0044020);mitogen-activated protein kinase p38 binding(GO:0048273);S-adenosyl-L-methionine binding(GO:1904047)
HUMAN|HGNC=10894|UniProtKB=O14744	PRMT5	Protein arginine N-methyltransferase 5;PRMT5;ortholog	transcription corepressor activity(GO:0003714);protein binding(GO:0005515);methyltransferase activity(GO:0008168);methyl-CpG binding(GO:0008327);histone-arginine N-methyltransferase activity(GO:0008469);protein-arginine N-methyltransferase activity(GO:0016274);protein-arginine omega-N symmetric methyltransferase activity(GO:0035243);identical protein binding(GO:0042802);ribonucleoprotein complex binding(GO:0043021);histone methyltransferase activity (H4-R3 specific)(GO:0044020);protein heterodimerization activity(GO:0046982);E-box binding(GO:0070888)
HUMAN|HGNC=17896|UniProtKB=Q9UMS4	PRPF19	Pre-mRNA-processing factor 19;PRPF19;ortholog	ubiquitin-protein transferase activity(GO:0004842);protein binding(GO:0005515);ubiquitin-ubiquitin ligase activity(GO:0034450);identical protein binding(GO:0042802);ubiquitin protein ligase activity(GO:0061630)
HUMAN|HGNC=17348|UniProtKB=O43395	PRPF3	U4/U6 small nuclear ribonucleoprotein Prp3;PRPF3;ortholog	RNA binding(GO:0003723);protein binding(GO:0005515);identical protein binding(GO:0042802)
HUMAN|HGNC=15446|UniProtKB=Q8WWY3	PRPF31	U4/U6 small nuclear ribonucleoprotein Prp31;PRPF31;ortholog	RNA binding(GO:0003723);protein binding(GO:0005515);U4 snRNA binding(GO:0030621);U4atac snRNA binding(GO:0030622);ribonucleoprotein complex binding(GO:0043021);snRNP binding(GO:0070990)
HUMAN|HGNC=17349|UniProtKB=O43172	PRPF4	U4/U6 small nuclear ribonucleoprotein Prp4;PRPF4;ortholog	protein binding(GO:0005515);U6 snRNA binding(GO:0017070);U4 snRNA binding(GO:0030621)
HUMAN|HGNC=16463|UniProtKB=O75400	PRPF40A	Pre-mRNA-processing factor 40 homolog A;PRPF40A;ortholog	RNA binding(GO:0003723);protein binding(GO:0005515)
HUMAN|HGNC=15860|UniProtKB=O94906	PRPF6	Pre-mRNA-processing factor 6;PRPF6;ortholog	transcription coactivator activity(GO:0003713);RNA binding(GO:0003723);protein binding(GO:0005515);ribonucleoprotein complex binding(GO:0043021);androgen receptor binding(GO:0050681)
HUMAN|HGNC=17340|UniProtKB=Q6P2Q9	PRPF8	Pre-mRNA-processing-splicing factor 8;PRPF8;ortholog	second spliceosomal transesterification activity(GO:0000386);RNA binding(GO:0003723);protein binding(GO:0005515);U6 snRNA binding(GO:0017070);U1 snRNA binding(GO:0030619);U2 snRNA binding(GO:0030620);U5 snRNA binding(GO:0030623);K63-linked polyubiquitin modification-dependent protein binding(GO:0070530);pre-mRNA intronic binding(GO:0097157)
HUMAN|HGNC=9462|UniProtKB=P60891	PRPS1	Ribose-phosphate pyrophosphokinase 1;PRPS1;ortholog	magnesium ion binding(GO:0000287);ribose phosphate diphosphokinase activity(GO:0004749);protein binding(GO:0005515);ATP binding(GO:0005524);kinase activity(GO:0016301);identical protein binding(GO:0042802);protein homodimerization activity(GO:0042803)
HUMAN|HGNC=13918|UniProtKB=P48634	PRRC2A	Protein PRRC2A;PRRC2A;ortholog	RNA binding(GO:0003723);protein binding(GO:0005515)
HUMAN|HGNC=24903|UniProtKB=Q9Y520	PRRC2C	Protein PRRC2C;PRRC2C;ortholog	RNA binding(GO:0003723);protein C-terminus binding(GO:0008022)
HUMAN|HGNC=9658|UniProtKB=P29350	PTPN6	Tyrosine-protein phosphatase non-receptor type 6;PTPN6;ortholog	phosphotyrosine residue binding(GO:0001784);protein tyrosine phosphatase activity(GO:0004725);transmembrane receptor protein tyrosine phosphatase activity(GO:0005001);protein binding(GO:0005515);SH3 domain binding(GO:0017124);protein kinase binding(GO:0019901);SH2 domain binding(GO:0042169);cell adhesion molecule binding(GO:0050839);phosphorylation-dependent protein binding(GO:0140031)
HUMAN|HGNC=14957|UniProtKB=Q14671	PUM1	Pumilio homolog 1;PUM1;ortholog	RNA binding(GO:0003723);mRNA 3'-UTR binding(GO:0003730);protein binding(GO:0005515);miRNA binding(GO:0035198)
HUMAN|HGNC=9701|UniProtKB=Q00577	PURA	Transcriptional activator protein Pur-alpha;PURA;ortholog	translation repressor activity, mRNA regulatory element binding(GO:0000900);RNA polymerase II regulatory region sequence-specific DNA binding(GO:0000977);DNA-binding transcription factor activity, RNA polymerase II-specific(GO:0000981);DNA-binding transcription repressor activity, RNA polymerase II-specific(GO:0001227);double-stranded DNA binding(GO:0003690);double-stranded telomeric DNA binding(GO:0003691);single-stranded DNA binding(GO:0003697);RNA binding(GO:0003723);protein binding(GO:0005515);transcription factor binding(GO:0008134);purine-rich negative regulatory element binding(GO:0032422);sequence-specific DNA binding(GO:0043565);SMAD binding(GO:0046332)
HUMAN|HGNC=9702|UniProtKB=Q96QR8	PURB	Transcriptional activator protein Pur-beta;PURB;ortholog	RNA polymerase II regulatory region sequence-specific DNA binding(GO:0000977);DNA-binding transcription factor activity, RNA polymerase II-specific(GO:0000981);DNA-binding transcription repressor activity, RNA polymerase II-specific(GO:0001227);double-stranded DNA binding(GO:0003690);single-stranded DNA binding(GO:0003697);RNA binding(GO:0003723);mRNA binding(GO:0003729);transcription factor binding(GO:0008134);purine-rich negative regulatory element binding(GO:0032422);sequence-specific DNA binding(GO:0043565);SMAD binding(GO:0046332)
HUMAN|HGNC=9751|UniProtKB=P47897	QARS	Glutamine--tRNA ligase;QARS;ortholog	aminoacyl-tRNA ligase activity(GO:0004812);glutamine-tRNA ligase activity(GO:0004819);protein kinase inhibitor activity(GO:0004860);protein binding(GO:0005515);ATP binding(GO:0005524);protein kinase binding(GO:0019901)
HUMAN|HGNC=24845|UniProtKB=Q9BXF6	RAB11FIP5	Rab11 family-interacting protein 5;RAB11FIP5;ortholog	protein binding(GO:0005515);Rab GTPase binding(GO:0017137);gamma-tubulin binding(GO:0043015)
HUMAN|HGNC=9804|UniProtKB=Q9H0H5	RACGAP1	Rac GTPase-activating protein 1;RACGAP1;ortholog	GTPase activator activity(GO:0005096);protein binding(GO:0005515);phosphatidylinositol-3,4,5-trisphosphate binding(GO:0005547);microtubule binding(GO:0008017);protein kinase binding(GO:0019901);alpha-tubulin binding(GO:0043014);gamma-tubulin binding(GO:0043015);metal ion binding(GO:0046872);beta-tubulin binding(GO:0048487)
HUMAN|HGNC=9816|UniProtKB=Q92878	RAD50	DNA repair protein RAD50;RAD50;ortholog	single-stranded DNA endodeoxyribonuclease activity(GO:0000014);DNA binding(GO:0003677);double-stranded telomeric DNA binding(GO:0003691);ATP-dependent DNA helicase activity(GO:0004003);adenylate kinase activity(GO:0004017);protein binding(GO:0005515);ATP binding(GO:0005524);3'-5' exonuclease activity(GO:0008408);ATPase activity(GO:0016887);protein binding, bridging(GO:0030674);single-stranded telomeric DNA binding(GO:0043047);metal ion binding(GO:0046872);G-quadruplex DNA binding(GO:0051880)
HUMAN|HGNC=17770|UniProtKB=Q6GYQ0	RALGAPA1	Ral GTPase-activating protein subunit alpha-1;RALGAPA1;ortholog	GTPase activator activity(GO:0005096);protein heterodimerization activity(GO:0046982)
HUMAN|HGNC=29221|UniProtKB=Q86X10	RALGAPB	Ral GTPase-activating protein subunit beta;RALGAPB;ortholog	GTPase activator activity(GO:0005096);protein heterodimerization activity(GO:0046982)
HUMAN|HGNC=9846|UniProtKB=P62826	RAN	GTP-binding nuclear protein Ran;RAN;ortholog	magnesium ion binding(GO:0000287);chromatin binding(GO:0003682);RNA binding(GO:0003723);GTPase activity(GO:0003924);nuclear export signal receptor activity(GO:0005049);protein binding(GO:0005515);GTP binding(GO:0005525);GDP binding(GO:0019003);cadherin binding(GO:0045296);protein heterodimerization activity(GO:0046982);androgen receptor binding(GO:0050681);pre-miRNA binding(GO:0070883)
HUMAN|HGNC=9848|UniProtKB=P49792	RANBP2	E3 SUMO-protein ligase RanBP2;RANBP2;ortholog	RNA binding(GO:0003723);GTPase activator activity(GO:0005096);protein binding(GO:0005515);Ran GTPase binding(GO:0008536);SUMO transferase activity(GO:0019789);protein-containing complex binding(GO:0044877);metal ion binding(GO:0046872)
HUMAN|HGNC=9870|UniProtKB=P54136	RARS	Arginine--tRNA ligase, cytoplasmic;RARS;ortholog	tRNA binding(GO:0000049);arginine-tRNA ligase activity(GO:0004814);protein binding(GO:0005515);ATP binding(GO:0005524);arginine binding(GO:0034618);cadherin binding(GO:0045296)
HUMAN|HGNC=30296|UniProtKB=Q8IY67	RAVER1	Ribonucleoprotein PTB-binding 1;RAVER1;ortholog	nucleic acid binding(GO:0003676);RNA binding(GO:0003723);protein binding(GO:0005515)
HUMAN|HGNC=9906|UniProtKB=O43251	RBFOX2	RNA binding protein fox-1 homolog 2;RBFOX2;ortholog	transcription corepressor activity(GO:0003714);RNA binding(GO:0003723);mRNA binding(GO:0003729);protein binding(GO:0005515);transcription factor binding(GO:0008134)
HUMAN|HGNC=9896|UniProtKB=P98175	RBM10	RNA-binding protein 10;RBM10;ortholog	RNA binding(GO:0003723);protein binding(GO:0005515);miRNA binding(GO:0035198);identical protein binding(GO:0042802);metal ion binding(GO:0046872)
HUMAN|HGNC=32310|UniProtKB=Q8IXT5	RBM12B	RNA-binding protein 12B;RBM12B;ortholog	nucleic acid binding(GO:0003676);RNA binding(GO:0003723);protein binding(GO:0005515)
HUMAN|HGNC=14959|UniProtKB=Q96T37	RBM15	Putative RNA-binding protein 15;RBM15;ortholog	RNA binding(GO:0003723);mRNA binding(GO:0003729);protein binding(GO:0005515)
HUMAN|HGNC=23244|UniProtKB=P49756	RBM25	RNA-binding protein 25;RBM25;ortholog	nucleic acid binding(GO:0003676);RNA binding(GO:0003723);mRNA binding(GO:0003729);protein binding(GO:0005515)
HUMAN|HGNC=29243|UniProtKB=Q9P2N5	RBM27	RNA-binding protein 27;RBM27;ortholog	RNA binding(GO:0003723);mRNA binding(GO:0003729);metal ion binding(GO:0046872)
HUMAN|HGNC=27223|UniProtKB=Q96EV2	RBM33	RNA-binding protein 33;RBM33;ortholog	nucleic acid binding(GO:0003676);RNA binding(GO:0003723)
HUMAN|HGNC=30358|UniProtKB=A0AV96	RBM47	RNA-binding protein 47;RBM47;ortholog	RNA binding(GO:0003723);mRNA binding(GO:0003729)
HUMAN|HGNC=9902|UniProtKB=P52756	RBM5	RNA-binding protein 5;RBM5;ortholog	nucleic acid binding(GO:0003676);DNA binding(GO:0003677);RNA binding(GO:0003723);mRNA binding(GO:0003729);protein binding(GO:0005515);metal ion binding(GO:0046872)
HUMAN|HGNC=28611|UniProtKB=Q6R327	RICTOR	Rapamycin-insensitive companion of mTOR;RICTOR;ortholog	protein binding(GO:0005515);enzyme activator activity(GO:0008047);protein kinase binding(GO:0019901);ribosome binding(GO:0043022)
HUMAN|HGNC=23207|UniProtKB=Q5UIP0	RIF1	Telomere-associated protein RIF1;RIF1;ortholog	protein binding(GO:0005515)
HUMAN|HGNC=10018|UniProtKB=Q06587	RING1	E3 ubiquitin-protein ligase RING1;RING1;ortholog	chromatin binding(GO:0003682);protein binding(GO:0005515);transferase activity(GO:0016740);metal ion binding(GO:0046872);ubiquitin-protein transferase activator activity(GO:0097027)
HUMAN|HGNC=18518|UniProtKB=O75792	RNASEH2A	Ribonuclease H2 subunit A;RNASEH2A;ortholog	RNA binding(GO:0003723);RNA-DNA hybrid ribonuclease activity(GO:0004523);ribonuclease activity(GO:0004540);metal ion binding(GO:0046872)
HUMAN|HGNC=25671|UniProtKB=Q5TBB1	RNASEH2B	Ribonuclease H2 subunit B;RNASEH2B;ortholog	RNA-DNA hybrid ribonuclease activity(GO:0004523)
HUMAN|HGNC=24116|UniProtKB=Q8TDP1	RNASEH2C	Ribonuclease H2 subunit C;RNASEH2C;ortholog	
HUMAN|HGNC=10062|UniProtKB=Q5VTR2	RNF20	E3 ubiquitin-protein ligase BRE1A;RNF20;ortholog	p53 binding(GO:0002039);chromatin binding(GO:0003682);transcription coactivator activity(GO:0003713);mRNA 3'-UTR binding(GO:0003730);ubiquitin-protein transferase activity(GO:0004842);protein binding(GO:0005515);ubiquitin protein ligase binding(GO:0031625);histone binding(GO:0042393);metal ion binding(GO:0046872)
HUMAN|HGNC=16867|UniProtKB=O75150	RNF40	E3 ubiquitin-protein ligase BRE1B;RNF40;ortholog	mRNA 3'-UTR binding(GO:0003730);ubiquitin-protein transferase activity(GO:0004842);protein binding(GO:0005515);syntaxin-1 binding(GO:0017075);ubiquitin conjugating enzyme binding(GO:0031624);ubiquitin protein ligase binding(GO:0031625);protein homodimerization activity(GO:0042803);protein-containing complex binding(GO:0044877);metal ion binding(GO:0046872)
HUMAN|HGNC=10075|UniProtKB=O43148	RNMT	mRNA cap guanine-N7 methyltransferase;RNMT;ortholog	RNA binding(GO:0003723);mRNA (guanine-N7-)-methyltransferase activity(GO:0004482);protein binding(GO:0005515)
HUMAN|HGNC=10080|UniProtKB=Q15287	RNPS1	RNA-binding protein with serine-rich domain 1;RNPS1;ortholog	RNA binding(GO:0003723);mRNA 3'-UTR binding(GO:0003730);protein binding(GO:0005515)
HUMAN|HGNC=10311|UniProtKB=Q02543	RPL18A	60S ribosomal protein L18a;RPL18A;ortholog	RNA binding(GO:0003723);structural constituent of ribosome(GO:0003735);protein binding(GO:0005515)
HUMAN|HGNC=17688|UniProtKB=P78346	RPP30	Ribonuclease P protein subunit p30;RPP30;ortholog	RNA binding(GO:0003723);ribonuclease P activity(GO:0004526);ribonuclease activity(GO:0004540);protein binding(GO:0005515)
HUMAN|HGNC=10445|UniProtKB=Q92600	RQCD1	CCR4-NOT transcription complex subunit 9;CNOT9;ortholog	epidermal growth factor receptor binding(GO:0005154);protein binding(GO:0005515);kinase binding(GO:0019900);protein domain specific binding(GO:0019904);protein homodimerization activity(GO:0042803)
HUMAN|HGNC=24534|UniProtKB=O76021	RSL1D1	Ribosomal L1 domain-containing protein 1;RSL1D1;ortholog	RNA binding(GO:0003723);mRNA 3'-UTR binding(GO:0003730);cadherin binding(GO:0045296);mRNA 5'-UTR binding(GO:0048027)
HUMAN|HGNC=26935|UniProtKB=Q9Y3I0	RTCB	tRNA-splicing ligase RtcB homolog;RTCB;ortholog	RNA binding(GO:0003723);RNA ligase (ATP) activity(GO:0003972);protein binding(GO:0005515);ATP binding(GO:0005524);vinculin binding(GO:0017166);metal ion binding(GO:0046872)
HUMAN|HGNC=23169|UniProtKB=Q9Y224	RTRAF	RNA transcription, translation and transport factor protein;RTRAF;ortholog	RNA polymerase II complex binding(GO:0000993);RNA binding(GO:0003723);protein binding(GO:0005515);identical protein binding(GO:0042802)
HUMAN|HGNC=10474|UniProtKB=Q9Y265	RUVBL1	RuvB-like 1;RUVBL1;ortholog	TFIID-class transcription factor complex binding(GO:0001094);DNA helicase activity(GO:0003678);transcription coactivator activity(GO:0003713);ATP-dependent DNA helicase activity(GO:0004003);protein binding(GO:0005515);ATP binding(GO:0005524);ATPase activity(GO:0016887);TBP-class protein binding(GO:0017025);ATP-dependent 5'-3' DNA helicase activity(GO:0043141);ADP binding(GO:0043531);cadherin binding(GO:0045296);ATPase binding(GO:0051117)
HUMAN|HGNC=10538|UniProtKB=O43290	SART1	U4/U6.U5 tri-snRNP-associated protein 1;SART1;ortholog	RNA binding(GO:0003723);protein binding(GO:0005515)
HUMAN|HGNC=10542|UniProtKB=O95248	SBF1	Myotubularin-related protein 5;SBF1;ortholog	protein tyrosine/serine/threonine phosphatase activity(GO:0008138);Rab guanyl-nucleotide exchange factor activity(GO:0017112);phosphatase regulator activity(GO:0019208)
HUMAN|HGNC=30377|UniProtKB=Q14160	SCRIB	Protein scribble homolog;SCRIB;ortholog	protein binding(GO:0005515);ionotropic glutamate receptor binding(GO:0035255);cadherin binding(GO:0045296)
HUMAN|HGNC=10697|UniProtKB=P55735	SEC13	Protein SEC13 homolog;SEC13;ortholog	structural molecule activity(GO:0005198);protein binding(GO:0005515);identical protein binding(GO:0042802)
HUMAN|HGNC=29006|UniProtKB=O15027	SEC16A	Protein transport protein Sec16A;SEC16A;ortholog	protein binding(GO:0005515)
HUMAN|HGNC=30379|UniProtKB=Q96EE3	SEH1L	Nucleoporin SEH1;SEH1L;ortholog	structural molecule activity(GO:0005198);protein binding(GO:0005515)
HUMAN|HGNC=14349|UniProtKB=Q9P0V9	SEPT10	Septin-10;SEPT10;ortholog	GTPase activity(GO:0003924);protein binding(GO:0005515);GTP binding(GO:0005525);protein-containing complex scaffold activity(GO:0032947)
HUMAN|HGNC=7729|UniProtKB=Q15019	SEPT2	Septin-2;SEPT2;ortholog	GTPase activity(GO:0003924);protein binding(GO:0005515);GTP binding(GO:0005525);protein-containing complex scaffold activity(GO:0032947);identical protein binding(GO:0042802);cadherin binding(GO:0045296)
HUMAN|HGNC=15848|UniProtKB=Q14141	SEPT6	Septin-6;SEPT6;ortholog	GTPase activity(GO:0003924);protein binding(GO:0005515);GTP binding(GO:0005525);protein-containing complex scaffold activity(GO:0032947)
HUMAN|HGNC=1717|UniProtKB=Q16181	SEPT7	Septin-7;SEPT7;ortholog	GTPase activity(GO:0003924);structural molecule activity(GO:0005198);protein binding(GO:0005515);GTP binding(GO:0005525);protein-containing complex scaffold activity(GO:0032947);identical protein binding(GO:0042802);cadherin binding(GO:0045296)
HUMAN|HGNC=16511|UniProtKB=Q92599	SEPT8	Septin-8;SEPT8;ortholog	GTPase activity(GO:0003924);protein binding(GO:0005515);GTP binding(GO:0005525);protein-containing complex scaffold activity(GO:0032947)
HUMAN|HGNC=7323|UniProtKB=Q9UHD8	SEPT9	Septin-9;SEPT9;ortholog	GTPase activity(GO:0003924);protein binding(GO:0005515);GTP binding(GO:0005525);protein-containing complex scaffold activity(GO:0032947);cadherin binding(GO:0045296)
HUMAN|HGNC=17860|UniProtKB=Q8NC51	SERBP1	Plasminogen activator inhibitor 1 RNA-binding protein;SERBP1;ortholog	RNA binding(GO:0003723);mRNA 3'-UTR binding(GO:0003730);protein binding(GO:0005515);SUMO binding(GO:0032183);cadherin binding(GO:0045296)
HUMAN|HGNC=12950|UniProtKB=Q15637	SF1	Splicing factor 1;SF1;ortholog	transcription corepressor activity(GO:0003714);RNA binding(GO:0003723);protein binding(GO:0005515);zinc ion binding(GO:0008270);identical protein binding(GO:0042802);pre-mRNA branch point binding(GO:0045131)
HUMAN|HGNC=10765|UniProtKB=Q15459	SF3A1	Splicing factor 3A subunit 1;SF3A1;ortholog	RNA binding(GO:0003723);protein binding(GO:0005515)
HUMAN|HGNC=10766|UniProtKB=Q15428	SF3A2	Splicing factor 3A subunit 2;SF3A2;ortholog	nucleic acid binding(GO:0003676);RNA binding(GO:0003723);protein binding(GO:0005515);zinc ion binding(GO:0008270)
HUMAN|HGNC=10767|UniProtKB=Q12874	SF3A3	Splicing factor 3A subunit 3;SF3A3;ortholog	RNA binding(GO:0003723);protein binding(GO:0005515);zinc ion binding(GO:0008270)
HUMAN|HGNC=10768|UniProtKB=O75533	SF3B1	Splicing factor 3B subunit 1;SF3B1;ortholog	RNA binding(GO:0003723);mRNA binding(GO:0003729);protein binding(GO:0005515)
HUMAN|HGNC=10769|UniProtKB=Q13435	SF3B2	Splicing factor 3B subunit 2;SF3B2;ortholog	RNA binding(GO:0003723);protein binding(GO:0005515)
HUMAN|HGNC=10770|UniProtKB=Q15393	SF3B3	Splicing factor 3B subunit 3;SF3B3;ortholog	nucleic acid binding(GO:0003676);protein binding(GO:0005515);protein-containing complex binding(GO:0044877)
HUMAN|HGNC=30096|UniProtKB=Q9Y3B4	SF3B6	Splicing factor 3B subunit 6;SF3B6;ortholog	RNA binding(GO:0003723);mRNA binding(GO:0003729);protein binding(GO:0005515)
HUMAN|HGNC=29547|UniProtKB=Q8NEM2	SHCBP1	SHC SH2 domain-binding protein 1;SHCBP1;ortholog	protein binding(GO:0005515);SH2 domain binding(GO:0042169)
HUMAN|HGNC=10852|UniProtKB=P34897	SHMT2	Serine hydroxymethyltransferase, mitochondrial;SHMT2;ortholog	chromatin binding(GO:0003682);glycine hydroxymethyltransferase activity(GO:0004372);protein binding(GO:0005515);zinc ion binding(GO:0008270);L-allo-threonine aldolase activity(GO:0008732);amino acid binding(GO:0016597);pyridoxal phosphate binding(GO:0030170);identical protein binding(GO:0042802);cobalt ion binding(GO:0050897);serine binding(GO:0070905)
HUMAN|HGNC=11100|UniProtKB=P51532	SMARCA4	Transcription activator BRG1;SMARCA4;ortholog	RNA polymerase II proximal promoter sequence-specific DNA binding(GO:0000978);RNA polymerase II distal enhancer sequence-specific DNA binding(GO:0000980);RNA polymerase I CORE element sequence-specific DNA binding(GO:0001164);p53 binding(GO:0002039);transcription coactivator activity(GO:0003713);transcription corepressor activity(GO:0003714);RNA binding(GO:0003723);helicase activity(GO:0004386);protein binding(GO:0005515);ATP binding(GO:0005524);DNA-dependent ATPase activity(GO:0008094);transcription factor binding(GO:0008134);hydrolase activity, acting on acid anhydrides(GO:0016817);ATPase activity(GO:0016887);Tat protein binding(GO:0030957);nucleosomal DNA binding(GO:0031492);histone binding(GO:0042393);protein N-terminus binding(GO:0047485);androgen receptor binding(GO:0050681);DNA polymerase binding(GO:0070182);lysine-acetylated histone binding(GO:0070577)
HUMAN|HGNC=11103|UniProtKB=Q12824	SMARCB1	SWI/SNF-related matrix-associated actin-dependent regulator of chromatin subfamily B member 1;SMARCB1;ortholog	RNA polymerase II proximal promoter sequence-specific DNA binding(GO:0000978);RNA polymerase II distal enhancer sequence-specific DNA binding(GO:0000980);RNA polymerase I CORE element sequence-specific DNA binding(GO:0001164);p53 binding(GO:0002039);DNA binding(GO:0003677);transcription coactivator activity(GO:0003713);protein binding(GO:0005515);Tat protein binding(GO:0030957);nucleosomal DNA binding(GO:0031492)
HUMAN|HGNC=11104|UniProtKB=Q92922	SMARCC1	SWI/SNF complex subunit SMARCC1;SMARCC1;ortholog	RNA polymerase II proximal promoter sequence-specific DNA binding(GO:0000978);RNA polymerase II distal enhancer sequence-specific DNA binding(GO:0000980);DNA-binding transcription factor activity, RNA polymerase II-specific(GO:0000981);transcription coactivator activity(GO:0003713);protein binding(GO:0005515);nucleosomal DNA binding(GO:0031492);protein N-terminus binding(GO:0047485)
HUMAN|HGNC=11105|UniProtKB=Q8TAQ2	SMARCC2	SWI/SNF complex subunit SMARCC2;SMARCC2;ortholog	RNA polymerase II proximal promoter sequence-specific DNA binding(GO:0000978);RNA polymerase II distal enhancer sequence-specific DNA binding(GO:0000980);DNA-binding transcription factor activity, RNA polymerase II-specific(GO:0000981);DNA binding(GO:0003677);chromatin binding(GO:0003682);transcription coactivator activity(GO:0003713);protein binding(GO:0005515);nucleosomal DNA binding(GO:0031492)
HUMAN|HGNC=18247|UniProtKB=Q2TAY7	SMU1	WD40 repeat-containing protein SMU1;SMU1;ortholog	protein binding(GO:0005515)
HUMAN|HGNC=30859|UniProtKB=O75643	SNRNP200	U5 small nuclear ribonucleoprotein 200 kDa helicase;SNRNP200;ortholog	RNA binding(GO:0003723);ATP-dependent RNA helicase activity(GO:0004004);helicase activity(GO:0004386);protein binding(GO:0005515);ATP binding(GO:0005524);ATP-dependent helicase activity(GO:0008026);identical protein binding(GO:0042802)
HUMAN|HGNC=30857|UniProtKB=Q96DI7	SNRNP40	U5 small nuclear ribonucleoprotein 40 kDa protein;SNRNP40;ortholog	RNA binding(GO:0003723);protein binding(GO:0005515)
HUMAN|HGNC=11150|UniProtKB=P08621	SNRNP70	U1 small nuclear ribonucleoprotein 70 kDa;SNRNP70;ortholog	RNA binding(GO:0003723);mRNA binding(GO:0003729);protein binding(GO:0005515);U1 snRNA binding(GO:0030619)
HUMAN|HGNC=11151|UniProtKB=P09012	SNRPA	U1 small nuclear ribonucleoprotein A;SNRPA;ortholog	RNA binding(GO:0003723);protein binding(GO:0005515);U1 snRNA binding(GO:0030619);snRNA stem-loop binding(GO:0035614);identical protein binding(GO:0042802);U1 snRNP binding(GO:1990446)
HUMAN|HGNC=11152|UniProtKB=P09661	SNRPA1	U2 small nuclear ribonucleoprotein A';SNRPA1;ortholog	RNA binding(GO:0003723);protein binding(GO:0005515);U2 snRNA binding(GO:0030620)
HUMAN|HGNC=11157|UniProtKB=P09234	SNRPC	U1 small nuclear ribonucleoprotein C;SNRPC;ortholog	RNA binding(GO:0003723);single-stranded RNA binding(GO:0003727);mRNA binding(GO:0003729);protein binding(GO:0005515);zinc ion binding(GO:0008270);U1 snRNA binding(GO:0030619);pre-mRNA 5'-splice site binding(GO:0030627);protein homodimerization activity(GO:0042803);U1 snRNP binding(GO:1990446)
HUMAN|HGNC=11158|UniProtKB=P62314	SNRPD1	Small nuclear ribonucleoprotein Sm D1;SNRPD1;ortholog	RNA binding(GO:0003723);protein binding(GO:0005515);U1 snRNP binding(GO:1990446)
HUMAN|HGNC=11159|UniProtKB=P62316	SNRPD2;SNRPD1	Small nuclear ribonucleoprotein Sm D2;SNRPD2;ortholog	RNA binding(GO:0003723);protein binding(GO:0005515)
HUMAN|HGNC=11160|UniProtKB=P62318	SNRPD3	Small nuclear ribonucleoprotein Sm D3;SNRPD3;ortholog	RNA binding(GO:0003723);protein binding(GO:0005515);enzyme binding(GO:0019899);telomerase RNA binding(GO:0070034);histone pre-mRNA DCP binding(GO:0071208);U7 snRNA binding(GO:0071209);U1 snRNP binding(GO:1990446)
HUMAN|HGNC=11161|UniProtKB=P62304	SNRPE	Small nuclear ribonucleoprotein E;SNRPE;ortholog	RNA binding(GO:0003723);protein binding(GO:0005515)
HUMAN|HGNC=11164|UniProtKB=P63162	SNRPN	Small nuclear ribonucleoprotein-associated protein N;SNRPN;ortholog	RNA binding(GO:0003723);protein binding(GO:0005515)
HUMAN|HGNC=20073|UniProtKB=Q96L92	SNX27	Sorting nexin-27;SNX27;ortholog	protein binding(GO:0005515);phosphatidylinositol-3-phosphate binding(GO:0032266);phosphatidylinositol binding(GO:0035091)
HUMAN|HGNC=11183|UniProtKB=P18583	SON	Protein SON;SON;ortholog	DNA binding(GO:0003677);RNA binding(GO:0003723);protein binding(GO:0005515);RS domain binding(GO:0050733)
HUMAN|HGNC=19751|UniProtKB=O75044	SRGAP2	SLIT-ROBO Rho GTPase-activating protein 2;SRGAP2;ortholog	GTPase activator activity(GO:0005096);protein binding(GO:0005515);identical protein binding(GO:0042802);protein homodimerization activity(GO:0042803);Rac GTPase binding(GO:0048365)
HUMAN|HGNC=11303|UniProtKB=O76094	SRP72	Signal recognition particle subunit SRP72;SRP72;ortholog	RNA binding(GO:0003723);signal recognition particle binding(GO:0005047);protein binding(GO:0005515);7S RNA binding(GO:0008312);TPR domain binding(GO:0030911);ribosome binding(GO:0043022)
HUMAN|HGNC=16638|UniProtKB=Q8IYB3	SRRM1	Serine/arginine repetitive matrix protein 1;SRRM1;ortholog	DNA binding(GO:0003677);RNA binding(GO:0003723);protein binding(GO:0005515)
HUMAN|HGNC=16639|UniProtKB=Q9UQ35	SRRM2	Serine/arginine repetitive matrix protein 2;SRRM2;ortholog	RNA binding(GO:0003723);protein N-terminus binding(GO:0047485);C2H2 zinc finger domain binding(GO:0070742)
HUMAN|HGNC=24101|UniProtKB=Q9BXP5	SRRT	Serrate RNA effector molecule homolog;SRRT;ortholog	nucleic acid binding(GO:0003676);DNA binding(GO:0003677);RNA binding(GO:0003723);protein binding(GO:0005515)
HUMAN|HGNC=10780|UniProtKB=Q07955	SRSF1	Serine/arginine-rich splicing factor 1;SRSF1;ortholog	RNA binding(GO:0003723);mRNA binding(GO:0003729);protein binding(GO:0005515);protein kinase B binding(GO:0043422);DNA topoisomerase binding(GO:0044547);RS domain binding(GO:0050733)
HUMAN|HGNC=16713|UniProtKB=O75494	SRSF10	Serine/arginine-rich splicing factor 10;SRSF10;ortholog	RNA binding(GO:0003723);protein binding(GO:0005515);RS domain binding(GO:0050733);unfolded protein binding(GO:0051082)
HUMAN|HGNC=10782|UniProtKB=Q05519	SRSF11	Serine/arginine-rich splicing factor 11;SRSF11;ortholog	RNA binding(GO:0003723);protein binding(GO:0005515)
HUMAN|HGNC=10783|UniProtKB=Q01130	SRSF2	Serine/arginine-rich splicing factor 2;SRSF2;ortholog	transcription corepressor activity(GO:0003714);RNA binding(GO:0003723);protein binding(GO:0005515)
HUMAN|HGNC=10788|UniProtKB=Q13247	SRSF6	Serine/arginine-rich splicing factor 6;SRSF6;ortholog	RNA binding(GO:0003723);protein binding(GO:0005515);pre-mRNA binding(GO:0036002)
HUMAN|HGNC=10789|UniProtKB=Q16629	SRSF7	Serine/arginine-rich splicing factor 7;SRSF7;ortholog	nucleic acid binding(GO:0003676);RNA binding(GO:0003723);protein binding(GO:0005515);zinc ion binding(GO:0008270);protein domain specific binding(GO:0019904)
HUMAN|HGNC=10791|UniProtKB=Q13242	SRSF9	Serine/arginine-rich splicing factor 9;SRSF9;ortholog	RNA binding(GO:0003723);protein binding(GO:0005515);protein domain specific binding(GO:0019904)
HUMAN|HGNC=25824|UniProtKB=Q6ZW31	SYDE1	Rho GTPase-activating protein SYDE1;SYDE1;ortholog	GTPase activator activity(GO:0005096)
HUMAN|HGNC=22935|UniProtKB=Q92797	SYMPK	Symplekin;SYMPK;ortholog	protein binding(GO:0005515)
HUMAN|HGNC=16918|UniProtKB=O60506	SYNCRIP	Heterogeneous nuclear ribonucleoprotein Q;SYNCRIP;ortholog	RNA binding(GO:0003723);mRNA binding(GO:0003729);protein binding(GO:0005515);poly(A) binding(GO:0008143);mRNA 5'-UTR binding(GO:0048027)
HUMAN|HGNC=29364|UniProtKB=Q9C0D5	TANC1	Protein TANC1;TANC1;ortholog	protein binding(GO:0005515)
HUMAN|HGNC=19165|UniProtKB=O60343	TBC1D4	TBC1 domain family member 4;TBC1D4;ortholog	GTPase activator activity(GO:0005096);Rab GTPase binding(GO:0017137)
HUMAN|HGNC=29529|UniProtKB=Q9BZK7	TBL1XR1	F-box-like/WD repeat-containing protein TBL1XR1;TBL1XR1;ortholog	transcription corepressor activity(GO:0003714);protein binding(GO:0005515);beta-catenin binding(GO:0008013);histone binding(GO:0042393);transcription regulatory region DNA binding(GO:0044212);protein N-terminus binding(GO:0047485)
HUMAN|HGNC=17165|UniProtKB=Q9UBB9	TFIP11	Tuftelin-interacting protein 11;TFIP11;ortholog	nucleic acid binding(GO:0003676);protein binding(GO:0005515)
HUMAN|HGNC=11827|UniProtKB=Q07157	TJP1	Tight junction protein ZO-1;TJP1;ortholog	protein binding(GO:0005515);calmodulin binding(GO:0005516);protein C-terminus binding(GO:0008022);protein domain specific binding(GO:0019904);cadherin binding(GO:0045296);connexin binding(GO:0071253)
HUMAN|HGNC=11828|UniProtKB=Q9UDY2	TJP2	Tight junction protein ZO-2;TJP2;ortholog	guanylate kinase activity(GO:0004385);protein binding(GO:0005515);ferric iron binding(GO:0008199);protein domain specific binding(GO:0019904);protein binding, bridging(GO:0030674);cadherin binding(GO:0045296)
HUMAN|HGNC=19081|UniProtKB=Q9C0C2	TNKS1BP1	182 kDa tankyrase-1-binding protein;TNKS1BP1;ortholog	protein binding(GO:0005515);enzyme binding(GO:0019899);protein-containing complex binding(GO:0044877);cadherin binding(GO:0045296);ankyrin repeat binding(GO:0071532)
HUMAN|HGNC=17008|UniProtKB=Q92547	TOPBP1	DNA topoisomerase 2-binding protein 1;TOPBP1;ortholog	DNA binding(GO:0003677);protein binding(GO:0005515);protein C-terminus binding(GO:0008022);identical protein binding(GO:0042802)
HUMAN|HGNC=10781|UniProtKB=P62995	TRA2B	Transformer-2 protein homolog beta;TRA2B;ortholog	RNA binding(GO:0003723);mRNA binding(GO:0003729);protein binding(GO:0005515);protein domain specific binding(GO:0019904);pre-mRNA binding(GO:0036002);identical protein binding(GO:0042802)
HUMAN|HGNC=20456|UniProtKB=Q6Q0C0	TRAF7	E3 ubiquitin-protein ligase TRAF7;TRAF7;ortholog	ubiquitin-protein transferase activity(GO:0004842);protein binding(GO:0005515);zinc ion binding(GO:0008270)
HUMAN|HGNC=17009|UniProtKB=Q9H2D6	TRIOBP	TRIO and F-actin-binding protein;TRIOBP;ortholog	GTP-Rho binding(GO:0017049);ubiquitin protein ligase binding(GO:0031625);myosin II binding(GO:0045159);actin filament binding(GO:0051015)
HUMAN|HGNC=12310|UniProtKB=Q15650	TRIP4	Activating signal cointegrator 1;TRIP4;ortholog	protease binding(GO:0002020);transcription coactivator activity(GO:0003713);protein binding(GO:0005515);zinc ion binding(GO:0008270);nuclear receptor binding(GO:0016922);protein kinase binding(GO:0019901);estrogen receptor binding(GO:0030331);histone acetyltransferase binding(GO:0035035);ubiquitin-like protein ligase binding(GO:0044389)
HUMAN|HGNC=20316|UniProtKB=Q86TN4	TRPT1	tRNA 2'-phosphotransferase 1;TRPT1;ortholog	tRNA 2'-phosphotransferase activity(GO:0000215);molecular_function(GO:0003674);transferase activity, transferring phosphorus-containing groups(GO:0016772)
HUMAN|HGNC=12347|UniProtKB=Q9Y4A5	TRRAP	Transformation/transcription domain-associated protein;TRRAP;ortholog	transcription coregulator activity(GO:0003712);protein binding(GO:0005515);kinase activity(GO:0016301)
HUMAN|HGNC=12407|UniProtKB=P68366	TUBA4A	Tubulin alpha-4A chain;TUBA4A;ortholog	GTPase activity(GO:0003924);structural constituent of cytoskeleton(GO:0005200);protein binding(GO:0005515);GTP binding(GO:0005525);protein kinase binding(GO:0019901)
HUMAN|HGNC=20776|UniProtKB=Q9BUF5	TUBB6	Tubulin beta-6 chain;TUBB6;ortholog	molecular_function(GO:0003674);GTPase activity(GO:0003924);structural constituent of cytoskeleton(GO:0005200);GTP binding(GO:0005525)
HUMAN|HGNC=20773|UniProtKB=Q3ZCM7	TUBB8	Tubulin beta-8 chain;TUBB8;ortholog	molecular_function(GO:0003674);GTPase activity(GO:0003924);structural constituent of cytoskeleton(GO:0005200);GTP binding(GO:0005525)
HUMAN|HGNC=12417|UniProtKB=P23258	TUBG1	Tubulin gamma-1 chain;TUBG1;ortholog	GTPase activity(GO:0003924);structural constituent of cytoskeleton(GO:0005200);protein binding(GO:0005515);GTP binding(GO:0005525);identical protein binding(GO:0042802)
HUMAN|HGNC=18599|UniProtKB=Q9BSJ2	TUBGCP2	Gamma-tubulin complex component 2;TUBGCP2;ortholog	protein binding(GO:0005515);gamma-tubulin binding(GO:0043015);microtubule minus-end binding(GO:0051011)
HUMAN|HGNC=18598|UniProtKB=Q96CW5	TUBGCP3	Gamma-tubulin complex component 3;TUBGCP3;ortholog	structural molecule activity(GO:0005198);structural constituent of cytoskeleton(GO:0005200);protein binding(GO:0005515);gamma-tubulin binding(GO:0043015);microtubule minus-end binding(GO:0051011)
HUMAN|HGNC=30855|UniProtKB=O15042	U2SURP	U2 snRNP-associated SURP motif-containing protein;U2SURP;ortholog	RNA binding(GO:0003723);protein binding(GO:0005515)
HUMAN|HGNC=16806|UniProtKB=O95071	UBR5	E3 ubiquitin-protein ligase UBR5;UBR5;ortholog	RNA binding(GO:0003723);ubiquitin-protein transferase activity(GO:0004842);protein binding(GO:0005515);zinc ion binding(GO:0008270);ubiquitin-ubiquitin ligase activity(GO:0034450);ubiquitin binding(GO:0043130);ubiquitin protein ligase activity(GO:0061630)
HUMAN|HGNC=17854|UniProtKB=Q9HAU5	UPF2	Regulator of nonsense transcripts 2;UPF2;ortholog	RNA binding(GO:0003723);protein binding(GO:0005515);telomeric DNA binding(GO:0042162)
HUMAN|HGNC=12608|UniProtKB=Q14694	USP10	Ubiquitin carboxyl-terminal hydrolase 10;USP10;ortholog	p53 binding(GO:0002039);RNA binding(GO:0003723);cysteine-type endopeptidase activity(GO:0004197);thiol-dependent ubiquitin-specific protease activity(GO:0004843);protein binding(GO:0005515);thiol-dependent ubiquitinyl hydrolase activity(GO:0036459);ion channel binding(GO:0044325)
HUMAN|HGNC=20071|UniProtKB=Q53GS9	USP39	U4/U6.U5 tri-snRNP-associated protein 2;USP39;ortholog	zinc ion binding(GO:0008270);thiol-dependent ubiquitinyl hydrolase activity(GO:0036459)
HUMAN|HGNC=12630|UniProtKB=Q93009	USP7	Ubiquitin carboxyl-terminal hydrolase 7;USP7;ortholog	p53 binding(GO:0002039);cysteine-type endopeptidase activity(GO:0004197);thiol-dependent ubiquitin-specific protease activity(GO:0004843);protein binding(GO:0005515);protein C-terminus binding(GO:0008022);transcription factor binding(GO:0008134);ubiquitin protein ligase binding(GO:0031625);thiol-dependent ubiquitinyl hydrolase activity(GO:0036459);ubiquitinyl hydrolase activity(GO:0101005);Lys48-specific deubiquitinase activity(GO:1990380)
HUMAN|HGNC=12692|UniProtKB=P08670	VIM	Vimentin;VIM;ortholog	double-stranded RNA binding(GO:0003725);structural molecule activity(GO:0005198);structural constituent of cytoskeleton(GO:0005200);structural constituent of eye lens(GO:0005212);protein binding(GO:0005515);protein C-terminus binding(GO:0008022);protein domain specific binding(GO:0019904);identical protein binding(GO:0042802);scaffold protein binding(GO:0097110);keratin filament binding(GO:1990254)
HUMAN|HGNC=24500|UniProtKB=Q69YN4	virma	Protein virilizer homolog;VIRMA;ortholog	RNA binding(GO:0003723);protein binding(GO:0005515)
HUMAN|HGNC=16461|UniProtKB=Q9Y2W2	WBP11	WW domain-binding protein 11;WBP11;ortholog	single-stranded DNA binding(GO:0003697);RNA binding(GO:0003723);protein binding(GO:0005515);WW domain binding(GO:0050699)
HUMAN|HGNC=16405|UniProtKB=O43709	WBSCR22	Probable 18S rRNA (guanine-N(7))-methyltransferase;BUD23;ortholog	RNA binding(GO:0003723);methyltransferase activity(GO:0008168);rRNA (guanine) methyltransferase activity(GO:0016435);protein heterodimerization activity(GO:0046982)
HUMAN|HGNC=20852|UniProtKB=Q96S15	WDR24	GATOR complex protein WDR24;WDR24;ortholog	protein binding(GO:0005515)
HUMAN|HGNC=25651|UniProtKB=Q9C0J8	WDR33	pre-mRNA 3' end processing protein WDR33;WDR33;ortholog	RNA binding(GO:0003723)
HUMAN|HGNC=12757|UniProtKB=P61964	WDR5	WD repeat-containing protein 5;WDR5;ortholog	protein binding(GO:0005515);methylated histone binding(GO:0035064);histone binding(GO:0042393);histone methyltransferase activity (H3-K4 specific)(GO:0042800);histone acetyltransferase activity (H4-K5 specific)(GO:0043995);histone acetyltransferase activity (H4-K8 specific)(GO:0043996);histone acetyltransferase activity (H4-K16 specific)(GO:0046972)
HUMAN|HGNC=30300|UniProtKB=Q9GZS3	WDR61	WD repeat-containing protein 61;WDR61;ortholog	protein binding(GO:0005515)
HUMAN|HGNC=29652|UniProtKB=Q9BQA1	WDR77	Methylosome protein 50;WDR77;ortholog	protein binding(GO:0005515);methyl-CpG binding(GO:0008327);nuclear receptor transcription coactivator activity(GO:0030374)
HUMAN|HGNC=28826|UniProtKB=Q6UXN9	WDR82	WD repeat-containing protein 82;WDR82;ortholog	chromatin binding(GO:0003682);protein binding(GO:0005515);histone methyltransferase activity (H3-K4 specific)(GO:0042800)
HUMAN|HGNC=14540|UniProtKB=Q9H4A3	WNK1	Serine/threonine-protein kinase WNK1;WNK1;ortholog	magnesium ion binding(GO:0000287);protein kinase activity(GO:0004672);protein serine/threonine kinase activity(GO:0004674);protein binding(GO:0005515);ATP binding(GO:0005524);chloride channel inhibitor activity(GO:0019869);potassium channel inhibitor activity(GO:0019870);protein kinase binding(GO:0019901);phosphatase binding(GO:0019902);protein serine/threonine kinase inhibitor activity(GO:0030291);protein kinase activator activity(GO:0030295)
HUMAN|HGNC=16846|UniProtKB=Q15007	WTAP	Pre-mRNA-splicing regulator WTAP;WTAP;ortholog	protein binding(GO:0005515)
HUMAN|HGNC=14089|UniProtKB=Q9HCS7	XAB2	Pre-mRNA-splicing factor SYF1;XAB2;ortholog	protein binding(GO:0005515)
HUMAN|HGNC=25489|UniProtKB=Q9ULM3	YEATS2	YEATS domain-containing protein 2;YEATS2;ortholog	DNA-binding transcription factor activity, RNA polymerase II-specific(GO:0000981);protein binding(GO:0005515);TBP-class protein binding(GO:0017025);histone binding(GO:0042393);modification-dependent protein binding(GO:0140030)
HUMAN|HGNC=17798|UniProtKB=P49750	YLPM1	YLP motif-containing protein 1;YLPM1;ortholog	RNA binding(GO:0003723);protein binding(GO:0005515)
HUMAN|HGNC=31675|UniProtKB=Q9Y5A9	YTHDF2	YTH domain-containing family protein 2;YTHDF2;ortholog	RNA binding(GO:0003723);protein binding(GO:0005515);N6-methyladenosine-containing RNA binding(GO:1990247)
HUMAN|HGNC=12856|UniProtKB=P25490	YY1	Transcriptional repressor protein YY1;YY1;ortholog	four-way junction DNA binding(GO:0000400);RNA polymerase II proximal promoter sequence-specific DNA binding(GO:0000978);RNA polymerase II distal enhancer sequence-specific DNA binding(GO:0000980);DNA-binding transcription factor activity, RNA polymerase II-specific(GO:0000981);proximal promoter sequence-specific DNA binding(GO:0000987);enhancer sequence-specific DNA binding(GO:0001158);DNA-binding transcription repressor activity, RNA polymerase II-specific(GO:0001227);nucleic acid binding(GO:0003676);DNA binding(GO:0003677);DNA-binding transcription factor activity(GO:0003700);transcription coactivator activity(GO:0003713);transcription corepressor activity(GO:0003714);RNA binding(GO:0003723);protein binding(GO:0005515);zinc ion binding(GO:0008270);sequence-specific DNA binding(GO:0043565);transcription regulatory region DNA binding(GO:0044212);SMAD binding(GO:0046332)
HUMAN|HGNC=29093|UniProtKB=O75152	ZC3H11A	Zinc finger CCCH domain-containing protein 11A;ZC3H11A;ortholog	DNA-binding transcription factor activity, RNA polymerase II-specific(GO:0000981);RNA binding(GO:0003723);mRNA binding(GO:0003729);protein binding(GO:0005515);metal ion binding(GO:0046872)
HUMAN|HGNC=20368|UniProtKB=Q5T200	ZC3H13	Zinc finger CCCH domain-containing protein 13;ZC3H13;ortholog	RNA binding(GO:0003723);protein binding(GO:0005515);metal ion binding(GO:0046872)
HUMAN|HGNC=17277|UniProtKB=Q96KR1	ZFR	Zinc finger RNA-binding protein;ZFR;ortholog	DNA binding(GO:0003677);RNA binding(GO:0003723);double-stranded RNA binding(GO:0003725);single-stranded RNA binding(GO:0003727);protein binding(GO:0005515);zinc ion binding(GO:0008270)
HUMAN|HGNC=28079|UniProtKB=Q86UK7	ZNF598	E3 ubiquitin-protein ligase ZNF598;ZNF598;ortholog	nucleic acid binding(GO:0003676);RNA binding(GO:0003723);protein binding(GO:0005515);ribosome binding(GO:0043022);metal ion binding(GO:0046872);ubiquitin protein ligase activity(GO:0061630)

Gene ID	Mapped IDs	Gene Name	Go database MF Complete
HUMAN|HGNC=17066|UniProtKB=Q9UKV3	ACIN1	Apoptotic chromatin condensation inducer in the nucleus;ACIN1;ortholog	nucleic acid binding(GO:0003676);RNA binding(GO:0003723);protein binding(GO:0005515);ATPase activity(GO:0016887);enzyme binding(GO:0019899)
HUMAN|HGNC=24124|UniProtKB=O96019	ACTL6A	Actin-like protein 6A;ACTL6A;ortholog	RNA polymerase II proximal promoter sequence-specific DNA binding(GO:0000978);RNA polymerase II distal enhancer sequence-specific DNA binding(GO:0000980);chromatin binding(GO:0003682);transcription coactivator activity(GO:0003713);protein binding(GO:0005515);nucleosomal DNA binding(GO:0031492)
HUMAN|HGNC=225|UniProtKB=P55265	ADAR	Double-stranded RNA-specific adenosine deaminase;ADAR;ortholog	DNA binding(GO:0003677);RNA binding(GO:0003723);double-stranded RNA binding(GO:0003725);double-stranded RNA adenosine deaminase activity(GO:0003726);adenosine deaminase activity(GO:0004000);protein binding(GO:0005515);tRNA-specific adenosine deaminase activity(GO:0008251);metal ion binding(GO:0046872)
HUMAN|HGNC=10648|UniProtKB=Q12904	AIMP1	Aminoacyl tRNA synthase complex-interacting multifunctional protein 1;AIMP1;ortholog	tRNA binding(GO:0000049);cytokine activity(GO:0005125);protein binding(GO:0005515);protein homodimerization activity(GO:0042803);GTPase binding(GO:0051020)
HUMAN|HGNC=25996|UniProtKB=Q6P6C2	ALKBH5	RNA demethylase ALKBH5;ALKBH5;ortholog	RNA binding(GO:0003723);2-oxoglutarate-dependent dioxygenase activity(GO:0016706);metal ion binding(GO:0046872);RNA N6-methyladenosine dioxygenase activity(GO:1990931)
HUMAN|HGNC=24714|UniProtKB=Q8IWZ3	ANKHD1	Ankyrin repeat and KH domain-containing protein 1;ANKHD1;ortholog	RNA binding(GO:0003723);protein binding(GO:0005515)
HUMAN|HGNC=594|UniProtKB=Q9BZZ5	API5	Apoptosis inhibitor 5;API5;ortholog	RNA binding(GO:0003723);protein binding(GO:0005515);fibroblast growth factor binding(GO:0017134)
HUMAN|HGNC=10555|UniProtKB=Q99700	ATXN2	Ataxin-2;ATXN2;ortholog	RNA binding(GO:0003723);epidermal growth factor receptor binding(GO:0005154);protein binding(GO:0005515);protein C-terminus binding(GO:0008022)
HUMAN|HGNC=31326|UniProtKB=Q8WWM7	ATXN2L	Ataxin-2-like protein;ATXN2L;ortholog	RNA binding(GO:0003723);protein binding(GO:0005515);cadherin binding(GO:0045296)
HUMAN|HGNC=30237|UniProtKB=Q6P1N0	CC2D1A	Coiled-coil and C2 domain-containing protein 1A;CC2D1A;ortholog	RNA polymerase II proximal promoter sequence-specific DNA binding(GO:0000978);DNA-binding transcription repressor activity, RNA polymerase II-specific(GO:0001227);protein binding(GO:0005515);cadherin binding(GO:0045296)
HUMAN|HGNC=24236|UniProtKB=Q8IX12	CCAR1	Cell division cycle and apoptosis regulator protein 1;CCAR1;ortholog	RNA polymerase II distal enhancer sequence-specific DNA binding(GO:0000980);transcription coactivator activity(GO:0003713);transcription corepressor activity(GO:0003714);RNA binding(GO:0003723);protein binding(GO:0005515);nuclear receptor transcription coactivator activity(GO:0030374)
HUMAN|HGNC=23360|UniProtKB=Q8N163	CCAR2	Cell cycle and apoptosis regulator protein 2;CCAR2;ortholog	RNA polymerase II complex binding(GO:0000993);RNA binding(GO:0003723);enzyme inhibitor activity(GO:0004857);protein binding(GO:0005515);enzyme binding(GO:0019899);nuclear receptor transcription coactivator activity(GO:0030374)
HUMAN|HGNC=1743|UniProtKB=Q99459	CDC5L	Cell division cycle 5-like protein;CDC5L;ortholog	RNA polymerase II regulatory region sequence-specific DNA binding(GO:0000977);DNA-binding transcription factor activity, RNA polymerase II-specific(GO:0000981);transcription corepressor binding(GO:0001222);DNA-binding transcription activator activity, RNA polymerase II-specific(GO:0001228);DNA binding(GO:0003677);RNA binding(GO:0003723);protein binding(GO:0005515);protein phosphatase 1 binding(GO:0008157);protein kinase binding(GO:0019901);leucine zipper domain binding(GO:0043522);WD40-repeat domain binding(GO:0071987)
HUMAN|HGNC=24511|UniProtKB=Q9Y3Y2	CHTOP	Chromatin target of PRMT1 protein;CHTOP;ortholog	RNA binding(GO:0003723);protein binding(GO:0005515);methyl-CpG binding(GO:0008327)
HUMAN|HGNC=13164|UniProtKB=P62633	CNBP	Cellular nucleic acid-binding protein;CNBP;ortholog	RNA polymerase II regulatory region sequence-specific DNA binding(GO:0000977);DNA-binding transcription repressor activity, RNA polymerase II-specific(GO:0001227);nucleic acid binding(GO:0003676);DNA-binding transcription factor activity(GO:0003700);RNA binding(GO:0003723);protein binding(GO:0005515);zinc ion binding(GO:0008270)
HUMAN|HGNC=7877|UniProtKB=A5YKK6	CNOT1	CCR4-NOT transcription complex subunit 1;CNOT1;ortholog	RNA binding(GO:0003723);poly(A)-specific ribonuclease activity(GO:0004535);protein binding(GO:0005515);protein domain specific binding(GO:0019904);estrogen receptor binding(GO:0030331);protein-containing complex scaffold activity(GO:0032947);retinoic acid receptor binding(GO:0042974);armadillo repeat domain binding(GO:0070016)
HUMAN|HGNC=14101|UniProtKB=Q9UIV1	CNOT7	CCR4-NOT transcription complex subunit 7;CNOT7;ortholog	3'-5'-exoribonuclease activity(GO:0000175);nucleic acid binding(GO:0003676);transcription corepressor activity(GO:0003714);RNA binding(GO:0003723);exoribonuclease activity(GO:0004532);poly(A)-specific ribonuclease activity(GO:0004535);protein binding(GO:0005515);transcription factor binding(GO:0008134);metal ion binding(GO:0046872)
HUMAN|HGNC=2324|UniProtKB=Q10570	CPSF1	Cleavage and polyadenylation specificity factor subunit 1;CPSF1;ortholog	nucleic acid binding(GO:0003676);protein binding(GO:0005515);enzyme binding(GO:0019899);mRNA 3'-UTR AU-rich region binding(GO:0035925)
HUMAN|HGNC=2325|UniProtKB=Q9P2I0	CPSF2	Cleavage and polyadenylation specificity factor subunit 2;CPSF2;ortholog	RNA binding(GO:0003723);protein binding(GO:0005515)
HUMAN|HGNC=2326|UniProtKB=Q9UKF6	CPSF3	Cleavage and polyadenylation specificity factor subunit 3;CPSF3;ortholog	RNA binding(GO:0003723);endoribonuclease activity(GO:0004521);protein binding(GO:0005515);5'-3' exonuclease activity(GO:0008409);metal ion binding(GO:0046872)
HUMAN|HGNC=2327|UniProtKB=O95639	CPSF4	Cleavage and polyadenylation specificity factor subunit 4;CPSF4;ortholog	nucleic acid binding(GO:0003676);RNA binding(GO:0003723);protein binding(GO:0005515);zinc ion binding(GO:0008270);metal ion binding(GO:0046872)
HUMAN|HGNC=15762|UniProtKB=Q9BZJ0	CRNKL1	Crooked neck-like protein 1;CRNKL1;ortholog	RNA binding(GO:0003723);protein binding(GO:0005515)
HUMAN|HGNC=2483|UniProtKB=Q05048	CSTF1	Cleavage stimulation factor subunit 1;CSTF1;ortholog	RNA binding(GO:0003723);protein binding(GO:0005515)
HUMAN|HGNC=2485|UniProtKB=Q12996	CSTF3	Cleavage stimulation factor subunit 3;CSTF3;ortholog	RNA binding(GO:0003723);mRNA binding(GO:0003729);protein binding(GO:0005515)
HUMAN|HGNC=29322|UniProtKB=Q9HCG8	CWC22	Pre-mRNA-splicing factor CWC22 homolog;CWC22;ortholog	RNA binding(GO:0003723);protein binding(GO:0005515)
HUMAN|HGNC=2678|UniProtKB=P14868	DARS	Aspartate--tRNA ligase, cytoplasmic;DARS;ortholog	nucleic acid binding(GO:0003676);RNA binding(GO:0003723);aminoacylase activity(GO:0004046);aspartate-tRNA ligase activity(GO:0004815);protein binding(GO:0005515);ATP binding(GO:0005524)
HUMAN|HGNC=15594|UniProtKB=Q9UK59	DBR1	Lariat debranching enzyme;DBR1;ortholog	RNA binding(GO:0003723);RNA lariat debranching enzyme activity(GO:0008419);hydrolase activity(GO:0016787);metal ion binding(GO:0046872)
HUMAN|HGNC=2734|UniProtKB=Q92499	DDX1	ATP-dependent RNA helicase DDX1;DDX1;ortholog	nucleic acid binding(GO:0003676);DNA binding(GO:0003677);chromatin binding(GO:0003682);transcription coregulator activity(GO:0003712);RNA binding(GO:0003723);RNA helicase activity(GO:0003724);double-stranded RNA binding(GO:0003725);helicase activity(GO:0004386);nuclease activity(GO:0004518);exonuclease activity(GO:0004527);protein binding(GO:0005515);ATP binding(GO:0005524);ATP-dependent helicase activity(GO:0008026);poly(A) binding(GO:0008143);DNA/RNA helicase activity(GO:0033677)
HUMAN|HGNC=2743|UniProtKB=Q9UHI6	DDX20	Probable ATP-dependent RNA helicase DDX20;DDX20;ortholog	DNA binding(GO:0003677);ATP-dependent RNA helicase activity(GO:0004004);protein binding(GO:0005515);ATP binding(GO:0005524);protein domain specific binding(GO:0019904);protein binding, bridging(GO:0030674);histone deacetylase binding(GO:0042826);repressing transcription factor binding(GO:0070491)
HUMAN|HGNC=17347|UniProtKB=Q9BUQ8	DDX23	Probable ATP-dependent RNA helicase DDX23;DDX23;ortholog	nucleic acid binding(GO:0003676);RNA binding(GO:0003723);ATP-dependent RNA helicase activity(GO:0004004);protein binding(GO:0005515);ATP binding(GO:0005524)
HUMAN|HGNC=2745|UniProtKB=O00571	DDX3X	ATP-dependent RNA helicase DDX3X;DDX3X;ortholog	nucleic acid binding(GO:0003676);DNA binding(GO:0003677);RNA binding(GO:0003723);GTPase activity(GO:0003924);ATP-dependent DNA helicase activity(GO:0004003);ATP-dependent RNA helicase activity(GO:0004004);helicase activity(GO:0004386);protein binding(GO:0005515);ATP binding(GO:0005524);transcription factor binding(GO:0008134);poly(A) binding(GO:0008143);eukaryotic initiation factor 4E binding(GO:0008190);ATPase activity(GO:0016887);nucleoside-triphosphatase activity(GO:0017111);translation initiation factor binding(GO:0031369);RNA strand annealing activity(GO:0033592);RNA stem-loop binding(GO:0035613);ribosomal small subunit binding(GO:0043024);CTPase activity(GO:0043273);protein serine/threonine kinase activator activity(GO:0043539);cadherin binding(GO:0045296);mRNA 5'-UTR binding(GO:0048027)
HUMAN|HGNC=18674|UniProtKB=Q9UJV9	DDX41	Probable ATP-dependent RNA helicase DDX41;DDX41;ortholog	nucleic acid binding(GO:0003676);DNA binding(GO:0003677);RNA binding(GO:0003723);helicase activity(GO:0004386);protein binding(GO:0005515);ATP binding(GO:0005524);metal ion binding(GO:0046872)
HUMAN|HGNC=18676|UniProtKB=Q86XP3	DDX42	ATP-dependent RNA helicase DDX42;DDX42;ortholog	nucleic acid binding(GO:0003676);RNA binding(GO:0003723);helicase activity(GO:0004386);protein binding(GO:0005515);ATP binding(GO:0005524)
HUMAN|HGNC=20038|UniProtKB=Q9Y2R4	DDX52	Probable ATP-dependent RNA helicase DDX52;DDX52;ortholog	RNA binding(GO:0003723);helicase activity(GO:0004386);ATP binding(GO:0005524)
HUMAN|HGNC=2747|UniProtKB=P26196	DDX6	Probable ATP-dependent RNA helicase DDX6;DDX6;ortholog	nucleic acid binding(GO:0003676);RNA binding(GO:0003723);RNA helicase activity(GO:0003724);helicase activity(GO:0004386);protein binding(GO:0005515);ATP binding(GO:0005524);protein domain specific binding(GO:0019904);cadherin binding(GO:0045296)
HUMAN|HGNC=2739|UniProtKB=O60231	DHX16	Pre-mRNA-splicing factor ATP-dependent RNA helicase DHX16;DHX16;ortholog	nucleic acid binding(GO:0003676);RNA binding(GO:0003723);RNA helicase activity(GO:0003724);helicase activity(GO:0004386);protein binding(GO:0005515);ATP binding(GO:0005524);ATPase activity(GO:0016887);ATP-dependent 3'-5' RNA helicase activity(GO:0034459)
HUMAN|HGNC=15815|UniProtKB=Q7Z478	DHX29	ATP-dependent RNA helicase DHX29;DHX29;ortholog	RNA binding(GO:0003723);translation initiation factor activity(GO:0003743);ATP-dependent RNA helicase activity(GO:0004004);ATP binding(GO:0005524);ATP-dependent 3'-5' RNA helicase activity(GO:0034459);ribosomal small subunit binding(GO:0043024);cadherin binding(GO:0045296)
HUMAN|HGNC=16716|UniProtKB=Q7L2E3	DHX30	Putative ATP-dependent RNA helicase DHX30;DHX30;ortholog	G-quadruplex RNA binding(GO:0002151);nucleic acid binding(GO:0003676);chromatin binding(GO:0003682);RNA binding(GO:0003723);double-stranded RNA binding(GO:0003725);ATP-dependent DNA helicase activity(GO:0004003);ATP-dependent RNA helicase activity(GO:0004004);helicase activity(GO:0004386);protein binding(GO:0005515);ATP binding(GO:0005524);ATP-dependent 3'-5' RNA helicase activity(GO:0034459)
HUMAN|HGNC=14410|UniProtKB=Q9H2U1	DHX36	ATP-dependent RNA helicase DHX36;DHX36;ortholog	magnesium ion binding(GO:0000287);RNA polymerase II proximal promoter sequence-specific DNA binding(GO:0000978);G-quadruplex RNA binding(GO:0002151);nucleic acid binding(GO:0003676);single-stranded DNA binding(GO:0003697);RNA binding(GO:0003723);double-stranded RNA binding(GO:0003725);mRNA 3'-UTR binding(GO:0003730);ATP-dependent DNA helicase activity(GO:0004003);ATP-dependent RNA helicase activity(GO:0004004);helicase activity(GO:0004386);protein binding(GO:0005515);ATP binding(GO:0005524);DNA-dependent ATPase activity(GO:0008094);ATP-dependent 3'-5' RNA helicase activity(GO:0034459);mRNA 3'-UTR AU-rich region binding(GO:0035925);histone deacetylase binding(GO:0042826);transcription regulatory region DNA binding(GO:0044212);mRNA 5'-UTR binding(GO:0048027);G-quadruplex DNA binding(GO:0051880);telomerase RNA binding(GO:0070034);pre-miRNA binding(GO:0070883)
HUMAN|HGNC=17211|UniProtKB=Q92620	DHX38	Pre-mRNA-splicing factor ATP-dependent RNA helicase PRP16;DHX38;ortholog	RNA binding(GO:0003723);protein binding(GO:0005515);ATP binding(GO:0005524);ATP-dependent 3'-5' RNA helicase activity(GO:0034459)
HUMAN|HGNC=18018|UniProtKB=Q8IX18	DHX40	Probable ATP-dependent RNA helicase DHX40;DHX40;ortholog	nucleic acid binding(GO:0003676);RNA binding(GO:0003723);helicase activity(GO:0004386);ATP binding(GO:0005524);ATP-dependent 3'-5' RNA helicase activity(GO:0034459)
HUMAN|HGNC=2976|UniProtKB=P26358	DNMT1	DNA (cytosine-5)-methyltransferase 1;DNMT1;ortholog	DNA-binding transcription factor activity, RNA polymerase II-specific(GO:0000981);DNA binding(GO:0003677);RNA binding(GO:0003723);DNA (cytosine-5-)-methyltransferase activity(GO:0003886);protein binding(GO:0005515);methyltransferase activity(GO:0008168);zinc ion binding(GO:0008270);methyl-CpG binding(GO:0008327);DNA-methyltransferase activity(GO:0009008);promoter-specific chromatin binding(GO:1990841)
HUMAN|HGNC=16160|UniProtKB=Q9H147	DNTTIP1	Deoxynucleotidyltransferase terminal-interacting protein 1;DNTTIP1;ortholog	DNA binding(GO:0003677);protein binding(GO:0005515);nucleosome binding(GO:0031491);protein homodimerization activity(GO:0042803)
HUMAN|HGNC=2961|UniProtKB=Q14204	DYNC1H1	Cytoplasmic dynein 1 heavy chain 1;DYNC1H1;ortholog	RNA binding(GO:0003723);microtubule motor activity(GO:0003777);protein binding(GO:0005515);ATP binding(GO:0005524);ATP-dependent microtubule motor activity, minus-end-directed(GO:0008569);dynein light chain binding(GO:0045503);dynein intermediate chain binding(GO:0045505);dynein light intermediate chain binding(GO:0051959)
HUMAN|HGNC=18745|UniProtKB=Q9Y6G9	DYNC1LI1	Cytoplasmic dynein 1 light intermediate chain 1;DYNC1LI1;ortholog	RNA binding(GO:0003723);microtubule motor activity(GO:0003777);protein binding(GO:0005515);ATP binding(GO:0005524);GDP binding(GO:0019003);dynein heavy chain binding(GO:0045504)
HUMAN|HGNC=30858|UniProtKB=Q15029	EFTUD2	116 kDa U5 small nuclear ribonucleoprotein component;EFTUD2;ortholog	RNA binding(GO:0003723);GTPase activity(GO:0003924);protein binding(GO:0005515);GTP binding(GO:0005525);U5 snRNA binding(GO:0030623)
HUMAN|HGNC=3266|UniProtKB=P20042	EIF2S2	Eukaryotic translation initiation factor 2 subunit 2;EIF2S2;ortholog	RNA binding(GO:0003723);translation initiation factor activity(GO:0003743);protein binding(GO:0005515);translation factor activity, RNA binding(GO:0008135);metal ion binding(GO:0046872)
HUMAN|HGNC=3293|UniProtKB=O60573	EIF4E2	Eukaryotic translation initiation factor 4E type 2;EIF4E2;ortholog	RNA cap binding(GO:0000339);RNA 7-methylguanosine cap binding(GO:0000340);RNA binding(GO:0003723);translation initiation factor activity(GO:0003743);protein binding(GO:0005515);translation factor activity, RNA binding(GO:0008135);ubiquitin protein ligase binding(GO:0031625)
HUMAN|HGNC=3296|UniProtKB=Q04637	EIF4G1	Eukaryotic translation initiation factor 4 gamma 1;EIF4G1;ortholog	RNA binding(GO:0003723);mRNA binding(GO:0003729);translation initiation factor activity(GO:0003743);protein binding(GO:0005515);ATP binding(GO:0005524);translation factor activity, RNA binding(GO:0008135);eukaryotic initiation factor 4E binding(GO:0008190);translation initiation factor binding(GO:0031369);protein-containing complex scaffold activity(GO:0032947);identical protein binding(GO:0042802)
HUMAN|HGNC=3508|UniProtKB=Q01844	EWSR1	RNA-binding protein EWS;EWSR1;ortholog	RNA binding(GO:0003723);protein binding(GO:0005515);calmodulin binding(GO:0005516);identical protein binding(GO:0042802);metal ion binding(GO:0046872)
HUMAN|HGNC=17097|UniProtKB=Q13868	EXOSC2	Exosome complex component RRP4;EXOSC2;ortholog	3'-5'-exoribonuclease activity(GO:0000175);RNA binding(GO:0003723);protein binding(GO:0005515);7S RNA binding(GO:0008312)
HUMAN|HGNC=13247|UniProtKB=Q9NZB2	FAM120A	Constitutive coactivator of PPAR-gamma-like protein 1;FAM120A;ortholog	RNA binding(GO:0003723)
HUMAN|HGNC=24520|UniProtKB=Q8NCA5	FAM98A	Protein FAM98A;FAM98A;ortholog	RNA binding(GO:0003723);protein binding(GO:0005515);protein methyltransferase activity(GO:0008276)
HUMAN|HGNC=26773|UniProtKB=Q52LJ0	FAM98B	Protein FAM98B;FAM98B;ortholog	RNA binding(GO:0003723);protein binding(GO:0005515);protein methyltransferase activity(GO:0008276);identical protein binding(GO:0042802)
HUMAN|HGNC=4004|UniProtKB=Q96AE4	FUBP1	Far upstream element-binding protein 1;FUBP1;ortholog	single-stranded DNA binding(GO:0003697);DNA-binding transcription factor activity(GO:0003700);RNA binding(GO:0003723);protein binding(GO:0005515)
HUMAN|HGNC=4005|UniProtKB=Q96I24	FUBP3	Far upstream element-binding protein 3;FUBP3;ortholog	RNA polymerase II proximal promoter sequence-specific DNA binding(GO:0000978);DNA-binding transcription activator activity, RNA polymerase II-specific(GO:0001228);RNA binding(GO:0003723);protein binding(GO:0005515)
HUMAN|HGNC=4010|UniProtKB=P35637	FUS	RNA-binding protein FUS;FUS;ortholog	DNA binding(GO:0003677);chromatin binding(GO:0003682);transcription coactivator activity(GO:0003713);RNA binding(GO:0003723);protein binding(GO:0005515);estrogen receptor binding(GO:0030331);myosin V binding(GO:0031489);ionotropic glutamate receptor binding(GO:0035255);identical protein binding(GO:0042802);metal ion binding(GO:0046872);retinoid X receptor binding(GO:0046965);thyroid hormone receptor binding(GO:0046966)
HUMAN|HGNC=30292|UniProtKB=Q13283	G3BP1	Ras GTPase-activating protein-binding protein 1;G3BP1;ortholog	DNA binding(GO:0003677);RNA binding(GO:0003723);mRNA binding(GO:0003729);ATP-dependent DNA helicase activity(GO:0004003);ATP-dependent RNA helicase activity(GO:0004004);endonuclease activity(GO:0004519);protein binding(GO:0005515);ATP binding(GO:0005524)
HUMAN|HGNC=30291|UniProtKB=Q9UN86	G3BP2	Ras GTPase-activating protein-binding protein 2;G3BP2;ortholog	RNA binding(GO:0003723);mRNA binding(GO:0003729);protein binding(GO:0005515);receptor signaling complex scaffold activity(GO:0030159)
HUMAN|HGNC=11960|UniProtKB=Q6Y7W6	GIGYF2	GRB10-interacting GYF protein 2;GIGYF2;ortholog	RNA binding(GO:0003723);protein binding(GO:0005515);cadherin binding(GO:0045296);proline-rich region binding(GO:0070064)
HUMAN|HGNC=30677|UniProtKB=Q92917	GPKOW	G-patch domain and KOW motifs-containing protein;GPKOW;ortholog	RNA binding(GO:0003723);protein binding(GO:0005515)
HUMAN|HGNC=31394|UniProtKB=Q6P1K8	GTF2H2C	General transcription factor IIH subunit 2-like protein;GTF2H2C;ortholog	nucleic acid binding(GO:0003676);protein binding(GO:0005515);zinc ion binding(GO:0008270)
HUMAN|HGNC=4658|UniProtKB=Q92759	GTF2H4	General transcription factor IIH subunit 4;GTF2H4;ortholog	ATPase activator activity(GO:0001671);double-stranded DNA binding(GO:0003690);protein kinase activity(GO:0004672);protein binding(GO:0005515);DNA-dependent ATPase activity(GO:0008094);RNA polymerase II CTD heptapeptide repeat kinase activity(GO:0008353);RNA polymerase II general transcription initiation factor activity(GO:0016251)
HUMAN|HGNC=4659|UniProtKB=P78347	GTF2I	General transcription factor II-I;GTF2I;ortholog	DNA binding(GO:0003677);DNA-binding transcription factor activity(GO:0003700);protein binding(GO:0005515)
HUMAN|HGNC=4839|UniProtKB=P51610	HCFC1	Host cell factor 1;HCFC1;ortholog	DNA-binding transcription activator activity, RNA polymerase II-specific(GO:0001228);chromatin binding(GO:0003682);transcription coactivator activity(GO:0003713);protein binding(GO:0005515);protein binding, bridging(GO:0030674);activating transcription factor binding(GO:0033613);identical protein binding(GO:0042802);histone acetyltransferase activity (H4-K5 specific)(GO:0043995);histone acetyltransferase activity (H4-K8 specific)(GO:0043996);cadherin binding(GO:0045296);histone acetyltransferase activity (H4-K16 specific)(GO:0046972);sequence-specific double-stranded DNA binding(GO:1990837)
HUMAN|HGNC=4853|UniProtKB=Q92769	HDAC2	Histone deacetylase 2;HDAC2;ortholog	RNA polymerase II proximal promoter sequence-specific DNA binding(GO:0000978);RNA polymerase II distal enhancer sequence-specific DNA binding(GO:0000980);RNA polymerase II repressing transcription factor binding(GO:0001103);chromatin binding(GO:0003682);RNA binding(GO:0003723);histone deacetylase activity(GO:0004407);protein binding(GO:0005515);transcription factor binding(GO:0008134);deacetylase activity(GO:0019213);enzyme binding(GO:0019899);heat shock protein binding(GO:0031072);nucleosomal DNA binding(GO:0031492);NAD-dependent histone deacetylase activity (H3-K14 specific)(GO:0032041);protein deacetylase activity(GO:0033558);histone deacetylase binding(GO:0042826);sequence-specific DNA binding(GO:0043565);NF-kappaB binding(GO:0051059);promoter-specific chromatin binding(GO:1990841)
HUMAN|HGNC=5031|UniProtKB=P09651	HNRNPA1	Heterogeneous nuclear ribonucleoprotein A1;HNRNPA1;ortholog	single-stranded DNA binding(GO:0003697);RNA binding(GO:0003723);single-stranded RNA binding(GO:0003727);mRNA binding(GO:0003729);protein binding(GO:0005515);protein domain specific binding(GO:0019904);miRNA binding(GO:0035198);pre-mRNA binding(GO:0036002);identical protein binding(GO:0042802);telomeric repeat-containing RNA binding(GO:0061752);G-rich strand telomeric DNA binding(GO:0098505)
HUMAN|HGNC=5033|UniProtKB=P22626	HNRNPA2B1	Heterogeneous nuclear ribonucleoproteins A2/B1;HNRNPA2B1;ortholog	RNA binding(GO:0003723);mRNA binding(GO:0003729);mRNA 3'-UTR binding(GO:0003730);protein binding(GO:0005515);miRNA binding(GO:0035198);identical protein binding(GO:0042802);single-stranded telomeric DNA binding(GO:0043047);pre-mRNA intronic binding(GO:0097157);G-rich strand telomeric DNA binding(GO:0098505);N6-methyladenosine-containing RNA binding(GO:1990247)
HUMAN|HGNC=24941|UniProtKB=P51991	HNRNPA3	Heterogeneous nuclear ribonucleoprotein A3;HNRNPA3;ortholog	RNA binding(GO:0003723);mRNA binding(GO:0003729);protein binding(GO:0005515);RNA transmembrane transporter activity(GO:0051033)
HUMAN|HGNC=5039|UniProtKB=P52597	HNRNPF	Heterogeneous nuclear ribonucleoprotein F;HNRNPF;ortholog	nucleic acid binding(GO:0003676);RNA binding(GO:0003723);single-stranded RNA binding(GO:0003727);protein binding(GO:0005515);TBP-class protein binding(GO:0017025)
HUMAN|HGNC=5041|UniProtKB=P31943	HNRNPH1	Heterogeneous nuclear ribonucleoprotein H;HNRNPH1;ortholog	nucleic acid binding(GO:0003676);RNA binding(GO:0003723);protein binding(GO:0005515);poly(U) RNA binding(GO:0008266)
HUMAN|HGNC=5043|UniProtKB=P31942	HNRNPH3	Heterogeneous nuclear ribonucleoprotein H3;HNRNPH3;ortholog	RNA binding(GO:0003723);protein binding(GO:0005515)
HUMAN|HGNC=5045|UniProtKB=P14866	HNRNPL	Heterogeneous nuclear ribonucleoprotein L;HNRNPL;ortholog	RNA binding(GO:0003723);mRNA 3'-UTR binding(GO:0003730);protein binding(GO:0005515);transcription regulatory region DNA binding(GO:0044212);pre-mRNA intronic binding(GO:0097157);mRNA CDS binding(GO:1990715)
HUMAN|HGNC=5047|UniProtKB=O43390	HNRNPR	Heterogeneous nuclear ribonucleoprotein R;HNRNPR;ortholog	RNA binding(GO:0003723);mRNA binding(GO:0003729);mRNA 3'-UTR binding(GO:0003730);protein binding(GO:0005515)
HUMAN|HGNC=5244|UniProtKB=P38646	HSPA9	Stress-70 protein, mitochondrial;HSPA9;ortholog	RNA binding(GO:0003723);protein binding(GO:0005515);ATP binding(GO:0005524);ATPase activity(GO:0016887);heat shock protein binding(GO:0031072);ubiquitin protein ligase binding(GO:0031625);ATPase activity, coupled(GO:0042623);protein folding chaperone(GO:0044183);unfolded protein binding(GO:0051082);misfolded protein binding(GO:0051787)
HUMAN|HGNC=5395|UniProtKB=Q16666	IFI16	Gamma-interferon-inducible protein 16;IFI16;ortholog	RNA polymerase II proximal promoter sequence-specific DNA binding(GO:0000978);DNA-binding transcription repressor activity, RNA polymerase II-specific(GO:0001227);double-stranded DNA binding(GO:0003690);RNA binding(GO:0003723);protein binding(GO:0005515);transcription factor binding(GO:0008134);identical protein binding(GO:0042802)
HUMAN|HGNC=28866|UniProtKB=Q9NZI8	IGF2BP1	Insulin-like growth factor 2 mRNA-binding protein 1;IGF2BP1;ortholog	RNA binding(GO:0003723);mRNA binding(GO:0003729);mRNA 3'-UTR binding(GO:0003730);protein binding(GO:0005515);translation regulator activity(GO:0045182);mRNA 5'-UTR binding(GO:0048027)
HUMAN|HGNC=6037|UniProtKB=Q12905	ILF2	Interleukin enhancer-binding factor 2;ILF2;ortholog	DNA binding(GO:0003677);RNA binding(GO:0003723);double-stranded RNA binding(GO:0003725);protein binding(GO:0005515);ATP binding(GO:0005524);transferase activity(GO:0016740)
HUMAN|HGNC=6038|UniProtKB=Q12906	ILF3	Interleukin enhancer-binding factor 3;ILF3;ortholog	DNA binding(GO:0003677);RNA binding(GO:0003723);double-stranded RNA binding(GO:0003725);single-stranded RNA binding(GO:0003727);protein binding(GO:0005515);AU-rich element binding(GO:0017091)
HUMAN|HGNC=6402|UniProtKB=O00410	IPO5	Importin-5;IPO5;ortholog	RNA binding(GO:0003723);GTPase inhibitor activity(GO:0005095);protein binding(GO:0005515);nuclear localization sequence binding(GO:0008139);Ran GTPase binding(GO:0008536);protein transporter activity(GO:0008565)
HUMAN|HGNC=17016|UniProtKB=O95251	KAT7	Histone acetyltransferase KAT7;KAT7;ortholog	DNA-binding transcription factor activity, RNA polymerase II-specific(GO:0000981);DNA replication origin binding(GO:0003688);histone acetyltransferase activity(GO:0004402);protein binding(GO:0005515);zinc ion binding(GO:0008270);H4 histone acetyltransferase activity(GO:0010485);histone binding(GO:0042393);transcription regulatory region DNA binding(GO:0044212)
HUMAN|HGNC=18116|UniProtKB=Q07666	KHDRBS1	KH domain-containing, RNA-binding, signal transduction-associated protein 1;KHDRBS1;ortholog	DNA binding(GO:0003677);RNA binding(GO:0003723);SH3/SH2 adaptor activity(GO:0005070);protein binding(GO:0005515);poly(A) binding(GO:0008143);poly(U) RNA binding(GO:0008266);SH3 domain binding(GO:0017124);protein domain specific binding(GO:0019904);identical protein binding(GO:0042802);protein-containing complex binding(GO:0044877)
HUMAN|HGNC=6316|UniProtKB=Q92945	KHSRP	Far upstream element-binding protein 2;KHSRP;ortholog	nucleic acid binding(GO:0003676);DNA binding(GO:0003677);RNA binding(GO:0003723);protein binding(GO:0005515);mRNA 3'-UTR AU-rich region binding(GO:0035925)
HUMAN|HGNC=13339|UniProtKB=O95239	KIF4A	Chromosome-associated kinesin KIF4A;KIF4A;ortholog	DNA binding(GO:0003677);microtubule motor activity(GO:0003777);protein binding(GO:0005515);ATP binding(GO:0005524);microtubule binding(GO:0008017);ATPase activity(GO:0016887)
HUMAN|HGNC=29531|UniProtKB=Q6PKG0	LARP1	La-related protein 1;LARP1;ortholog	RNA cap binding(GO:0000339);RNA binding(GO:0003723);mRNA 3'-UTR binding(GO:0003730);protein binding(GO:0005515);eukaryotic initiation factor 4E binding(GO:0008190);translation activator activity(GO:0008494);translation initiation factor binding(GO:0031369);cadherin binding(GO:0045296);mRNA 5'-UTR binding(GO:0048027)
HUMAN|HGNC=24320|UniProtKB=Q71RC2	LARP4	La-related protein 4;LARP4;ortholog	RNA binding(GO:0003723);protein binding(GO:0005515);poly(A) binding(GO:0008143)
HUMAN|HGNC=28987|UniProtKB=Q92615	LARP4B	La-related protein 4B;LARP4B;ortholog	RNA binding(GO:0003723);protein binding(GO:0005515)
HUMAN|HGNC=15714|UniProtKB=P42704	LRPPRC	Leucine-rich PPR motif-containing protein, mitochondrial;LRPPRC;ortholog	single-stranded DNA binding(GO:0003697);RNA binding(GO:0003723);protein binding(GO:0005515);microtubule binding(GO:0008017);ubiquitin protein ligase binding(GO:0031625);beta-tubulin binding(GO:0048487)
HUMAN|HGNC=24489|UniProtKB=Q8ND56	LSM14A	Protein LSM14 homolog A;LSM14A;ortholog	double-stranded DNA binding(GO:0003690);RNA binding(GO:0003723);double-stranded RNA binding(GO:0003725);single-stranded RNA binding(GO:0003727)
HUMAN|HGNC=24309|UniProtKB=O95232	LUC7L3	Luc7-like protein 3;LUC7L3;ortholog	DNA binding(GO:0003677);RNA binding(GO:0003723);mRNA binding(GO:0003729);protein binding(GO:0005515)
HUMAN|HGNC=13664|UniProtKB=Q9UPN3	MACF1	Microtubule-actin cross-linking factor 1, isoforms 1/2/3/5;MACF1;ortholog	RNA binding(GO:0003723);actin binding(GO:0003779);structural molecule activity(GO:0005198);calcium ion binding(GO:0005509);protein binding(GO:0005515);microtubule binding(GO:0008017);ATPase activity(GO:0016887);cadherin binding(GO:0045296);microtubule minus-end binding(GO:0051011);actin filament binding(GO:0051015)
HUMAN|HGNC=6912|UniProtKB=P43243	MATR3	Matrin-3;MATR3;ortholog	nucleic acid binding(GO:0003676);RNA binding(GO:0003723);structural molecule activity(GO:0005198);protein binding(GO:0005515);zinc ion binding(GO:0008270);miRNA binding(GO:0035198);identical protein binding(GO:0042802)
HUMAN|HGNC=6945|UniProtKB=P25205	MCM3	DNA replication licensing factor MCM3;MCM3;ortholog	DNA binding(GO:0003677);helicase activity(GO:0004386);protein binding(GO:0005515);ATP binding(GO:0005524)
HUMAN|HGNC=9234|UniProtKB=Q15648	MED1	Mediator of RNA polymerase II transcription subunit 1;MED1;ortholog	RNA polymerase II proximal promoter sequence-specific DNA binding(GO:0000978);RNA polymerase II core promoter sequence-specific DNA binding(GO:0000979);chromatin binding(GO:0003682);transcription coregulator activity(GO:0003712);transcription coactivator activity(GO:0003713);transcription corepressor activity(GO:0003714);protein binding(GO:0005515);transcription factor binding(GO:0008134);nuclear receptor binding(GO:0016922);estrogen receptor binding(GO:0030331);nuclear receptor transcription coactivator activity(GO:0030374);thyroid hormone receptor coactivator activity(GO:0030375);chromatin DNA binding(GO:0031490);nuclear hormone receptor binding(GO:0035257);mediator complex binding(GO:0036033);signaling receptor activity(GO:0038023);vitamin D receptor binding(GO:0042809);retinoic acid receptor binding(GO:0042974);peroxisome proliferator activated receptor binding(GO:0042975);thyroid hormone receptor binding(GO:0046966);LBD domain binding(GO:0050693);ubiquitin protein ligase activity(GO:0061630)
HUMAN|HGNC=20247|UniProtKB=Q7L2J0	MEPCE	7SK snRNA methylphosphate capping enzyme;MEPCE;ortholog	RNA binding(GO:0003723);O-methyltransferase activity(GO:0008171);RNA methyltransferase activity(GO:0008173);S-adenosylmethionine-dependent methyltransferase activity(GO:0008757);snRNA binding(GO:0017069)
HUMAN|HGNC=7107|UniProtKB=P46013	MKI67	Proliferation marker protein Ki-67;MKI67;ortholog	DNA binding(GO:0003677);RNA binding(GO:0003723);protein binding(GO:0005515);ATP binding(GO:0005524);protein C-terminus binding(GO:0008022)
HUMAN|HGNC=7200|UniProtKB=Q9HCE1	MOV10	Putative helicase MOV-10;MOV10;ortholog	RNA binding(GO:0003723);protein binding(GO:0005515);ATP binding(GO:0005524);ATP-dependent 5'-3' RNA helicase activity(GO:0032575)
HUMAN|HGNC=7230|UniProtKB=P49959	MRE11A	Double-strand break repair protein MRE11;MRE11;ortholog	single-stranded DNA endodeoxyribonuclease activity(GO:0000014);DNA binding(GO:0003677);double-stranded DNA binding(GO:0003690);ATP-dependent DNA helicase activity(GO:0004003);nuclease activity(GO:0004518);endonuclease activity(GO:0004519);endodeoxyribonuclease activity(GO:0004520);protein binding(GO:0005515);protein C-terminus binding(GO:0008022);3'-5'-exodeoxyribonuclease activity(GO:0008296);3'-5' exonuclease activity(GO:0008408);5'-3' exonuclease activity(GO:0008409);hydrolase activity(GO:0016787);manganese ion binding(GO:0030145);identical protein binding(GO:0042802);cadherin binding(GO:0045296)
HUMAN|HGNC=18585|UniProtKB=Q96DH6	MSI2	RNA-binding protein Musashi homolog 2;MSI2;ortholog	RNA binding(GO:0003723);protein binding(GO:0005515);poly(U) RNA binding(GO:0008266)
HUMAN|HGNC=7546|UniProtKB=Q9BQG0	MYBBP1A	Myb-binding protein 1A;MYBBP1A;ortholog	DNA binding(GO:0003677);transcription corepressor activity(GO:0003714);RNA binding(GO:0003723);transcription factor binding(GO:0008134);sequence-specific DNA binding(GO:0043565);E-box binding(GO:0070888)
HUMAN|HGNC=7652|UniProtKB=O60934	NBN	Nibrin;NBN;ortholog	damaged DNA binding(GO:0003684);ATP-dependent DNA helicase activity(GO:0004003);protein binding(GO:0005515);transcription factor binding(GO:0008134);protein N-terminus binding(GO:0047485)
HUMAN|HGNC=7658|UniProtKB=Q09161	NCBP1	Nuclear cap-binding protein subunit 1;NCBP1;ortholog	RNA cap binding(GO:0000339);RNA 7-methylguanosine cap binding(GO:0000340);RNA binding(GO:0003723);protein binding(GO:0005515)
HUMAN|HGNC=24324|UniProtKB=Q8WX92	NELFB	Negative elongation factor B;NELFB;ortholog	RNA binding(GO:0003723);protein binding(GO:0005515)
HUMAN|HGNC=15934|UniProtKB=Q8IXH7	NELFCD	Negative elongation factor C/D;NELFCD;ortholog	RNA binding(GO:0003723);protein binding(GO:0005515)
HUMAN|HGNC=7785|UniProtKB=O00712	NFIB	Nuclear factor 1 B-type;NFIB;ortholog	RNA polymerase II proximal promoter sequence-specific DNA binding(GO:0000978);DNA-binding transcription factor activity, RNA polymerase II-specific(GO:0000981);DNA-binding transcription activator activity, RNA polymerase II-specific(GO:0001228);DNA binding(GO:0003677);DNA-binding transcription factor activity(GO:0003700);transcription corepressor activity(GO:0003714)
HUMAN|HGNC=28461|UniProtKB=Q9BVI4	NOC4L	Nucleolar complex protein 4 homolog;NOC4L;ortholog	DNA-binding transcription factor activity, RNA polymerase II-specific(GO:0000981);RNA binding(GO:0003723);protein binding(GO:0005515)
HUMAN|HGNC=17946|UniProtKB=Q9Y314	NOSIP	Nitric oxide synthase-interacting protein;NOSIP;ortholog	RNA binding(GO:0003723);protein binding(GO:0005515);ubiquitin protein ligase activity(GO:0061630)
HUMAN|HGNC=7978|UniProtKB=P04150	NR3C1	Glucocorticoid receptor;NR3C1;ortholog	RNA polymerase II proximal promoter sequence-specific DNA binding(GO:0000978);DNA-binding transcription factor activity, RNA polymerase II-specific(GO:0000981);core promoter binding(GO:0001047);DNA-binding transcription activator activity, RNA polymerase II-specific(GO:0001228);DNA binding(GO:0003677);DNA-binding transcription factor activity(GO:0003700);RNA binding(GO:0003723);nuclear receptor activity(GO:0004879);glucocorticoid receptor activity(GO:0004883);steroid binding(GO:0005496);protein binding(GO:0005515);zinc ion binding(GO:0008270);protein kinase binding(GO:0019901);SUMO binding(GO:0032183);Hsp90 protein binding(GO:0051879);steroid hormone binding(GO:1990239)
HUMAN|HGNC=16385|UniProtKB=Q96P11	NSUN5	Probable 28S rRNA (cytosine-C(5))-methyltransferase;NSUN5;ortholog	RNA binding(GO:0003723);methyltransferase activity(GO:0008168)
HUMAN|HGNC=17634|UniProtKB=Q7Z417	NUFIP2	Nuclear fragile X mental retardation-interacting protein 2;NUFIP2;ortholog	RNA binding(GO:0003723);protein binding(GO:0005515)
HUMAN|HGNC=8071|UniProtKB=Q9UBU9	NXF1	Nuclear RNA export factor 1;NXF1;ortholog	RNA binding(GO:0003723);mRNA binding(GO:0003729);protein binding(GO:0005515)
HUMAN|HGNC=24949|UniProtKB=Q01804	OTUD4	OTU domain-containing protein 4;OTUD4;ortholog	RNA binding(GO:0003723);thiol-dependent ubiquitin-specific protease activity(GO:0004843);protein binding(GO:0005515);protein-containing complex scaffold activity(GO:0032947);Lys63-specific deubiquitinase activity(GO:0061578)
HUMAN|HGNC=8548|UniProtKB=P07237	P4HB	Protein disulfide-isomerase;P4HB;ortholog	RNA binding(GO:0003723);protein disulfide isomerase activity(GO:0003756);procollagen-proline 4-dioxygenase activity(GO:0004656);integrin binding(GO:0005178);protein binding(GO:0005515);peptide disulfide oxidoreductase activity(GO:0015037);isomerase activity(GO:0016853);enzyme binding(GO:0019899);protein heterodimerization activity(GO:0046982)
HUMAN|HGNC=8554|UniProtKB=P11940	PABPC1	Polyadenylate-binding protein 1;PABPC1;ortholog	nucleic acid binding(GO:0003676);RNA binding(GO:0003723);mRNA 3'-UTR binding(GO:0003730);protein binding(GO:0005515);protein C-terminus binding(GO:0008022);poly(A) binding(GO:0008143);poly(U) RNA binding(GO:0008266);translation activator activity(GO:0008494)
HUMAN|HGNC=8557|UniProtKB=Q13310	PABPC4	Polyadenylate-binding protein 4;PABPC4;ortholog	RNA binding(GO:0003723);mRNA 3'-UTR binding(GO:0003730);protein binding(GO:0005515);poly(A) binding(GO:0008143);poly(U) RNA binding(GO:0008266)
HUMAN|HGNC=8565|UniProtKB=Q86U42	PABPN1	Polyadenylate-binding protein 2;PABPN1;ortholog	RNA binding(GO:0003723);protein binding(GO:0005515);RNA polymerase binding(GO:0070063)
HUMAN|HGNC=13579|UniProtKB=Q9Y5B6	PAXBP1	PAX3- and PAX7-binding protein 1;PAXBP1;ortholog	RNA polymerase II proximal promoter sequence-specific DNA binding(GO:0000978);DNA binding(GO:0003677);DNA-binding transcription factor activity(GO:0003700);transcription factor binding(GO:0008134)
HUMAN|HGNC=18000|UniProtKB=Q7RTV0	PHF5A	PHD finger-like domain-containing protein 5A;PHF5A;ortholog	DNA binding(GO:0003677);DNA-binding transcription factor activity(GO:0003700);RNA binding(GO:0003723);metal ion binding(GO:0046872)
HUMAN|HGNC=9162|UniProtKB=Q9H307	PNN	Pinin;PNN;ortholog	DNA binding(GO:0003677);RNA binding(GO:0003723);structural molecule activity(GO:0005198);protein binding(GO:0005515)
HUMAN|HGNC=18801|UniProtKB=Q7Z3K3	POGZ	Pogo transposable element with ZNF domain;POGZ;ortholog	RNA polymerase II core promoter sequence-specific DNA binding(GO:0000979);DNA-binding transcription factor activity, RNA polymerase II-specific(GO:0000981);nucleic acid binding(GO:0003676);protein binding(GO:0005515);metal ion binding(GO:0046872)
HUMAN|HGNC=9175|UniProtKB=P28340	POLD1	DNA polymerase delta catalytic subunit;POLD1;ortholog	nucleotide binding(GO:0000166);DNA binding(GO:0003677);chromatin binding(GO:0003682);damaged DNA binding(GO:0003684);DNA-directed DNA polymerase activity(GO:0003887);protein binding(GO:0005515);3'-5'-exodeoxyribonuclease activity(GO:0008296);3'-5' exonuclease activity(GO:0008408);enzyme binding(GO:0019899);metal ion binding(GO:0046872);4 iron, 4 sulfur cluster binding(GO:0051539)
HUMAN|HGNC=9176|UniProtKB=P49005	POLD2	DNA polymerase delta subunit 2;POLD2;ortholog	DNA binding(GO:0003677);DNA-directed DNA polymerase activity(GO:0003887);protein binding(GO:0005515)
HUMAN|HGNC=9188|UniProtKB=P30876	POLR2B	DNA-directed RNA polymerase II subunit RPB2;POLR2B;ortholog	RNA polymerase II activity(GO:0001055);DNA binding(GO:0003677);chromatin binding(GO:0003682);RNA binding(GO:0003723);DNA-directed 5'-3' RNA polymerase activity(GO:0003899);protein binding(GO:0005515);ribonucleoside binding(GO:0032549);metal ion binding(GO:0046872)
HUMAN|HGNC=9284|UniProtKB=Q96QC0	PPP1R10	Serine/threonine-protein phosphatase 1 regulatory subunit 10;PPP1R10;ortholog	DNA binding(GO:0003677);RNA binding(GO:0003723);protein phosphatase inhibitor activity(GO:0004864);protein binding(GO:0005515);metal ion binding(GO:0046872)
HUMAN|HGNC=5187|UniProtKB=Q99873	PRMT1	Protein arginine N-methyltransferase 1;PRMT1;ortholog	RNA binding(GO:0003723);protein binding(GO:0005515);methyltransferase activity(GO:0008168);N-methyltransferase activity(GO:0008170);protein methyltransferase activity(GO:0008276);methyl-CpG binding(GO:0008327);histone-arginine N-methyltransferase activity(GO:0008469);protein-arginine N-methyltransferase activity(GO:0016274);enzyme binding(GO:0019899);protein-arginine omega-N monomethyltransferase activity(GO:0035241);protein-arginine omega-N asymmetric methyltransferase activity(GO:0035242);histone methyltransferase activity(GO:0042054);identical protein binding(GO:0042802);histone methyltransferase activity (H4-R3 specific)(GO:0044020);mitogen-activated protein kinase p38 binding(GO:0048273);S-adenosyl-L-methionine binding(GO:1904047)
HUMAN|HGNC=17348|UniProtKB=O43395	PRPF3	U4/U6 small nuclear ribonucleoprotein Prp3;PRPF3;ortholog	RNA binding(GO:0003723);protein binding(GO:0005515);identical protein binding(GO:0042802)
HUMAN|HGNC=15446|UniProtKB=Q8WWY3	PRPF31	U4/U6 small nuclear ribonucleoprotein Prp31;PRPF31;ortholog	RNA binding(GO:0003723);protein binding(GO:0005515);U4 snRNA binding(GO:0030621);U4atac snRNA binding(GO:0030622);ribonucleoprotein complex binding(GO:0043021);snRNP binding(GO:0070990)
HUMAN|HGNC=16463|UniProtKB=O75400	PRPF40A	Pre-mRNA-processing factor 40 homolog A;PRPF40A;ortholog	RNA binding(GO:0003723);protein binding(GO:0005515)
HUMAN|HGNC=15860|UniProtKB=O94906	PRPF6	Pre-mRNA-processing factor 6;PRPF6;ortholog	transcription coactivator activity(GO:0003713);RNA binding(GO:0003723);protein binding(GO:0005515);ribonucleoprotein complex binding(GO:0043021);androgen receptor binding(GO:0050681)
HUMAN|HGNC=13918|UniProtKB=P48634	PRRC2A	Protein PRRC2A;PRRC2A;ortholog	RNA binding(GO:0003723);protein binding(GO:0005515)
HUMAN|HGNC=24903|UniProtKB=Q9Y520	PRRC2C	Protein PRRC2C;PRRC2C;ortholog	RNA binding(GO:0003723);protein C-terminus binding(GO:0008022)
HUMAN|HGNC=14957|UniProtKB=Q14671	PUM1	Pumilio homolog 1;PUM1;ortholog	RNA binding(GO:0003723);mRNA 3'-UTR binding(GO:0003730);protein binding(GO:0005515);miRNA binding(GO:0035198)
HUMAN|HGNC=9701|UniProtKB=Q00577	PURA	Transcriptional activator protein Pur-alpha;PURA;ortholog	translation repressor activity, mRNA regulatory element binding(GO:0000900);RNA polymerase II regulatory region sequence-specific DNA binding(GO:0000977);DNA-binding transcription factor activity, RNA polymerase II-specific(GO:0000981);DNA-binding transcription repressor activity, RNA polymerase II-specific(GO:0001227);double-stranded DNA binding(GO:0003690);double-stranded telomeric DNA binding(GO:0003691);single-stranded DNA binding(GO:0003697);RNA binding(GO:0003723);protein binding(GO:0005515);transcription factor binding(GO:0008134);purine-rich negative regulatory element binding(GO:0032422);sequence-specific DNA binding(GO:0043565);SMAD binding(GO:0046332)
HUMAN|HGNC=9702|UniProtKB=Q96QR8	PURB	Transcriptional activator protein Pur-beta;PURB;ortholog	RNA polymerase II regulatory region sequence-specific DNA binding(GO:0000977);DNA-binding transcription factor activity, RNA polymerase II-specific(GO:0000981);DNA-binding transcription repressor activity, RNA polymerase II-specific(GO:0001227);double-stranded DNA binding(GO:0003690);single-stranded DNA binding(GO:0003697);RNA binding(GO:0003723);mRNA binding(GO:0003729);transcription factor binding(GO:0008134);purine-rich negative regulatory element binding(GO:0032422);sequence-specific DNA binding(GO:0043565);SMAD binding(GO:0046332)
HUMAN|HGNC=9816|UniProtKB=Q92878	RAD50	DNA repair protein RAD50;RAD50;ortholog	single-stranded DNA endodeoxyribonuclease activity(GO:0000014);DNA binding(GO:0003677);double-stranded telomeric DNA binding(GO:0003691);ATP-dependent DNA helicase activity(GO:0004003);adenylate kinase activity(GO:0004017);protein binding(GO:0005515);ATP binding(GO:0005524);3'-5' exonuclease activity(GO:0008408);ATPase activity(GO:0016887);protein binding, bridging(GO:0030674);single-stranded telomeric DNA binding(GO:0043047);metal ion binding(GO:0046872);G-quadruplex DNA binding(GO:0051880)
HUMAN|HGNC=9848|UniProtKB=P49792	RANBP2	E3 SUMO-protein ligase RanBP2;RANBP2;ortholog	RNA binding(GO:0003723);GTPase activator activity(GO:0005096);protein binding(GO:0005515);Ran GTPase binding(GO:0008536);SUMO transferase activity(GO:0019789);protein-containing complex binding(GO:0044877);metal ion binding(GO:0046872)
HUMAN|HGNC=30296|UniProtKB=Q8IY67	RAVER1	Ribonucleoprotein PTB-binding 1;RAVER1;ortholog	nucleic acid binding(GO:0003676);RNA binding(GO:0003723);protein binding(GO:0005515)
HUMAN|HGNC=9906|UniProtKB=O43251	RBFOX2	RNA binding protein fox-1 homolog 2;RBFOX2;ortholog	transcription corepressor activity(GO:0003714);RNA binding(GO:0003723);mRNA binding(GO:0003729);protein binding(GO:0005515);transcription factor binding(GO:0008134)
HUMAN|HGNC=9896|UniProtKB=P98175	RBM10	RNA-binding protein 10;RBM10;ortholog	RNA binding(GO:0003723);protein binding(GO:0005515);miRNA binding(GO:0035198);identical protein binding(GO:0042802);metal ion binding(GO:0046872)
HUMAN|HGNC=32310|UniProtKB=Q8IXT5	RBM12B	RNA-binding protein 12B;RBM12B;ortholog	nucleic acid binding(GO:0003676);RNA binding(GO:0003723);protein binding(GO:0005515)
HUMAN|HGNC=14959|UniProtKB=Q96T37	RBM15	Putative RNA-binding protein 15;RBM15;ortholog	RNA binding(GO:0003723);mRNA binding(GO:0003729);protein binding(GO:0005515)
HUMAN|HGNC=23244|UniProtKB=P49756	RBM25	RNA-binding protein 25;RBM25;ortholog	nucleic acid binding(GO:0003676);RNA binding(GO:0003723);mRNA binding(GO:0003729);protein binding(GO:0005515)
HUMAN|HGNC=29243|UniProtKB=Q9P2N5	RBM27	RNA-binding protein 27;RBM27;ortholog	RNA binding(GO:0003723);mRNA binding(GO:0003729);metal ion binding(GO:0046872)
HUMAN|HGNC=27223|UniProtKB=Q96EV2	RBM33	RNA-binding protein 33;RBM33;ortholog	nucleic acid binding(GO:0003676);RNA binding(GO:0003723)
HUMAN|HGNC=30358|UniProtKB=A0AV96	RBM47	RNA-binding protein 47;RBM47;ortholog	RNA binding(GO:0003723);mRNA binding(GO:0003729)
HUMAN|HGNC=9902|UniProtKB=P52756	RBM5	RNA-binding protein 5;RBM5;ortholog	nucleic acid binding(GO:0003676);DNA binding(GO:0003677);RNA binding(GO:0003723);mRNA binding(GO:0003729);protein binding(GO:0005515);metal ion binding(GO:0046872)
HUMAN|HGNC=18518|UniProtKB=O75792	RNASEH2A	Ribonuclease H2 subunit A;RNASEH2A;ortholog	RNA binding(GO:0003723);RNA-DNA hybrid ribonuclease activity(GO:0004523);ribonuclease activity(GO:0004540);metal ion binding(GO:0046872)
HUMAN|HGNC=25671|UniProtKB=Q5TBB1	RNASEH2B	Ribonuclease H2 subunit B;RNASEH2B;ortholog	RNA-DNA hybrid ribonuclease activity(GO:0004523)
HUMAN|HGNC=24116|UniProtKB=Q8TDP1	RNASEH2C	Ribonuclease H2 subunit C;RNASEH2C;ortholog	
HUMAN|HGNC=16867|UniProtKB=O75150	RNF40	E3 ubiquitin-protein ligase BRE1B;RNF40;ortholog	mRNA 3'-UTR binding(GO:0003730);ubiquitin-protein transferase activity(GO:0004842);protein binding(GO:0005515);syntaxin-1 binding(GO:0017075);ubiquitin conjugating enzyme binding(GO:0031624);ubiquitin protein ligase binding(GO:0031625);protein homodimerization activity(GO:0042803);protein-containing complex binding(GO:0044877);metal ion binding(GO:0046872)
HUMAN|HGNC=10075|UniProtKB=O43148	RNMT	mRNA cap guanine-N7 methyltransferase;RNMT;ortholog	RNA binding(GO:0003723);mRNA (guanine-N7-)-methyltransferase activity(GO:0004482);protein binding(GO:0005515)
HUMAN|HGNC=10080|UniProtKB=Q15287	RNPS1	RNA-binding protein with serine-rich domain 1;RNPS1;ortholog	RNA binding(GO:0003723);mRNA 3'-UTR binding(GO:0003730);protein binding(GO:0005515)
HUMAN|HGNC=10311|UniProtKB=Q02543	RPL18A	60S ribosomal protein L18a;RPL18A;ortholog	RNA binding(GO:0003723);structural constituent of ribosome(GO:0003735);protein binding(GO:0005515)
HUMAN|HGNC=17688|UniProtKB=P78346	RPP30	Ribonuclease P protein subunit p30;RPP30;ortholog	RNA binding(GO:0003723);ribonuclease P activity(GO:0004526);ribonuclease activity(GO:0004540);protein binding(GO:0005515)
HUMAN|HGNC=24534|UniProtKB=O76021	RSL1D1	Ribosomal L1 domain-containing protein 1;RSL1D1;ortholog	RNA binding(GO:0003723);mRNA 3'-UTR binding(GO:0003730);cadherin binding(GO:0045296);mRNA 5'-UTR binding(GO:0048027)
HUMAN|HGNC=26935|UniProtKB=Q9Y3I0	RTCB	tRNA-splicing ligase RtcB homolog;RTCB;ortholog	RNA binding(GO:0003723);RNA ligase (ATP) activity(GO:0003972);protein binding(GO:0005515);ATP binding(GO:0005524);vinculin binding(GO:0017166);metal ion binding(GO:0046872)
HUMAN|HGNC=23169|UniProtKB=Q9Y224	RTRAF	RNA transcription, translation and transport factor protein;RTRAF;ortholog	RNA polymerase II complex binding(GO:0000993);RNA binding(GO:0003723);protein binding(GO:0005515);identical protein binding(GO:0042802)
HUMAN|HGNC=10538|UniProtKB=O43290	SART1	U4/U6.U5 tri-snRNP-associated protein 1;SART1;ortholog	RNA binding(GO:0003723);protein binding(GO:0005515)
HUMAN|HGNC=17860|UniProtKB=Q8NC51	SERBP1	Plasminogen activator inhibitor 1 RNA-binding protein;SERBP1;ortholog	RNA binding(GO:0003723);mRNA 3'-UTR binding(GO:0003730);protein binding(GO:0005515);SUMO binding(GO:0032183);cadherin binding(GO:0045296)
HUMAN|HGNC=12950|UniProtKB=Q15637	SF1	Splicing factor 1;SF1;ortholog	transcription corepressor activity(GO:0003714);RNA binding(GO:0003723);protein binding(GO:0005515);zinc ion binding(GO:0008270);identical protein binding(GO:0042802);pre-mRNA branch point binding(GO:0045131)
HUMAN|HGNC=10765|UniProtKB=Q15459	SF3A1	Splicing factor 3A subunit 1;SF3A1;ortholog	RNA binding(GO:0003723);protein binding(GO:0005515)
HUMAN|HGNC=10766|UniProtKB=Q15428	SF3A2	Splicing factor 3A subunit 2;SF3A2;ortholog	nucleic acid binding(GO:0003676);RNA binding(GO:0003723);protein binding(GO:0005515);zinc ion binding(GO:0008270)
HUMAN|HGNC=10767|UniProtKB=Q12874	SF3A3	Splicing factor 3A subunit 3;SF3A3;ortholog	RNA binding(GO:0003723);protein binding(GO:0005515);zinc ion binding(GO:0008270)
HUMAN|HGNC=10768|UniProtKB=O75533	SF3B1	Splicing factor 3B subunit 1;SF3B1;ortholog	RNA binding(GO:0003723);mRNA binding(GO:0003729);protein binding(GO:0005515)
HUMAN|HGNC=10769|UniProtKB=Q13435	SF3B2	Splicing factor 3B subunit 2;SF3B2;ortholog	RNA binding(GO:0003723);protein binding(GO:0005515)
HUMAN|HGNC=10770|UniProtKB=Q15393	SF3B3	Splicing factor 3B subunit 3;SF3B3;ortholog	nucleic acid binding(GO:0003676);protein binding(GO:0005515);protein-containing complex binding(GO:0044877)
HUMAN|HGNC=30096|UniProtKB=Q9Y3B4	SF3B6	Splicing factor 3B subunit 6;SF3B6;ortholog	RNA binding(GO:0003723);mRNA binding(GO:0003729);protein binding(GO:0005515)
HUMAN|HGNC=11100|UniProtKB=P51532	SMARCA4	Transcription activator BRG1;SMARCA4;ortholog	RNA polymerase II proximal promoter sequence-specific DNA binding(GO:0000978);RNA polymerase II distal enhancer sequence-specific DNA binding(GO:0000980);RNA polymerase I CORE element sequence-specific DNA binding(GO:0001164);p53 binding(GO:0002039);transcription coactivator activity(GO:0003713);transcription corepressor activity(GO:0003714);RNA binding(GO:0003723);helicase activity(GO:0004386);protein binding(GO:0005515);ATP binding(GO:0005524);DNA-dependent ATPase activity(GO:0008094);transcription factor binding(GO:0008134);hydrolase activity, acting on acid anhydrides(GO:0016817);ATPase activity(GO:0016887);Tat protein binding(GO:0030957);nucleosomal DNA binding(GO:0031492);histone binding(GO:0042393);protein N-terminus binding(GO:0047485);androgen receptor binding(GO:0050681);DNA polymerase binding(GO:0070182);lysine-acetylated histone binding(GO:0070577)
HUMAN|HGNC=11103|UniProtKB=Q12824	SMARCB1	SWI/SNF-related matrix-associated actin-dependent regulator of chromatin subfamily B member 1;SMARCB1;ortholog	RNA polymerase II proximal promoter sequence-specific DNA binding(GO:0000978);RNA polymerase II distal enhancer sequence-specific DNA binding(GO:0000980);RNA polymerase I CORE element sequence-specific DNA binding(GO:0001164);p53 binding(GO:0002039);DNA binding(GO:0003677);transcription coactivator activity(GO:0003713);protein binding(GO:0005515);Tat protein binding(GO:0030957);nucleosomal DNA binding(GO:0031492)
HUMAN|HGNC=11104|UniProtKB=Q92922	SMARCC1	SWI/SNF complex subunit SMARCC1;SMARCC1;ortholog	RNA polymerase II proximal promoter sequence-specific DNA binding(GO:0000978);RNA polymerase II distal enhancer sequence-specific DNA binding(GO:0000980);DNA-binding transcription factor activity, RNA polymerase II-specific(GO:0000981);transcription coactivator activity(GO:0003713);protein binding(GO:0005515);nucleosomal DNA binding(GO:0031492);protein N-terminus binding(GO:0047485)
HUMAN|HGNC=11105|UniProtKB=Q8TAQ2	SMARCC2	SWI/SNF complex subunit SMARCC2;SMARCC2;ortholog	RNA polymerase II proximal promoter sequence-specific DNA binding(GO:0000978);RNA polymerase II distal enhancer sequence-specific DNA binding(GO:0000980);DNA-binding transcription factor activity, RNA polymerase II-specific(GO:0000981);DNA binding(GO:0003677);chromatin binding(GO:0003682);transcription coactivator activity(GO:0003713);protein binding(GO:0005515);nucleosomal DNA binding(GO:0031492)
HUMAN|HGNC=30859|UniProtKB=O75643	SNRNP200	U5 small nuclear ribonucleoprotein 200 kDa helicase;SNRNP200;ortholog	RNA binding(GO:0003723);ATP-dependent RNA helicase activity(GO:0004004);helicase activity(GO:0004386);protein binding(GO:0005515);ATP binding(GO:0005524);ATP-dependent helicase activity(GO:0008026);identical protein binding(GO:0042802)
HUMAN|HGNC=30857|UniProtKB=Q96DI7	SNRNP40	U5 small nuclear ribonucleoprotein 40 kDa protein;SNRNP40;ortholog	RNA binding(GO:0003723);protein binding(GO:0005515)
HUMAN|HGNC=11150|UniProtKB=P08621	SNRNP70	U1 small nuclear ribonucleoprotein 70 kDa;SNRNP70;ortholog	RNA binding(GO:0003723);mRNA binding(GO:0003729);protein binding(GO:0005515);U1 snRNA binding(GO:0030619)
HUMAN|HGNC=11151|UniProtKB=P09012	SNRPA	U1 small nuclear ribonucleoprotein A;SNRPA;ortholog	RNA binding(GO:0003723);protein binding(GO:0005515);U1 snRNA binding(GO:0030619);snRNA stem-loop binding(GO:0035614);identical protein binding(GO:0042802);U1 snRNP binding(GO:1990446)
HUMAN|HGNC=11152|UniProtKB=P09661	SNRPA1	U2 small nuclear ribonucleoprotein A';SNRPA1;ortholog	RNA binding(GO:0003723);protein binding(GO:0005515);U2 snRNA binding(GO:0030620)
HUMAN|HGNC=11157|UniProtKB=P09234	SNRPC	U1 small nuclear ribonucleoprotein C;SNRPC;ortholog	RNA binding(GO:0003723);single-stranded RNA binding(GO:0003727);mRNA binding(GO:0003729);protein binding(GO:0005515);zinc ion binding(GO:0008270);U1 snRNA binding(GO:0030619);pre-mRNA 5'-splice site binding(GO:0030627);protein homodimerization activity(GO:0042803);U1 snRNP binding(GO:1990446)
HUMAN|HGNC=11158|UniProtKB=P62314	SNRPD1	Small nuclear ribonucleoprotein Sm D1;SNRPD1;ortholog	RNA binding(GO:0003723);protein binding(GO:0005515);U1 snRNP binding(GO:1990446)
HUMAN|HGNC=11159|UniProtKB=P62316	SNRPD2,SNR	Small nuclear ribonucleoprotein Sm D2;SNRPD2;ortholog	RNA binding(GO:0003723);protein binding(GO:0005515)
HUMAN|HGNC=11160|UniProtKB=P62318	SNRPD3	Small nuclear ribonucleoprotein Sm D3;SNRPD3;ortholog	RNA binding(GO:0003723);protein binding(GO:0005515);enzyme binding(GO:0019899);telomerase RNA binding(GO:0070034);histone pre-mRNA DCP binding(GO:0071208);U7 snRNA binding(GO:0071209);U1 snRNP binding(GO:1990446)
HUMAN|HGNC=11161|UniProtKB=P62304	SNRPE	Small nuclear ribonucleoprotein E;SNRPE;ortholog	RNA binding(GO:0003723);protein binding(GO:0005515)
HUMAN|HGNC=11164|UniProtKB=P63162	SNRPN	Small nuclear ribonucleoprotein-associated protein N;SNRPN;ortholog	RNA binding(GO:0003723);protein binding(GO:0005515)
HUMAN|HGNC=11183|UniProtKB=P18583	SON	Protein SON;SON;ortholog	DNA binding(GO:0003677);RNA binding(GO:0003723);protein binding(GO:0005515);RS domain binding(GO:0050733)
HUMAN|HGNC=11303|UniProtKB=O76094	SRP72	Signal recognition particle subunit SRP72;SRP72;ortholog	RNA binding(GO:0003723);signal recognition particle binding(GO:0005047);protein binding(GO:0005515);7S RNA binding(GO:0008312);TPR domain binding(GO:0030911);ribosome binding(GO:0043022)
HUMAN|HGNC=16638|UniProtKB=Q8IYB3	SRRM1	Serine/arginine repetitive matrix protein 1;SRRM1;ortholog	DNA binding(GO:0003677);RNA binding(GO:0003723);protein binding(GO:0005515)
HUMAN|HGNC=16639|UniProtKB=Q9UQ35	SRRM2	Serine/arginine repetitive matrix protein 2;SRRM2;ortholog	RNA binding(GO:0003723);protein N-terminus binding(GO:0047485);C2H2 zinc finger domain binding(GO:0070742)
HUMAN|HGNC=24101|UniProtKB=Q9BXP5	SRRT	Serrate RNA effector molecule homolog;SRRT;ortholog	nucleic acid binding(GO:0003676);DNA binding(GO:0003677);RNA binding(GO:0003723);protein binding(GO:0005515)
HUMAN|HGNC=10780|UniProtKB=Q07955	SRSF1	Serine/arginine-rich splicing factor 1;SRSF1;ortholog	RNA binding(GO:0003723);mRNA binding(GO:0003729);protein binding(GO:0005515);protein kinase B binding(GO:0043422);DNA topoisomerase binding(GO:0044547);RS domain binding(GO:0050733)
HUMAN|HGNC=16713|UniProtKB=O75494	SRSF10	Serine/arginine-rich splicing factor 10;SRSF10;ortholog	RNA binding(GO:0003723);protein binding(GO:0005515);RS domain binding(GO:0050733);unfolded protein binding(GO:0051082)
HUMAN|HGNC=10782|UniProtKB=Q05519	SRSF11	Serine/arginine-rich splicing factor 11;SRSF11;ortholog	RNA binding(GO:0003723);protein binding(GO:0005515)
HUMAN|HGNC=10783|UniProtKB=Q01130	SRSF2	Serine/arginine-rich splicing factor 2;SRSF2;ortholog	transcription corepressor activity(GO:0003714);RNA binding(GO:0003723);protein binding(GO:0005515)
HUMAN|HGNC=10788|UniProtKB=Q13247	SRSF6	Serine/arginine-rich splicing factor 6;SRSF6;ortholog	RNA binding(GO:0003723);protein binding(GO:0005515);pre-mRNA binding(GO:0036002)
HUMAN|HGNC=10789|UniProtKB=Q16629	SRSF7	Serine/arginine-rich splicing factor 7;SRSF7;ortholog	nucleic acid binding(GO:0003676);RNA binding(GO:0003723);protein binding(GO:0005515);zinc ion binding(GO:0008270);protein domain specific binding(GO:0019904)
HUMAN|HGNC=10791|UniProtKB=Q13242	SRSF9	Serine/arginine-rich splicing factor 9;SRSF9;ortholog	RNA binding(GO:0003723);protein binding(GO:0005515);protein domain specific binding(GO:0019904)
HUMAN|HGNC=16918|UniProtKB=O60506	SYNCRIP	Heterogeneous nuclear ribonucleoprotein Q;SYNCRIP;ortholog	RNA binding(GO:0003723);mRNA binding(GO:0003729);protein binding(GO:0005515);poly(A) binding(GO:0008143);mRNA 5'-UTR binding(GO:0048027)
HUMAN|HGNC=17165|UniProtKB=Q9UBB9	TFIP11	Tuftelin-interacting protein 11;TFIP11;ortholog	nucleic acid binding(GO:0003676);protein binding(GO:0005515)
HUMAN|HGNC=17008|UniProtKB=Q92547	TOPBP1	DNA topoisomerase 2-binding protein 1;TOPBP1;ortholog	DNA binding(GO:0003677);protein binding(GO:0005515);protein C-terminus binding(GO:0008022);identical protein binding(GO:0042802)
HUMAN|HGNC=10781|UniProtKB=P62995	TRA2B	Transformer-2 protein homolog beta;TRA2B;ortholog	RNA binding(GO:0003723);mRNA binding(GO:0003729);protein binding(GO:0005515);protein domain specific binding(GO:0019904);pre-mRNA binding(GO:0036002);identical protein binding(GO:0042802)
HUMAN|HGNC=30855|UniProtKB=O15042	U2SURP	U2 snRNP-associated SURP motif-containing protein;U2SURP;ortholog	RNA binding(GO:0003723);protein binding(GO:0005515)
HUMAN|HGNC=16806|UniProtKB=O95071	UBR5	E3 ubiquitin-protein ligase UBR5;UBR5;ortholog	RNA binding(GO:0003723);ubiquitin-protein transferase activity(GO:0004842);protein binding(GO:0005515);zinc ion binding(GO:0008270);ubiquitin-ubiquitin ligase activity(GO:0034450);ubiquitin binding(GO:0043130);ubiquitin protein ligase activity(GO:0061630)
HUMAN|HGNC=17854|UniProtKB=Q9HAU5	UPF2	Regulator of nonsense transcripts 2;UPF2;ortholog	RNA binding(GO:0003723);protein binding(GO:0005515);telomeric DNA binding(GO:0042162)
HUMAN|HGNC=12692|UniProtKB=P08670	VIM	Vimentin;VIM;ortholog	double-stranded RNA binding(GO:0003725);structural molecule activity(GO:0005198);structural constituent of cytoskeleton(GO:0005200);structural constituent of eye lens(GO:0005212);protein binding(GO:0005515);protein C-terminus binding(GO:0008022);protein domain specific binding(GO:0019904);identical protein binding(GO:0042802);scaffold protein binding(GO:0097110);keratin filament binding(GO:1990254)
HUMAN|HGNC=24500|UniProtKB=Q69YN4	virma	Protein virilizer homolog;VIRMA;ortholog	RNA binding(GO:0003723);protein binding(GO:0005515)
HUMAN|HGNC=16461|UniProtKB=Q9Y2W2	WBP11	WW domain-binding protein 11;WBP11;ortholog	single-stranded DNA binding(GO:0003697);RNA binding(GO:0003723);protein binding(GO:0005515);WW domain binding(GO:0050699)
HUMAN|HGNC=16405|UniProtKB=O43709	WBSCR22	Probable 18S rRNA (guanine-N(7))-methyltransferase;BUD23;ortholog	RNA binding(GO:0003723);methyltransferase activity(GO:0008168);rRNA (guanine) methyltransferase activity(GO:0016435);protein heterodimerization activity(GO:0046982)
HUMAN|HGNC=25651|UniProtKB=Q9C0J8	WDR33	pre-mRNA 3' end processing protein WDR33;WDR33;ortholog	RNA binding(GO:0003723)
HUMAN|HGNC=25489|UniProtKB=Q9ULM3	YEATS2	YEATS domain-containing protein 2;YEATS2;ortholog	DNA-binding transcription factor activity, RNA polymerase II-specific(GO:0000981);protein binding(GO:0005515);TBP-class protein binding(GO:0017025);histone binding(GO:0042393);modification-dependent protein binding(GO:0140030)
HUMAN|HGNC=17798|UniProtKB=P49750	YLPM1	YLP motif-containing protein 1;YLPM1;ortholog	RNA binding(GO:0003723);protein binding(GO:0005515)
HUMAN|HGNC=31675|UniProtKB=Q9Y5A9	YTHDF2	YTH domain-containing family protein 2;YTHDF2;ortholog	RNA binding(GO:0003723);protein binding(GO:0005515);N6-methyladenosine-containing RNA binding(GO:1990247)
HUMAN|HGNC=12856|UniProtKB=P25490	YY1	Transcriptional repressor protein YY1;YY1;ortholog	four-way junction DNA binding(GO:0000400);RNA polymerase II proximal promoter sequence-specific DNA binding(GO:0000978);RNA polymerase II distal enhancer sequence-specific DNA binding(GO:0000980);DNA-binding transcription factor activity, RNA polymerase II-specific(GO:0000981);proximal promoter sequence-specific DNA binding(GO:0000987);enhancer sequence-specific DNA binding(GO:0001158);DNA-binding transcription repressor activity, RNA polymerase II-specific(GO:0001227);nucleic acid binding(GO:0003676);DNA binding(GO:0003677);DNA-binding transcription factor activity(GO:0003700);transcription coactivator activity(GO:0003713);transcription corepressor activity(GO:0003714);RNA binding(GO:0003723);protein binding(GO:0005515);zinc ion binding(GO:0008270);sequence-specific DNA binding(GO:0043565);transcription regulatory region DNA binding(GO:0044212);SMAD binding(GO:0046332)
HUMAN|HGNC=29093|UniProtKB=O75152	ZC3H11A	Zinc finger CCCH domain-containing protein 11A;ZC3H11A;ortholog	DNA-binding transcription factor activity, RNA polymerase II-specific(GO:0000981);RNA binding(GO:0003723);mRNA binding(GO:0003729);protein binding(GO:0005515);metal ion binding(GO:0046872)
HUMAN|HGNC=20368|UniProtKB=Q5T200	ZC3H13	Zinc finger CCCH domain-containing protein 13;ZC3H13;ortholog	RNA binding(GO:0003723);protein binding(GO:0005515);metal ion binding(GO:0046872)
HUMAN|HGNC=17277|UniProtKB=Q96KR1	ZFR	Zinc finger RNA-binding protein;ZFR;ortholog	DNA binding(GO:0003677);RNA binding(GO:0003723);double-stranded RNA binding(GO:0003725);single-stranded RNA binding(GO:0003727);protein binding(GO:0005515);zinc ion binding(GO:0008270)
HUMAN|HGNC=28079|UniProtKB=Q86UK7	ZNF598	E3 ubiquitin-protein ligase ZNF598;ZNF598;ortholog	nucleic acid binding(GO:0003676);RNA binding(GO:0003723);protein binding(GO:0005515);ribosome binding(GO:0043022);metal ion binding(GO:0046872);ubiquitin protein ligase activity(GO:0061630)

Supplementary Table 4.1 Human proteins found enriched on system 1a relatively to system 2		
Gene ID	Mapped IDs	Gene Name
HUMAN|HGNC=10648|UniProtKB=Q12904	AIMP1	Aminoacyl tRNA synthase complex-interacting multifunctional protein 1;AIMP1;ortholog
HUMAN|HGNC=20609|UniProtKB=Q13155	AIMP2	Aminoacyl tRNA synthase complex-interacting multifunctional protein 2;AIMP2;ortholog
HUMAN|HGNC=11393|UniProtKB=O14965	AURKA	Aurora kinase A;AURKA;ortholog
HUMAN|HGNC=18673|UniProtKB=Q96JB5	CDK5RAP3	CDK5 regulatory subunit-associated protein 3;CDK5RAP3;ortholog
HUMAN|HGNC=2678|UniProtKB=P14868	DARS	Aspartate--tRNA ligase, cytoplasmic;DARS;ortholog
HUMAN|HGNC=3061|UniProtKB=P23919	DTYMK	Thymidylate kinase;DTYMK;ortholog
HUMAN|HGNC=3212|UniProtKB=O43324	EEF1E1	Eukaryotic translation elongation factor 1 epsilon-1;EEF1E1;ortholog
HUMAN|HGNC=3418|UniProtKB=P07814	EPRS	Bifunctional glutamate/proline--tRNA ligase;EPRS;ortholog
HUMAN|HGNC=21109|UniProtKB=Q96EK7	FAM120B	Constitutive coactivator of peroxisome proliferator-activated receptor gamma;FAM120B;ortholog
HUMAN|HGNC=26773|UniProtKB=Q52LJ0	FAM98B	Protein FAM98B;FAM98B;ortholog
HUMAN|HGNC=5031|UniProtKB=P09651	HNRNPA1	Heterogeneous nuclear ribonucleoprotein A1;HNRNPA1;ortholog
HUMAN|HGNC=5033|UniProtKB=P22626	HNRNPA2B1	Heterogeneous nuclear ribonucleoproteins A2/B1;HNRNPA2B1;ortholog
HUMAN|HGNC=24941|UniProtKB=P51991	HNRNPA3	Heterogeneous nuclear ribonucleoprotein A3;HNRNPA3;ortholog
HUMAN|HGNC=5330|UniProtKB=P41252	IARS	Isoleucine--tRNA ligase, cytoplasmic;IARS;ortholog
HUMAN|HGNC=6215|UniProtKB=Q15046	KARS	Lysine--tRNA ligase;KARS;ortholog
HUMAN|HGNC=6512|UniProtKB=Q9P2J5	LARS	Leucine--tRNA ligase, cytoplasmic;LARS;ortholog
HUMAN|HGNC=6701|UniProtKB=P30533	LRPAP1	Alpha-2-macroglobulin receptor-associated protein;LRPAP1;ortholog
HUMAN|HGNC=6898|UniProtKB=P56192	MARS	Methionine--tRNA ligase, cytoplasmic;MARS;ortholog
HUMAN|HGNC=7095|UniProtKB=O15344	MID1	E3 ubiquitin-protein ligase Midline-1;MID1;ortholog
HUMAN|HGNC=7804|UniProtKB=P23511	NFYA	Nuclear transcription factor Y subunit alpha;NFYA;ortholog
HUMAN|HGNC=7827|UniProtKB=Q9BPW8	NIPSNAP1	Protein NipSnap homolog 1;NIPSNAP1;ortholog
HUMAN|HGNC=7857|UniProtKB=P30419	NMT1	Glycylpeptide N-tetradecanoyltransferase 1;NMT1;ortholog
HUMAN|HGNC=17946|UniProtKB=Q9Y314	NOSIP	Nitric oxide synthase-interacting protein;NOSIP;ortholog
HUMAN|HGNC=8604|UniProtKB=O95340	PAPSS2	Bifunctional 3'-phosphoadenosine 5'-phosphosulfate synthase 2;PAPSS2;ortholog
HUMAN|HGNC=9077|UniProtKB=P53350	PLK1	Serine/threonine-protein kinase PLK1;PLK1;ortholog
HUMAN|HGNC=9658|UniProtKB=P29350	PTPN6	Tyrosine-protein phosphatase non-receptor type 6;PTPN6;ortholog
HUMAN|HGNC=9751|UniProtKB=P47897	QARS	Glutamine--tRNA ligase;QARS;ortholog
HUMAN|HGNC=9870|UniProtKB=P54136	RARS	Arginine--tRNA ligase, cytoplasmic;RARS;ortholog
HUMAN|HGNC=32310|UniProtKB=Q8IXT5	RBM12B	RNA-binding protein 12B;RBM12B;ortholog
HUMAN|HGNC=17688|UniProtKB=P78346	RPP30	Ribonuclease P protein subunit p30;RPP30;ortholog
HUMAN|HGNC=11303|UniProtKB=O76094	SRP72	Signal recognition particle subunit SRP72;SRP72;ortholog

Supplementary Table 4.2 Human Proteins Found enriched on system 2 relatively to system 1a

Gene ID	Mapped IDs	Gene Name
HUMAN|HGNC=17066|UniProtKB=Q9UKV3	ACIN1	Apoptotic chromatin condensation inducer in the nucleus;ACIN1;ortholog
HUMAN|HGNC=24124|UniProtKB=O96019	ACTL6A	Actin-like protein 6A;ACTL6A;ortholog
HUMAN|HGNC=225|UniProtKB=P55265	ADAR	Double-stranded RNA-specific adenosine deaminase;ADAR;ortholog
HUMAN|HGNC=371|UniProtKB=Q12802	AKAP13	A-kinase anchor protein 13;AKAP13;ortholog
HUMAN|HGNC=24714|UniProtKB=Q8IWZ3	ANKHD1	Ankyrin repeat and KH domain-containing protein 1;ANKHD1;ortholog
HUMAN|HGNC=682|UniProtKB=Q92974	ARHGEF2	Rho guanine nucleotide exchange factor 2;ARHGEF2;ortholog
HUMAN|HGNC=15607|UniProtKB=Q14155	ARHGEF7	Rho guanine nucleotide exchange factor 7;ARHGEF7;ortholog
HUMAN|HGNC=10555|UniProtKB=Q99700	ATXN2	Ataxin-2;ATXN2;ortholog
HUMAN|HGNC=937|UniProtKB=Q99933	BAG1	BAG family molecular chaperone regulator 1;BAG1;ortholog
HUMAN|HGNC=938|UniProtKB=O95816	BAG2	BAG family molecular chaperone regulator 2;BAG2;ortholog
HUMAN|HGNC=13575|UniProtKB=O60885	BRD4	Bromodomain-containing protein 4;BRD4;ortholog
HUMAN|HGNC=1424|UniProtKB=P27708	CAD	CAD protein;CAD;ortholog
HUMAN|HGNC=29188|UniProtKB=Q08AD1	CAMSAP2	Calmodulin-regulated spectrin-associated protein 2;CAMSAP2;ortholog
HUMAN|HGNC=24236|UniProtKB=Q8IX12	CCAR1	Cell division cycle and apoptosis regulator protein 1;CCAR1;ortholog
HUMAN|HGNC=23360|UniProtKB=Q8N163	CCAR2	Cell cycle and apoptosis regulator protein 2;CCAR2;ortholog
HUMAN|HGNC=1728|UniProtKB=P30260	CDC27	Cell division cycle protein 27 homolog;CDC27;ortholog
HUMAN|HGNC=1743|UniProtKB=Q99459	CDC5L	Cell division cycle 5-like protein;CDC5L;ortholog
HUMAN|HGNC=28920|UniProtKB=Q5SW79	CEP170	Centrosomal protein of 170 kDa;CEP170;ortholog
HUMAN|HGNC=1985|UniProtKB=O14578	CIT	Citron Rho-interacting kinase;CIT;ortholog
HUMAN|HGNC=17088|UniProtKB=Q7Z460	CLASP1	CLIP-associating protein 1;CLASP1;ortholog
HUMAN|HGNC=17078|UniProtKB=O75122	CLASP2	CLIP-associating protein 2;CLASP2;ortholog
HUMAN|HGNC=23186|UniProtKB=Q14677	CLINT1	Clathrin interactor 1;CLINT1;ortholog
HUMAN|HGNC=18290|UniProtKB=Q8NFW8	CMAS	N-acylneuraminate cytidylyltransferase;CMAS;ortholog
HUMAN|HGNC=7877|UniProtKB=A5YKK6	CNOT1	CCR4-NOT transcription complex subunit 1;CNOT1;ortholog
HUMAN|HGNC=2230|UniProtKB=P53621	COPA	Coatomer subunit alpha;COPA;ortholog
HUMAN|HGNC=2323|UniProtKB=P31327	CPS1	Carbamoyl-phosphate synthase [ammonia], mitochondrial;CPS1;ortholog
HUMAN|HGNC=2324|UniProtKB=Q10570	CPSF1	Cleavage and polyadenylation specificity factor subunit 1;CPSF1;ortholog
HUMAN|HGNC=2325|UniProtKB=Q9P2I0	CPSF2	Cleavage and polyadenylation specificity factor subunit 2;CPSF2;ortholog
HUMAN|HGNC=2327|UniProtKB=O95639	CPSF4	Cleavage and polyadenylation specificity factor subunit 4;CPSF4;ortholog
HUMAN|HGNC=15762|UniProtKB=Q9BZJ0	CRNKL1	Crooked neck-like protein 1;CRNKL1;ortholog
HUMAN|HGNC=29322|UniProtKB=Q9HCG8	CWC22	Pre-mRNA-splicing factor CWC22 homolog;CWC22;ortholog
HUMAN|HGNC=13759|UniProtKB=Q7L576	CYFIP1	Cytoplasmic FMR1-interacting protein 1;CYFIP1;ortholog
HUMAN|HGNC=30915|UniProtKB=P61962	DCAF7	DDB1- and CUL4-associated factor 7;DCAF7;ortholog
HUMAN|HGNC=2743|UniProtKB=Q9UHI6	DDX20	Probable ATP-dependent RNA helicase DDX20;DDX20;ortholog
HUMAN|HGNC=2745|UniProtKB=O00571	DDX3X	ATP-dependent RNA helicase DDX3X;DDX3X;ortholog
HUMAN|HGNC=15815|UniProtKB=Q7Z478	DHX29	ATP-dependent RNA helicase DHX29;DHX29;ortholog
HUMAN|HGNC=16716|UniProtKB=Q7L2E3	DHX30	Putative ATP-dependent RNA helicase DHX30;DHX30;ortholog
HUMAN|HGNC=17211|UniProtKB=Q92620	DHX38	Pre-mRNA-splicing factor ATP-dependent RNA helicase PRP16;DHX38;ortholog
HUMAN|HGNC=5229|UniProtKB=P31689	DNAJA1	DnaJ homolog subfamily A member 1;DNAJA1;ortholog
HUMAN|HGNC=14884|UniProtKB=O60884	DNAJA2	DnaJ homolog subfamily A member 2;DNAJA2;ortholog
HUMAN|HGNC=14889|UniProtKB=Q9UBS4	DNAJB11	DnaJ homolog subfamily B member 11;DNAJB11;ortholog
HUMAN|HGNC=30343|UniProtKB=O75165	DNAJC13	DnaJ homolog subfamily C member 13;DNAJC13;ortholog
HUMAN|HGNC=2976|UniProtKB=P26358	DNMT1	DNA (cytosine-5)-methyltransferase 1;DNMT1;ortholog
HUMAN|HGNC=16160|UniProtKB=Q9H147	DNTTIP1	Deoxynucleotidyltransferase terminal-interacting protein 1;DNTTIP1;ortholog
HUMAN|HGNC=23476|UniProtKB=Q9H7D0	DOCK5	Dedicator of cytokinesis protein 5;DOCK5;ortholog
HUMAN|HGNC=2961|UniProtKB=Q14204	DYNC1H1	Cytoplasmic dynein 1 heavy chain 1;DYNC1H1;ortholog
HUMAN|HGNC=17157|UniProtKB=Q6P2E9	EDC4	Enhancer of mRNA-decapping protein 4;EDC4;ortholog
HUMAN|HGNC=3296|UniProtKB=Q04637	EIF4G1	Eukaryotic translation initiation factor 4 gamma 1;EIF4G1;ortholog
HUMAN|HGNC=17233|UniProtKB=Q96JJ3	ELMO2	Engulfment and cell motility protein 2;ELMO2;ortholog
HUMAN|HGNC=18035|UniProtKB=O95834	EML2	Echinoderm microtubule-associated protein-like 2;EML2;ortholog
HUMAN|HGNC=26666|UniProtKB=Q32P44	EML3	Echinoderm microtubule-associated protein-like 3;EML3;ortholog
HUMAN|HGNC=17072|UniProtKB=Q8IUD2	ERC1	ELKS/Rab6-interacting/CAST family member 1;ERC1;ortholog
HUMAN|HGNC=24968|UniProtKB=Q96KP1	EXOC2	Exocyst complex component 2;EXOC2;ortholog
HUMAN|HGNC=13247|UniProtKB=Q9NZB2	FAM120A	Constitutive coactivator of PPAR-gamma-like protein 1;FAM120A;ortholog
HUMAN|HGNC=17905|UniProtKB=Q9Y613	FHOD1	FH1/FH2 domain-containing protein 1;FHOD1;ortholog
HUMAN|HGNC=3754|UniProtKB=P21333	FLNA	Filamin-A;FLNA;ortholog
HUMAN|HGNC=19752|UniProtKB=Q8N3X1	FNBP4	Formin-binding protein 4;FNBP4;ortholog
HUMAN|HGNC=4004|UniProtKB=Q96AE4	FUBP1	Far upstream element-binding protein 1;FUBP1;ortholog
HUMAN|HGNC=4005|UniProtKB=Q96I24	FUBP3	Far upstream element-binding protein 3;FUBP3;ortholog
HUMAN|HGNC=9126|UniProtKB=O75420	GIGYF1	GRB10-interacting GYF protein 1;GIGYF1;ortholog
HUMAN|HGNC=4272|UniProtKB=Q9Y2X7	GIT1	ARF GTPase-activating protein GIT1;GIT1;ortholog
HUMAN|HGNC=4426|UniProtKB=Q08378	GOLGA3	Golgin subfamily A member 3;GOLGA3;ortholog
HUMAN|HGNC=4655|UniProtKB=P32780	GTF2H1	General transcription factor IIH subunit 1;GTF2H1;ortholog
HUMAN|HGNC=31394|UniProtKB=Q6P1K8	GTF2H2C	General transcription factor IIH subunit 2-like protein;GTF2H2C;ortholog
HUMAN|HGNC=4658|UniProtKB=Q92759	GTF2H4	General transcription factor IIH subunit 4;GTF2H4;ortholog
HUMAN|HGNC=4839|UniProtKB=P51610	HCFC1	Host cell factor 1;HCFC1;ortholog
HUMAN|HGNC=4853|UniProtKB=Q92769	HDAC2	Histone deacetylase 2;HDAC2;ortholog
HUMAN|HGNC=5043|UniProtKB=P31942	HNRNPH3	Heterogeneous nuclear ribonucleoprotein H3;HNRNPH3;ortholog
HUMAN|HGNC=17853|UniProtKB=Q9P2D0	IBTK	Inhibitor of Bruton tyrosine kinase;IBTK;ortholog
HUMAN|HGNC=5395|UniProtKB=Q16666	IFI16	Gamma-interferon-inducible protein 16;IFI16;ortholog
HUMAN|HGNC=5959|UniProtKB=O95163	IKBKAP	Elongator complex protein 1;ELP1;ortholog
HUMAN|HGNC=29300|UniProtKB=Q63ZY3	KANK2	KN motif and ankyrin repeat domain-containing protein 2;KANK2;ortholog
HUMAN|HGNC=18116|UniProtKB=Q07666	KHDRBS1	KH domain-containing, RNA-binding, signal transduction-associated protein 1;KHDRBS1;ortholog
HUMAN|HGNC=6316|UniProtKB=Q92945	KHSRP	Far upstream element-binding protein 2;KHSRP;ortholog
HUMAN|HGNC=24500|UniProtKB=Q69YN4	KIAA1429	Protein virilizer homolog VIRMAortholog
HUMAN|HGNC=29301|UniProtKB=Q9P206	KIAA1522	Uncharacterized protein KIAA1522;KIAA1522;ortholog
HUMAN|HGNC=6392|UniProtKB=Q02241	KIF23	Kinesin-like protein KIF23;KIF23;ortholog
HUMAN|HGNC=6318|UniProtKB=O00139	KIF2A	Kinesin-like protein KIF2A;KIF2A;ortholog
HUMAN|HGNC=6393|UniProtKB=Q99661	KIF2C	Kinesin-like protein KIF2C;KIF2C;ortholog
HUMAN|HGNC=13339|UniProtKB=O95239	KIF4A	Chromosome-associated kinesin KIF4A;KIF4A;ortholog
HUMAN|HGNC=6490|UniProtKB=Q13751	LAMB3	Laminin subunit beta-3;LAMB3;ortholog
HUMAN|HGNC=29531|UniProtKB=Q6PKG0	LARP1	La-related protein 1;LARP1;ortholog
HUMAN|HGNC=24320|UniProtKB=Q71RC2	LARP4	La-related protein 4;LARP4;ortholog
HUMAN|HGNC=28987|UniProtKB=Q92615	LARP4B	La-related protein 4B;LARP4B;ortholog
HUMAN|HGNC=15714|UniProtKB=P42704	LRPPRC	Leucine-rich PPR motif-containing protein, mitochondrial;LRPPRC;ortholog
HUMAN|HGNC=21581|UniProtKB=Q5VZK9	LRRC16A	F-actin-uncapping protein LRRC16A;CARMIL1;ortholog
HUMAN|HGNC=24309|UniProtKB=O95232	LUC7L3	Luc7-like protein 3;LUC7L3;ortholog
HUMAN|HGNC=18752|UniProtKB=Q9BPZ7	MAPKAP1	Target of rapamycin complex 2 subunit MAPKAP1;MAPKAP1;ortholog
HUMAN|HGNC=3332|UniProtKB=Q7KZI7	MARK2	Serine/threonine-protein kinase MARK2;MARK2;ortholog
HUMAN|HGNC=6897|UniProtKB=P27448	MARK3	MAP/microtubule affinity-regulating kinase 3;MARK3;ortholog
HUMAN|HGNC=6912|UniProtKB=P43243	MATR3	Matrin-3;MATR3;ortholog
HUMAN|HGNC=21163|UniProtKB=Q14676	MDC1	Mediator of DNA damage checkpoint protein 1;MDC1;ortholog
HUMAN|HGNC=2370|UniProtKB=O60244	MED14	Mediator of RNA polymerase II transcription subunit 14;MED14;ortholog
HUMAN|HGNC=11473|UniProtKB=Q13503	MED21	Mediator of RNA polymerase II transcription subunit 21;MED21;ortholog
HUMAN|HGNC=22963|UniProtKB=O75448	MED24	Mediator of RNA polymerase II transcription subunit 24;MED24;ortholog
HUMAN|HGNC=20247|UniProtKB=Q7L2J0	MEPCE	7SK snRNA methylphosphate capping enzyme;MEPCE;ortholog
HUMAN|HGNC=21905|UniProtKB=Q9NXC5	MIOS	GATOR complex protein MIOS;MIOS;ortholog
HUMAN|HGNC=7107|UniProtKB=P46013	MKI67	Proliferation marker protein Ki-67;MKI67;ortholog
HUMAN|HGNC=7230|UniProtKB=P49959	MRE11A	Double-strand break repair protein MRE11;MRE11;ortholog
HUMAN|HGNC=7449|UniProtKB=Q13613	MTMR1	Myotubularin-related protein 1;MTMR1;ortholog
HUMAN|HGNC=7546|UniProtKB=Q9BQG0	MYBBP1A	Myb-binding protein 1A;MYBBP1A;ortholog
HUMAN|HGNC=23386|UniProtKB=O75592	MYCBP2	E3 ubiquitin-protein ligase MYCBP2;MYCBP2;ortholog
HUMAN|HGNC=7609|UniProtKB=Q13459	MYO9B	Unconventional myosin-IXb;MYO9B;ortholog
HUMAN|HGNC=23246|UniProtKB=Q86TC9	MYPN	Myopalladin;MYPN;ortholog
HUMAN|HGNC=7652|UniProtKB=O60934	NBN	Nibrin;NBN;ortholog
HUMAN|HGNC=7666|UniProtKB=Q9Y2A7	NCKAP1	Nck-associated protein 1;NCKAP1;ortholog
HUMAN|HGNC=7765|UniProtKB=P21359	NF1	Neurofibromin;NF1;ortholog
HUMAN|HGNC=7785|UniProtKB=O00712	NFIB	Nuclear factor 1 B-type;NFIB;ortholog
HUMAN|HGNC=7978|UniProtKB=P04150	NR3C1	Glucocorticoid receptor;NR3C1;ortholog
HUMAN|HGNC=25994|UniProtKB=Q08J23	NSUN2	tRNA (cytosine(34)-C(5))-methyltransferase;NSUN2;ortholog
HUMAN|HGNC=8060|UniProtKB=P49757	NUMB	Protein numb homolog;NUMB;ortholog
HUMAN|HGNC=8127|UniProtKB=O15294	OGT	UDP-N-acetylglucosamine--peptide N-acetylglucosaminyltransferase 110 kDa subunit;OGT;ortholog
HUMAN|HGNC=24949|UniProtKB=Q01804	OTUD4	OTU domain-containing protein 4;OTUD4;ortholog
HUMAN|HGNC=8546|UniProtKB=P13674	P4HA1	Prolyl 4-hydroxylase subunit alpha-1;P4HA1;ortholog
HUMAN|HGNC=17068|UniProtKB=Q8WX93	PALLD	Palladin;PALLD;ortholog
HUMAN|HGNC=29232|UniProtKB=Q460N5	PARP14	Poly [ADP-ribose] polymerase 14;PARP14;ortholog
HUMAN|HGNC=13579|UniProtKB=Q9Y5B6	PAXBP1	PAX3- and PAX7-binding protein 1;PAXBP1;ortholog
HUMAN|HGNC=8921|UniProtKB=Q92576	PHF3	PHD finger protein 3;PHF3;ortholog
HUMAN|HGNC=9162|UniProtKB=Q9H307	PNN	Pinin;PNN;ortholog
HUMAN|HGNC=18801|UniProtKB=Q7Z3K3	POGZ	Pogo transposable element with ZNF domain;POGZ;ortholog
HUMAN|HGNC=9188|UniProtKB=P30876	POLR2B	DNA-directed RNA polymerase II subunit RPB2;POLR2B;ortholog
HUMAN|HGNC=9245|UniProtKB=Q13136	PPFIA1	Liprin-alpha-1;PPFIA1;ortholog
HUMAN|HGNC=9249|UniProtKB=Q86W92	PPFIBP1	Liprin-beta-1;PPFIBP1;ortholog
HUMAN|HGNC=9273|UniProtKB=O60437	PPL	Periplakin;PPL;ortholog
HUMAN|HGNC=9281|UniProtKB=P62136	PPP1CA	Serine/threonine-protein phosphatase PP1-alpha catalytic subunit;PPP1CA;ortholog
HUMAN|HGNC=9284|UniProtKB=Q96QC0	PPP1R10	Serine/threonine-protein phosphatase 1 regulatory subunit 10;PPP1R10;ortholog
HUMAN|HGNC=9302|UniProtKB=P30153	PPP2R1A	Serine/threonine-protein phosphatase 2A 65 kDa regulatory subunit A alpha isoform;PPP2R1A;ortholog
HUMAN|HGNC=16463|UniProtKB=O75400	PRPF40A	Pre-mRNA-processing factor 40 homolog A;PRPF40A;ortholog
HUMAN|HGNC=17340|UniProtKB=Q6P2Q9	PRPF8	Pre-mRNA-processing-splicing factor 8;PRPF8;ortholog
HUMAN|HGNC=13918|UniProtKB=P48634	PRRC2A	Protein PRRC2A;PRRC2A;ortholog
HUMAN|HGNC=24903|UniProtKB=Q9Y520	PRRC2C	Protein PRRC2C;PRRC2C;ortholog
HUMAN|HGNC=14957|UniProtKB=Q14671	PUM1	Pumilio homolog 1;PUM1;ortholog
HUMAN|HGNC=24845|UniProtKB=Q9BXF6	RAB11FIP5	Rab11 family-interacting protein 5;RAB11FIP5;ortholog
HUMAN|HGNC=9804|UniProtKB=Q9H0H5	RACGAP1	Rac GTPase-activating protein 1;RACGAP1;ortholog
HUMAN|HGNC=9816|UniProtKB=Q92878	RAD50	DNA repair protein RAD50;RAD50;ortholog
HUMAN|HGNC=17770|UniProtKB=Q6GYQ0	RALGAPA1	Ral GTPase-activating protein subunit alpha-1;RALGAPA1;ortholog
HUMAN|HGNC=29221|UniProtKB=Q86X10	RALGAPB	Ral GTPase-activating protein subunit beta;RALGAPB;ortholog
HUMAN|HGNC=9848|UniProtKB=P49792	RANBP2	E3 SUMO-protein ligase RanBP2;RANBP2;ortholog
HUMAN|HGNC=9896|UniProtKB=P98175	RBM10	RNA-binding protein 10;RBM10;ortholog
HUMAN|HGNC=14959|UniProtKB=Q96T37	RBM15	Putative RNA-binding protein 15;RBM15;ortholog
HUMAN|HGNC=29243|UniProtKB=Q9P2N5	RBM27	RNA-binding protein 27;RBM27;ortholog
HUMAN|HGNC=27223|UniProtKB=Q96EV2	RBM33	RNA-binding protein 33;RBM33;ortholog
HUMAN|HGNC=30358|UniProtKB=A0AV96	RBM47	RNA-binding protein 47;RBM47;ortholog
HUMAN|HGNC=28611|UniProtKB=Q6R327	RICTOR	Rapamycin-insensitive companion of mTOR;RICTOR;ortholog
HUMAN|HGNC=23207|UniProtKB=Q5UIP0	RIF1	Telomere-associated protein RIF1;RIF1;ortholog
HUMAN|HGNC=25671|UniProtKB=Q5TBB1	RNASEH2B	Ribonuclease H2 subunit B;RNASEH2B;ortholog
HUMAN|HGNC=10080|UniProtKB=Q15287	RNPS1	RNA-binding protein with serine-rich domain 1;RNPS1;ortholog
HUMAN|HGNC=10474|UniProtKB=Q9Y265	RUVBL1	RuvB-like 1;RUVBL1;ortholog
HUMAN|HGNC=10538|UniProtKB=O43290	SART1	U4/U6.U5 tri-snRNP-associated protein 1;SART1;ortholog
HUMAN|HGNC=10542|UniProtKB=O95248	SBF1	Myotubularin-related protein 5;SBF1;ortholog
HUMAN|HGNC=10697|UniProtKB=P55735	SEC13	Protein SEC13 homolog;SEC13;ortholog
HUMAN|HGNC=29006|UniProtKB=O15027	SEC16A	Protein transport protein Sec16A;SEC16A;ortholog
HUMAN|HGNC=30379|UniProtKB=Q96EE3	SEH1L	Nucleoporin SEH1;SEH1L;ortholog
HUMAN|HGNC=17860|UniProtKB=Q8NC51	SERBP1	Plasminogen activator inhibitor 1 RNA-binding protein;SERBP1;ortholog
HUMAN|HGNC=10766|UniProtKB=Q15428	SF3A2	Splicing factor 3A subunit 2;SF3A2;ortholog
HUMAN|HGNC=29547|UniProtKB=Q8NEM2	SHCBP1	SHC SH2 domain-binding protein 1;SHCBP1;ortholog
HUMAN|HGNC=10852|UniProtKB=P34897	SHMT2	Serine hydroxymethyltransferase, mitochondrial;SHMT2;ortholog
HUMAN|HGNC=11100|UniProtKB=P51532	SMARCA4	Transcription activator BRG1;SMARCA4;ortholog
HUMAN|HGNC=11103|UniProtKB=Q12824	SMARCB1	SWI/SNF-related matrix-associated actin-dependent regulator of chromatin subfamily B member 1;SMARCB1;ortholog
HUMAN|HGNC=11104|UniProtKB=Q92922	SMARCC1	SWI/SNF complex subunit SMARCC1;SMARCC1;ortholog
HUMAN|HGNC=11105|UniProtKB=Q8TAQ2	SMARCC2	SWI/SNF complex subunit SMARCC2;SMARCC2;ortholog
HUMAN|HGNC=18247|UniProtKB=Q2TAY7	SMU1	WD40 repeat-containing protein SMU1;SMU1;ortholog
HUMAN|HGNC=30859|UniProtKB=O75643	SNRNP200	U5 small nuclear ribonucleoprotein 200 kDa helicase;SNRNP200;ortholog
HUMAN|HGNC=11158|UniProtKB=P62314	SNRPD1	Small nuclear ribonucleoprotein Sm D1;SNRPD1;ortholog
HUMAN|HGNC=20073|UniProtKB=Q96L92	SNX27	Sorting nexin-27;SNX27;ortholog
HUMAN|HGNC=11183|UniProtKB=P18583	SON	Protein SON;SON;ortholog
HUMAN|HGNC=19744|UniProtKB=O43295	SRGAP2	SLIT-ROBO Rho GTPase-activating protein 3;SRGAP3;ortholog
HUMAN|HGNC=16638|UniProtKB=Q8IYB3	SRRM1	Serine/arginine repetitive matrix protein 1;SRRM1;ortholog
HUMAN|HGNC=16639|UniProtKB=Q9UQ35	SRRM2	Serine/arginine repetitive matrix protein 2;SRRM2;ortholog
HUMAN|HGNC=10782|UniProtKB=Q05519	SRSF11	Serine/arginine-rich splicing factor 11;SRSF11;ortholog
HUMAN|HGNC=10788|UniProtKB=Q13247	SRSF6	Serine/arginine-rich splicing factor 6;SRSF6;ortholog
HUMAN|HGNC=25824|UniProtKB=Q6ZW31	SYDE1	Rho GTPase-activating protein SYDE1;SYDE1;ortholog
HUMAN|HGNC=22935|UniProtKB=Q92797	SYMPK	Symplekin;SYMPK;ortholog
HUMAN|HGNC=16918|UniProtKB=O60506	SYNCRIP	Heterogeneous nuclear ribonucleoprotein Q;SYNCRIP;ortholog
HUMAN|HGNC=29364|UniProtKB=Q9C0D5	TANC1	Protein TANC1;TANC1;ortholog
HUMAN|HGNC=19165|UniProtKB=O60343	TBC1D4	TBC1 domain family member 4;TBC1D4;ortholog
HUMAN|HGNC=29529|UniProtKB=Q9BZK7	TBL1XR1	F-box-like/WD repeat-containing protein TBL1XR1;TBL1XR1;ortholog
HUMAN|HGNC=17165|UniProtKB=Q9UBB9	TFIP11	Tuftelin-interacting protein 11;TFIP11;ortholog
HUMAN|HGNC=11827|UniProtKB=Q07157	TJP1	Tight junction protein ZO-1;TJP1;ortholog
HUMAN|HGNC=11828|UniProtKB=Q9UDY2	TJP2	Tight junction protein ZO-2;TJP2;ortholog
HUMAN|HGNC=19081|UniProtKB=Q9C0C2	TNKS1BP1	182 kDa tankyrase-1-binding protein;TNKS1BP1;ortholog
HUMAN|HGNC=17008|UniProtKB=Q92547	TOPBP1	DNA topoisomerase 2-binding protein 1;TOPBP1;ortholog
HUMAN|HGNC=20316|UniProtKB=Q86TN4	TRPT1	tRNA 2'-phosphotransferase 1;TRPT1;ortholog
HUMAN|HGNC=12347|UniProtKB=Q9Y4A5	TRRAP	Transformation/transcription domain-associated protein;TRRAP;ortholog
HUMAN|HGNC=12407|UniProtKB=P68366	TUBA4A	Tubulin alpha-4A chain;TUBA4A;ortholog
HUMAN|HGNC=20776|UniProtKB=Q9BUF5	TUBB6	Tubulin beta-6 chain;TUBB6;ortholog
HUMAN|HGNC=20773|UniProtKB=Q3ZCM7	TUBB8	Tubulin beta-8 chain;TUBB8;ortholog
HUMAN|HGNC=12417|UniProtKB=P23258	TUBG1	Tubulin gamma-1 chain;TUBG1;ortholog
HUMAN|HGNC=18599|UniProtKB=Q9BSJ2	TUBGCP2	Gamma-tubulin complex component 2;TUBGCP2;ortholog
HUMAN|HGNC=18598|UniProtKB=Q96CW5	TUBGCP3	Gamma-tubulin complex component 3;TUBGCP3;ortholog
HUMAN|HGNC=16806|UniProtKB=O95071	UBR5	E3 ubiquitin-protein ligase UBR5;UBR5;ortholog
HUMAN|HGNC=17854|UniProtKB=Q9HAU5	UPF2	Regulator of nonsense transcripts 2;UPF2;ortholog
HUMAN|HGNC=20852|UniProtKB=Q96S15	WDR24	GATOR complex protein WDR24;WDR24;ortholog
HUMAN|HGNC=25651|UniProtKB=Q9C0J8	WDR33	pre-mRNA 3' end processing protein WDR33;WDR33;ortholog
HUMAN|HGNC=12757|UniProtKB=P61964	WDR5	WD repeat-containing protein 5;WDR5;ortholog
HUMAN|HGNC=28826|UniProtKB=Q6UXN9	WDR82	WD repeat-containing protein 82;WDR82;ortholog
HUMAN|HGNC=14540|UniProtKB=Q9H4A3	WNK1	Serine/threonine-protein kinase WNK1;WNK1;ortholog
HUMAN|HGNC=14089|UniProtKB=Q9HCS7	XAB2	Pre-mRNA-splicing factor SYF1;XAB2;ortholog
HUMAN|HGNC=25489|UniProtKB=Q9ULM3	YEATS2	YEATS domain-containing protein 2;YEATS2;ortholog
HUMAN|HGNC=12856|UniProtKB=P25490	YY1	Transcriptional repressor protein YY1;YY1;ortholog
HUMAN|HGNC=29093|UniProtKB=O75152	ZC3H11A	Zinc finger CCCH domain-containing protein 11A;ZC3H11A;ortholog
HUMAN|HGNC=20368|UniProtKB=Q5T200	ZC3H13	Zinc finger CCCH domain-containing protein 13;ZC3H13;ortholog
HUMAN|HGNC=17277|UniProtKB=Q96KR1	ZFR	Zinc finger RNA-binding protein;ZFR;ortholog
HUMAN|HGNC=28079|UniProtKB=Q86UK7	ZNF598	E3 ubiquitin-protein ligase ZNF598;ZNF598;ortholog

Supplementary Table 4.3 Human proteins Common to system 1a and 2		
Gene ID	Mapped IDs	Gene Name
HUMAN|HG	ALKBH5	RNA demethylase ALKBH5;ALKBH5;ortholog
HUMAN|HG	ANLN	Anillin;ANLN;ortholog
HUMAN|HG	AP3D1	AP-3 complex subunit delta-1;AP3D1;ortholog
HUMAN|HG	API5	Apoptosis inhibitor 5;API5;ortholog
HUMAN|HG	ARAF	Serine/threonine-protein kinase A-Raf;ARAF;ortholog
HUMAN|HG	ATXN2L	Ataxin-2-like protein;ATXN2L;ortholog
HUMAN|HG	BCAR1	Breast cancer anti-estrogen resistance protein 1;BCAR1;ortholog
HUMAN|HG	BUB3	Mitotic checkpoint protein BUB3;BUB3;ortholog
HUMAN|HG	c14orf66	Sphingolipid delta(4)-desaturase/C4-monooxygenase DES2;DEGS2;ortholog
HUMAN|HG	c14orf80	Tubulin epsilon and delta complex protein 1;TEDC1;ortholog
HUMAN|HG	CAMK2D	Calcium/calmodulin-dependent protein kinase type II subunit delta;CAMK2D;ortholog
HUMAN|HG	CAMK2G	Calcium/calmodulin-dependent protein kinase type II subunit gamma;CAMK2G;ortholog
HUMAN|HG	CC2D1A	Coiled-coil and C2 domain-containing protein 1A;CC2D1A;ortholog
HUMAN|HG	CCDC50	Coiled-coil domain-containing protein 50;CCDC50;ortholog
HUMAN|HG	CDC73	Parafibromin;CDC73;ortholog
HUMAN|HG	CDK2	Cyclin-dependent kinase 2;CDK2;ortholog
HUMAN|HG	CDK7	Cyclin-dependent kinase 7;CDK7;ortholog
HUMAN|HG	CHTOP	Chromatin target of PRMT1 protein;CHTOP;ortholog
HUMAN|HG	CNBP	Cellular nucleic acid-binding protein;CNBP;ortholog
HUMAN|HG	CNOT10	CCR4-NOT transcription complex subunit 10;CNOT10;ortholog
HUMAN|HG	CNOT7	CCR4-NOT transcription complex subunit 7;CNOT7;ortholog
HUMAN|HG	COPB2	Coatomer subunit beta';COPB2;ortholog
HUMAN|HG	CPSF3	Cleavage and polyadenylation specificity factor subunit 3;CPSF3;ortholog
HUMAN|HG	CSNK1A1	Casein kinase I isoform alpha;CSNK1A1;ortholog
HUMAN|HG	CSTF1	Cleavage stimulation factor subunit 1;CSTF1;ortholog
HUMAN|HG	CSTF3	Cleavage stimulation factor subunit 3;CSTF3;ortholog
HUMAN|HG	CWC27	Peptidyl-prolyl cis-trans isomerase CWC27 homolog;CWC27;ortholog
HUMAN|HG	DBR1	Lariat debranching enzyme;DBR1;ortholog
HUMAN|HG	DDX1	ATP-dependent RNA helicase DDX1;DDX1;ortholog
HUMAN|HG	DDX23	Probable ATP-dependent RNA helicase DDX23;DDX23;ortholog
HUMAN|HG	DDX41	Probable ATP-dependent RNA helicase DDX41;DDX41;ortholog
HUMAN|HG	DDX42	ATP-dependent RNA helicase DDX42;DDX42;ortholog
HUMAN|HG	DDX52	Probable ATP-dependent RNA helicase DDX52;DDX52;ortholog
HUMAN|HG	DDX6	Probable ATP-dependent RNA helicase DDX6;DDX6;ortholog
HUMAN|HG	DHX16	Pre-mRNA-splicing factor ATP-dependent RNA helicase DHX16;DHX16;ortholog
HUMAN|HG	DHX36	ATP-dependent RNA helicase DHX36;DHX36;ortholog
HUMAN|HG	DHX40	Probable ATP-dependent RNA helicase DHX40;DHX40;ortholog
HUMAN|HG	DIAPH3	Protein diaphanous homolog 3;DIAPH3;ortholog
HUMAN|HG	DYNC1LI1	Cytoplasmic dynein 1 light intermediate chain 1;DYNC1LI1;ortholog
HUMAN|HG	DYNC1LI2	Cytoplasmic dynein 1 light intermediate chain 2;DYNC1LI2;ortholog
HUMAN|HG	EFTUD2	116 kDa U5 small nuclear ribonucleoprotein component;EFTUD2;ortholog
HUMAN|HG	EIF2S2	Eukaryotic translation initiation factor 2 subunit 2;EIF2S2;ortholog
HUMAN|HG	EIF4E2	Eukaryotic translation initiation factor 4E type 2;EIF4E2;ortholog
HUMAN|HG	ELP3	Elongator complex protein 3;ELP3;ortholog
HUMAN|HG	EWSR1	RNA-binding protein EWS;EWSR1;ortholog
HUMAN|HG	EXOC1	Exocyst complex component 1;EXOC1;ortholog
HUMAN|HG	EXOC4	Exocyst complex component 4;EXOC4;ortholog
HUMAN|HG	EXOSC2	Exosome complex component RRP4;EXOSC2;ortholog
HUMAN|HG	EXOSC4	Exosome complex component RRP41;EXOSC4;ortholog
HUMAN|HG	FAM129B	Niban-like protein 1;FAM129B;ortholog
HUMAN|HG	FAM208A	Protein TASOR;FAM208A;ortholog
HUMAN|HG	FAM83A	Protein FAM83A;FAM83A;ortholog
HUMAN|HG	FAM83G	Protein FAM83G;FAM83G;ortholog
HUMAN|HG	FAM98A	Protein FAM98A;FAM98A;ortholog
HUMAN|HG	FBXO22	F-box only protein 22;FBXO22;ortholog
HUMAN|HG	FGF2	Fibroblast growth factor 2;FGF2;ortholog
HUMAN|HG	FUS	RNA-binding protein FUS;FUS;ortholog
HUMAN|HG	G3BP1	Ras GTPase-activating protein-binding protein 1;G3BP1;ortholog
HUMAN|HG	G3BP2	Ras GTPase-activating protein-binding protein 2;G3BP2;ortholog
HUMAN|HG	GIGYF2	GRB10-interacting GYF protein 2;GIGYF2;ortholog
HUMAN|HG	GPKOW	G-patch domain and KOW motifs-containing protein;GPKOW;ortholog
HUMAN|HG	GTF2I	General transcription factor II-I;GTF2I;ortholog
HUMAN|HG	HNRNPF	Heterogeneous nuclear ribonucleoprotein F;HNRNPF;ortholog
HUMAN|HG	HNRNPH1	Heterogeneous nuclear ribonucleoprotein H;HNRNPH1;ortholog
HUMAN|HG	HNRNPL	Heterogeneous nuclear ribonucleoprotein L;HNRNPL;ortholog
HUMAN|HG	HNRNPR	Heterogeneous nuclear ribonucleoprotein R;HNRNPR;ortholog
HUMAN|HG	HSPA1B	Heat shock 70 kDa protein 1B;HSPA1B;ortholog
HUMAN|HG	HSPA9	Stress-70 protein, mitochondrial;HSPA9;ortholog
HUMAN|HG	IGF2BP1	Insulin-like growth factor 2 mRNA-binding protein 1;IGF2BP1;ortholog
HUMAN|HG	IK	Protein Red;IK;ortholog
HUMAN|HG	ILF2	Interleukin enhancer-binding factor 2;ILF2;ortholog
HUMAN|HG	ILF3	Interleukin enhancer-binding factor 3;ILF3;ortholog
HUMAN|HG	IPO5	Importin-5;IPO5;ortholog
HUMAN|HG	IRS2	Insulin receptor substrate 2;IRS2;ortholog
HUMAN|HG	KAT7	Histone acetyltransferase KAT7;KAT7;ortholog
HUMAN|HG	KIF5B	Kinesin-1 heavy chain;KIF5B;ortholog
HUMAN|HG	KLHDC4	Kelch domain-containing protein 4;KLHDC4;ortholog
HUMAN|HG	LSM12	Protein LSM12 homolog;LSM12;ortholog
HUMAN|HG	LSM14A	Protein LSM14 homolog A;LSM14A;ortholog
HUMAN|HG	MACF1	Microtubule-actin cross-linking factor 1, isoforms 1/2/3/5;MACF1;ortholog
HUMAN|HG	MAP7D1	MAP7 domain-containing protein 1;MAP7D1;ortholog
HUMAN|HG	MCM3	DNA replication licensing factor MCM3;MCM3;ortholog
HUMAN|HG	MCM5	DNA replication licensing factor MCM5;MCM5;ortholog
HUMAN|HG	MED1	Mediator of RNA polymerase II transcription subunit 1;MED1;ortholog
HUMAN|HG	MOCOS	Molybdenum cofactor sulfurase;MOCOS;ortholog
HUMAN|HG	MOV10	Putative helicase MOV-10;MOV10;ortholog
HUMAN|HG	MSI2	RNA-binding protein Musashi homolog 2;MSI2;ortholog
HUMAN|HG	NCAPD2	Condensin complex subunit 1;NCAPD2;ortholog
HUMAN|HG	NCBP1	Nuclear cap-binding protein subunit 1;NCBP1;ortholog
HUMAN|HG	NCOA5	Nuclear receptor coactivator 5;NCOA5;ortholog
HUMAN|HG	NEDD1	Protein NEDD1;NEDD1;ortholog
HUMAN|HG	NELFA	Negative elongation factor A;NELFA;ortholog
HUMAN|HG	NELFB	Negative elongation factor B;NELFB;ortholog
HUMAN|HG	NELFC/D	Negative elongation factor C/D;NELFCD;ortholog
HUMAN|HG	NELFE	Negative elongation factor E;NELFE;ortholog
HUMAN|HG	NOC4L	Nucleolar complex protein 4 homolog;NOC4L;ortholog
HUMAN|HG	NSUN5	Probable 28S rRNA (cytosine-C(5))-methyltransferase;NSUN5;ortholog
HUMAN|HG	NUFIP2	Nuclear fragile X mental retardation-interacting protein 2;NUFIP2;ortholog
HUMAN|HG	NXF1	Nuclear RNA export factor 1;NXF1;ortholog
HUMAN|HG	P4HB	Protein disulfide-isomerase;P4HB;ortholog
HUMAN|HG	PABPC1	Polyadenylate-binding protein 1;PABPC1;ortholog
HUMAN|HG	PABPC4	Polyadenylate-binding protein 4;PABPC4;ortholog
HUMAN|HG	PABPN1	Polyadenylate-binding protein 2;PABPN1;ortholog
HUMAN|HG	PAK4	Serine/threonine-protein kinase PAK 4;PAK4;ortholog
HUMAN|HG	PARD3	Partitioning defective 3 homolog;PARD3;ortholog
HUMAN|HG	PARG	Poly(ADP-ribose) glycohydrolase;PARG;ortholog
HUMAN|HG	PAWR	PRKC apoptosis WT1 regulator protein;PAWR;ortholog
HUMAN|HG	PCCA	Propionyl-CoA carboxylase alpha chain, mitochondrial;PCCA;ortholog
HUMAN|HG	PCCB	Propionyl-CoA carboxylase beta chain, mitochondrial;PCCB;ortholog
HUMAN|HG	PDE3A	cGMP-inhibited 3',5'-cyclic phosphodiesterase A;PDE3A;ortholog
HUMAN|HG	PFKFB2	6-phosphofructo-2-kinase/fructose-2,6-bisphosphatase 2;PFKFB2;ortholog
HUMAN|HG	PHF5A	PHD finger-like domain-containing protein 5A;PHF5A;ortholog
HUMAN|HG	PKP3	Plakophilin-3;PKP3;ortholog
HUMAN|HG	PLRG1	Pleiotropic regulator 1;PLRG1;ortholog
HUMAN|HG	POLD1	DNA polymerase delta catalytic subunit;POLD1;ortholog
HUMAN|HG	POLD2	DNA polymerase delta subunit 2;POLD2;ortholog
HUMAN|HG	PPIH	Peptidyl-prolyl cis-trans isomerase H;PPIH;ortholog
HUMAN|HG	PPP1R12A	Protein phosphatase 1 regulatory subunit 12A;PPP1R12A;ortholog
HUMAN|HG	PPP2CA	Serine/threonine-protein phosphatase 2A catalytic subunit alpha isoform;PPP2CA;ortholog
HUMAN|HG	PRKAR2A	cAMP-dependent protein kinase type II-alpha regulatory subunit;PRKAR2A;ortholog
HUMAN|HG	PRMT1	Protein arginine N-methyltransferase 1;PRMT1;ortholog
HUMAN|HG	PRMT5	Protein arginine N-methyltransferase 5;PRMT5;ortholog
HUMAN|HG	PRPF19	Pre-mRNA-processing factor 19;PRPF19;ortholog
HUMAN|HG	PRPF3	U4/U6 small nuclear ribonucleoprotein Prp3;PRPF3;ortholog
HUMAN|HG	PRPF31	U4/U6 small nuclear ribonucleoprotein Prp31;PRPF31;ortholog
HUMAN|HG	PRPF4	U4/U6 small nuclear ribonucleoprotein Prp4;PRPF4;ortholog
HUMAN|HG	PRPF6	Pre-mRNA-processing factor 6;PRPF6;ortholog
HUMAN|HG	PRPS1	Ribose-phosphate pyrophosphokinase 1;PRPS1;ortholog
HUMAN|HG	PURA	Transcriptional activator protein Pur-alpha;PURA;ortholog
HUMAN|HG	PURB	Transcriptional activator protein Pur-beta;PURB;ortholog
HUMAN|HG	RAN	GTP-binding nuclear protein Ran;RAN;ortholog
HUMAN|HG	RAVER1	Ribonucleoprotein PTB-binding 1;RAVER1;ortholog
HUMAN|HG	RBFOX2	RNA binding protein fox-1 homolog 2;RBFOX2;ortholog
HUMAN|HG	RBM25	RNA-binding protein 25;RBM25;ortholog
HUMAN|HG	RBM5	RNA-binding protein 5;RBM5;ortholog
HUMAN|HG	RING1	E3 ubiquitin-protein ligase RING1;RING1;ortholog
HUMAN|HG	RNASEH2A	Ribonuclease H2 subunit A;RNASEH2A;ortholog
HUMAN|HG	RNASEH2C	Ribonuclease H2 subunit C;RNASEH2C;ortholog
HUMAN|HG	RNF20	E3 ubiquitin-protein ligase BRE1A;RNF20;ortholog
HUMAN|HG	RNF40	E3 ubiquitin-protein ligase BRE1B;RNF40;ortholog
HUMAN|HG	RNMT	mRNA cap guanine-N7 methyltransferase;RNMT;ortholog
HUMAN|HG	RPL18A	60S ribosomal protein L18a;RPL18A;ortholog
HUMAN|HG	RQCD1	CCR4-NOT transcription complex subunit 9;CNOT9;ortholog
HUMAN|HG	RSL1D1	Ribosomal L1 domain-containing protein 1;RSL1D1;ortholog
HUMAN|HG	RTCB	tRNA-splicing ligase RtcB homolog;RTCB;ortholog
HUMAN|HG	SCRIB	Protein scribble homolog;SCRIB;ortholog
HUMAN|HG	SEPT10	Septin-10;SEPT10;ortholog
HUMAN|HG	SEPT2	Septin-2;SEPT2;ortholog
HUMAN|HG	SEPT6	Septin-6;SEPT6;ortholog
HUMAN|HG	SEPT7	Septin-7;SEPT7;ortholog
HUMAN|HG	SEPT8	Septin-8;SEPT8;ortholog
HUMAN|HG	SEPT9	Septin-9;SEPT9;ortholog
HUMAN|HG	SF1	Splicing factor 1;SF1;ortholog
HUMAN|HG	SF3A1	Splicing factor 3A subunit 1;SF3A1;ortholog
HUMAN|HG	SF3A3	Splicing factor 3A subunit 3;SF3A3;ortholog
HUMAN|HG	SF3B1	Splicing factor 3B subunit 1;SF3B1;ortholog
HUMAN|HG	SF3B2	Splicing factor 3B subunit 2;SF3B2;ortholog
HUMAN|HG	SF3B3	Splicing factor 3B subunit 3;SF3B3;ortholog
HUMAN|HG	SF3B6	Splicing factor 3B subunit 6;SF3B6;ortholog
HUMAN|HG	SNRNP40	U5 small nuclear ribonucleoprotein 40 kDa protein;SNRNP40;ortholog
HUMAN|HG	SNRNP70	U1 small nuclear ribonucleoprotein 70 kDa;SNRNP70;ortholog
HUMAN|HG	SNRPA	U1 small nuclear ribonucleoprotein A;SNRPA;ortholog
HUMAN|HG	SNRPA1	U2 small nuclear ribonucleoprotein A';SNRPA1;ortholog
HUMAN|HG	SNRPC	U1 small nuclear ribonucleoprotein C;SNRPC;ortholog
HUMAN|HG	SNRPD2	Small nuclear ribonucleoprotein Sm D2;SNRPD2;ortholog
HUMAN|HG	SNRPD3	Small nuclear ribonucleoprotein Sm D3;SNRPD3;ortholog
HUMAN|HG	SNRPE	Small nuclear ribonucleoprotein E;SNRPE;ortholog
HUMAN|HG	SNRPN	Small nuclear ribonucleoprotein-associated protein N;SNRPN;ortholog
HUMAN|HG	SRRT	Serrate RNA effector molecule homolog;SRRT;ortholog
HUMAN|HG	SRSF1	Serine/arginine-rich splicing factor 1;SRSF1;ortholog
HUMAN|HG	SRSF10	Serine/arginine-rich splicing factor 10;SRSF10;ortholog
HUMAN|HG	SRSF2	Serine/arginine-rich splicing factor 2;SRSF2;ortholog
HUMAN|HG	SRSF7	Serine/arginine-rich splicing factor 7;SRSF7;ortholog
HUMAN|HG	SRSF9	Serine/arginine-rich splicing factor 9;SRSF9;ortholog
HUMAN|HG	TRA2B	Transformer-2 protein homolog beta;TRA2B;ortholog
HUMAN|HG	TRAF7	E3 ubiquitin-protein ligase TRAF7;TRAF7;ortholog
HUMAN|HG	TRIOBP	TRIO and F-actin-binding protein;TRIOBP;ortholog
HUMAN|HG	TRIP4	Activating signal cointegrator 1;TRIP4;ortholog
HUMAN|HG	U2SURP	U2 snRNP-associated SURP motif-containing protein;U2SURP;ortholog
HUMAN|HG	USP10	Ubiquitin carboxyl-terminal hydrolase 32;USP32;ortholog
HUMAN|HG	USP39	U4/U6.U5 tri-snRNP-associated protein 2;USP39;ortholog
HUMAN|HG	USP7	Ubiquitin carboxyl-terminal hydrolase 7;USP7;ortholog
HUMAN|HG	VIM	Vimentin;VIM;ortholog
HUMAN|HG	WBP11	WW domain-binding protein 11;WBP11;ortholog
HUMAN|HG	WBSCR22	Probable 18S rRNA (guanine-N(7))-methyltransferase;BUD23;ortholog
HUMAN|HG	WDR61	WD repeat-containing protein 61;WDR61;ortholog
HUMAN|HG	WDR77	Methylosome protein 50;WDR77;ortholog
HUMAN|HG	WTAP	Pre-mRNA-splicing regulator WTAP;WTAP;ortholog
HUMAN|HG	YLPM1	YLP motif-containing protein 1;YLPM1;ortholog
HUMAN|HG	YTHDF2	YTH domain-containing family protein 2;YTHDF2;ortholog

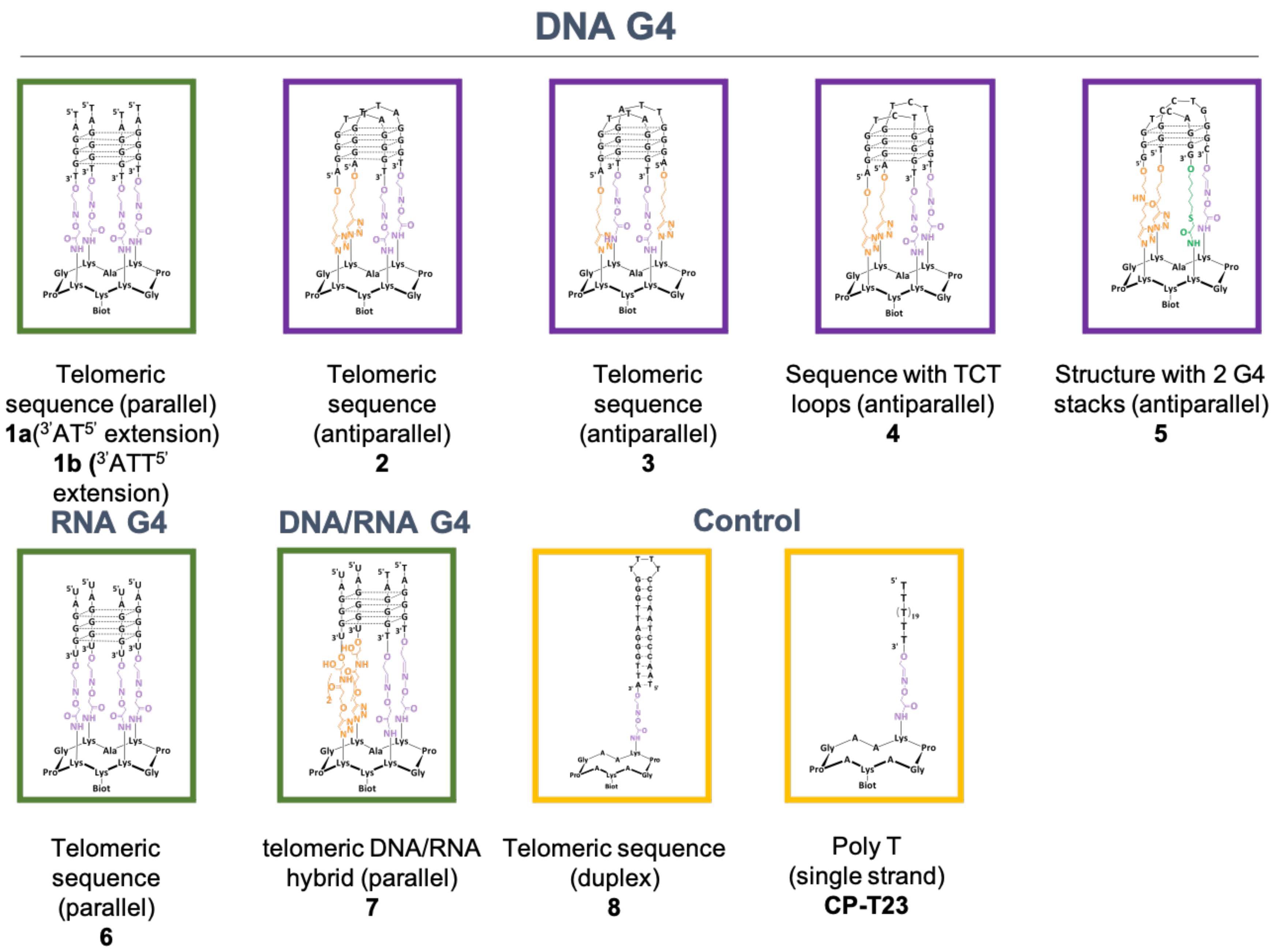

Supplementary Figure S1

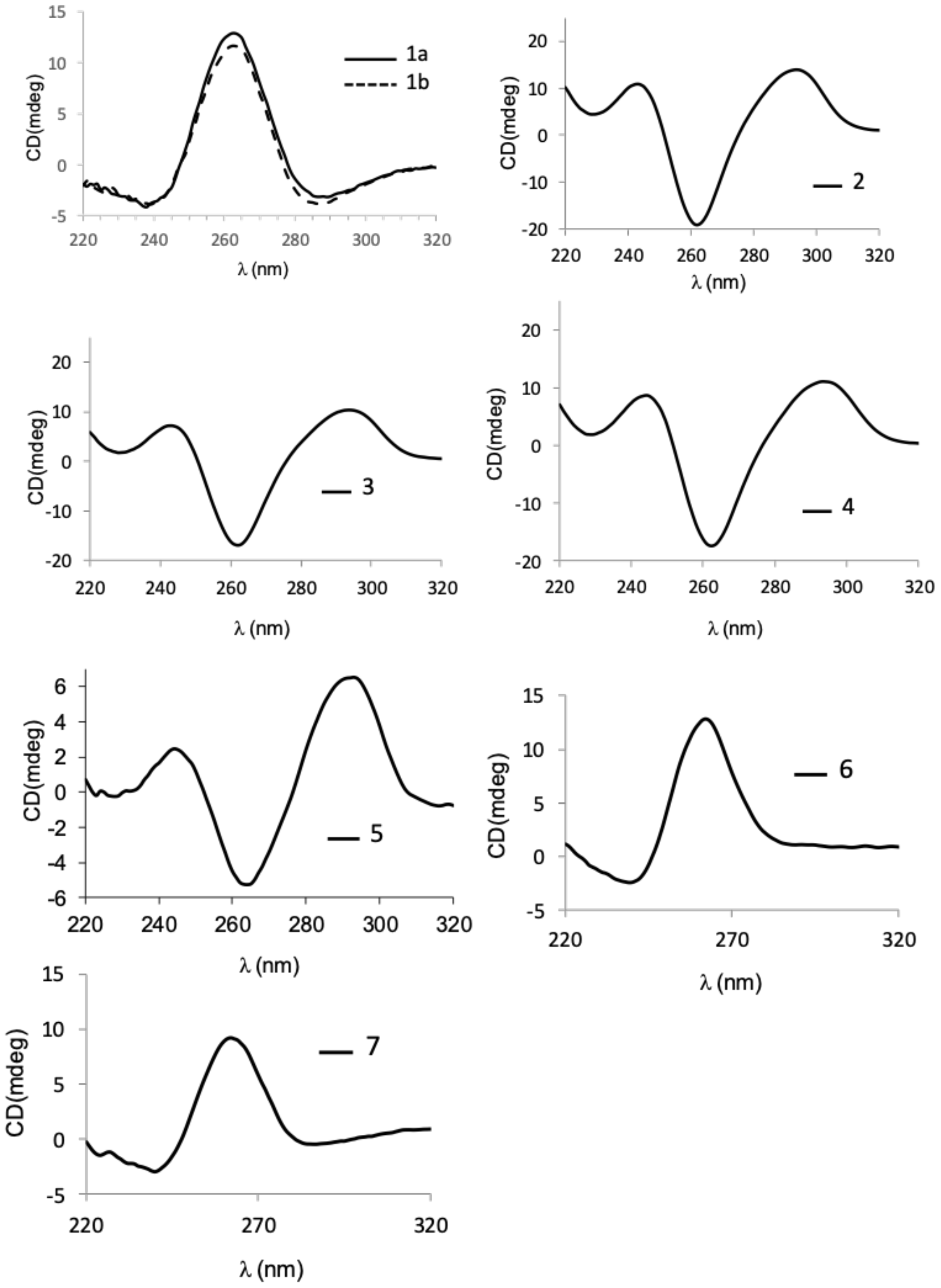

Supplementary Figure S2

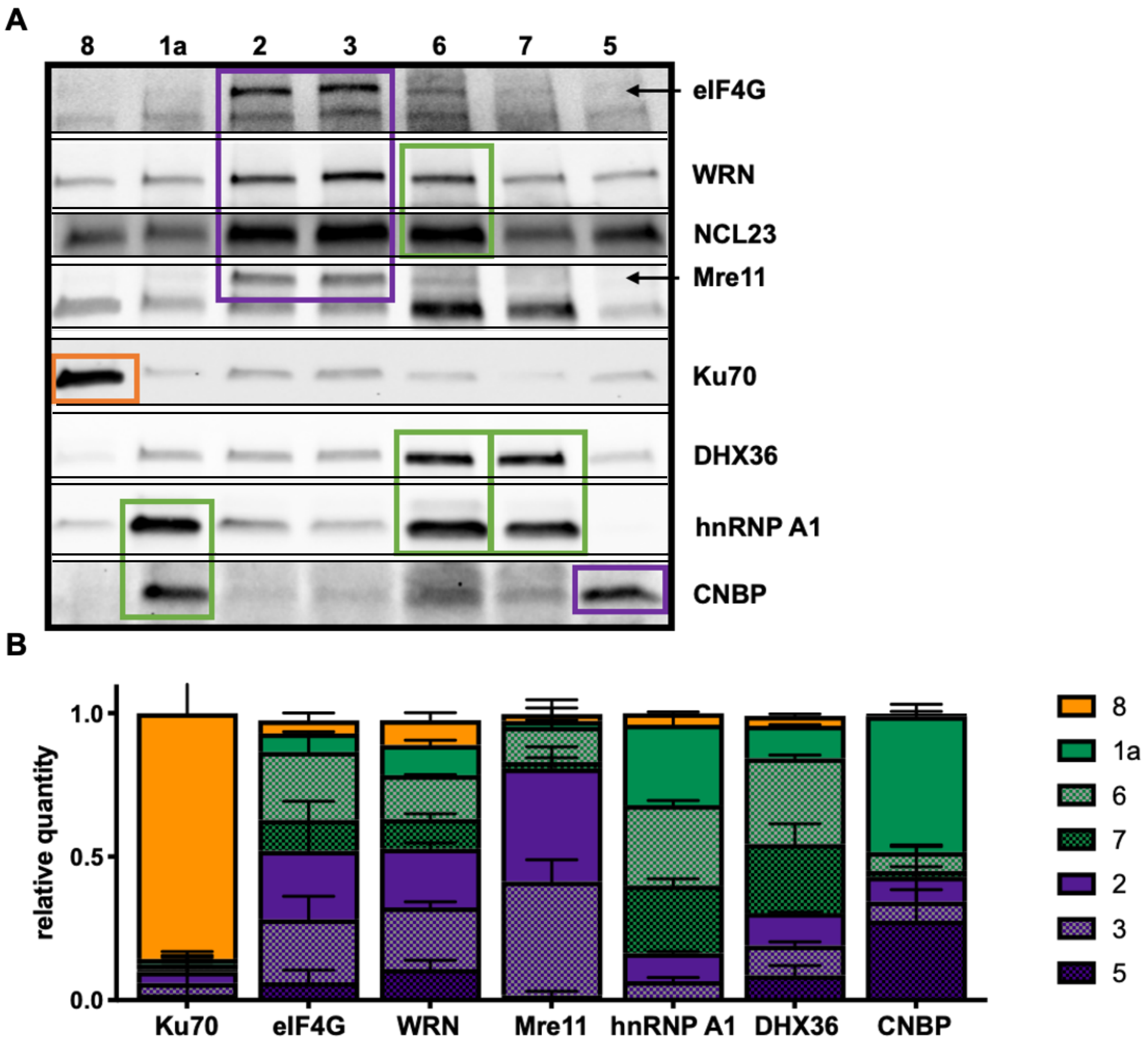

Supplementary Figure S3

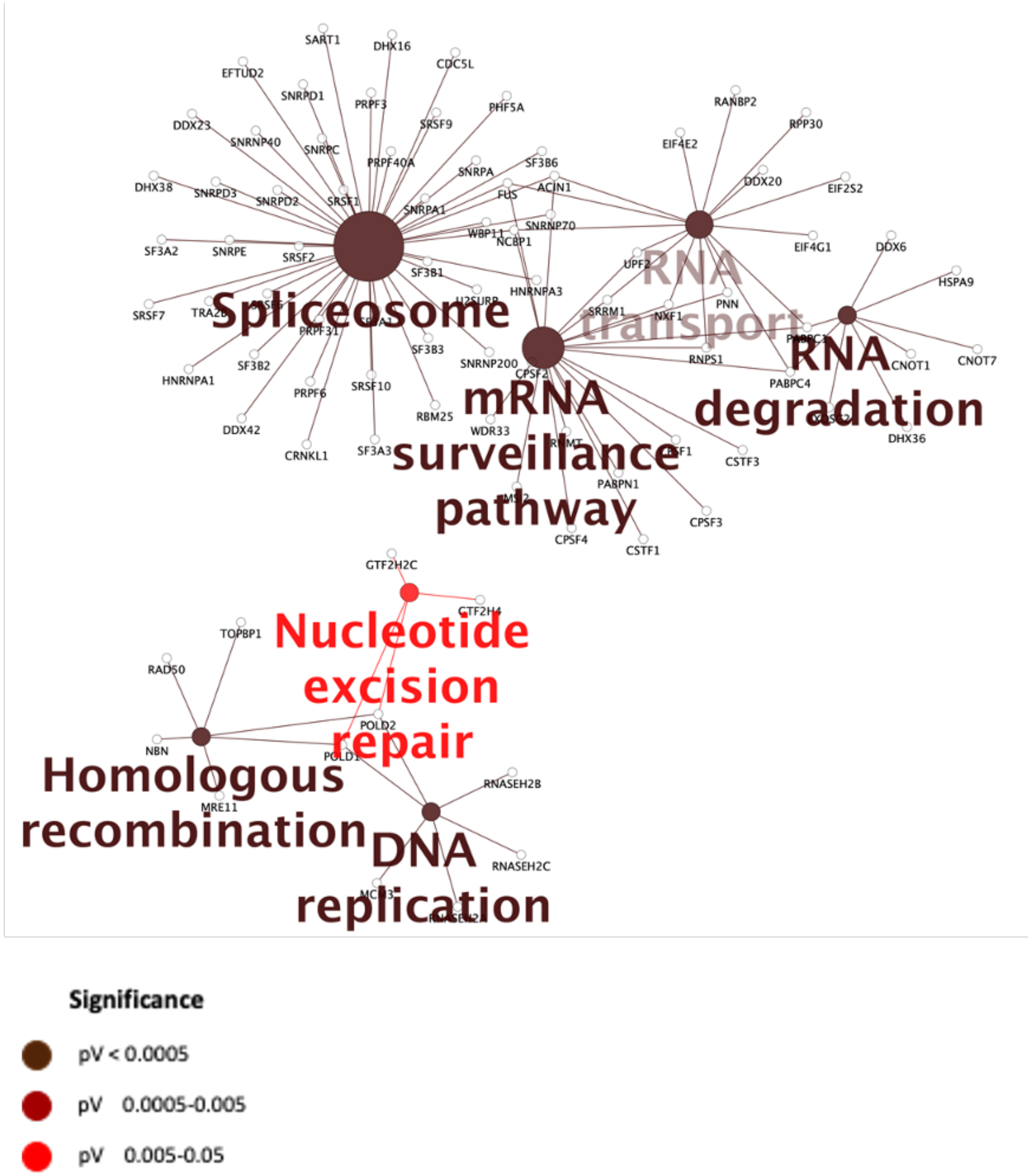

Supplementary Figure S4

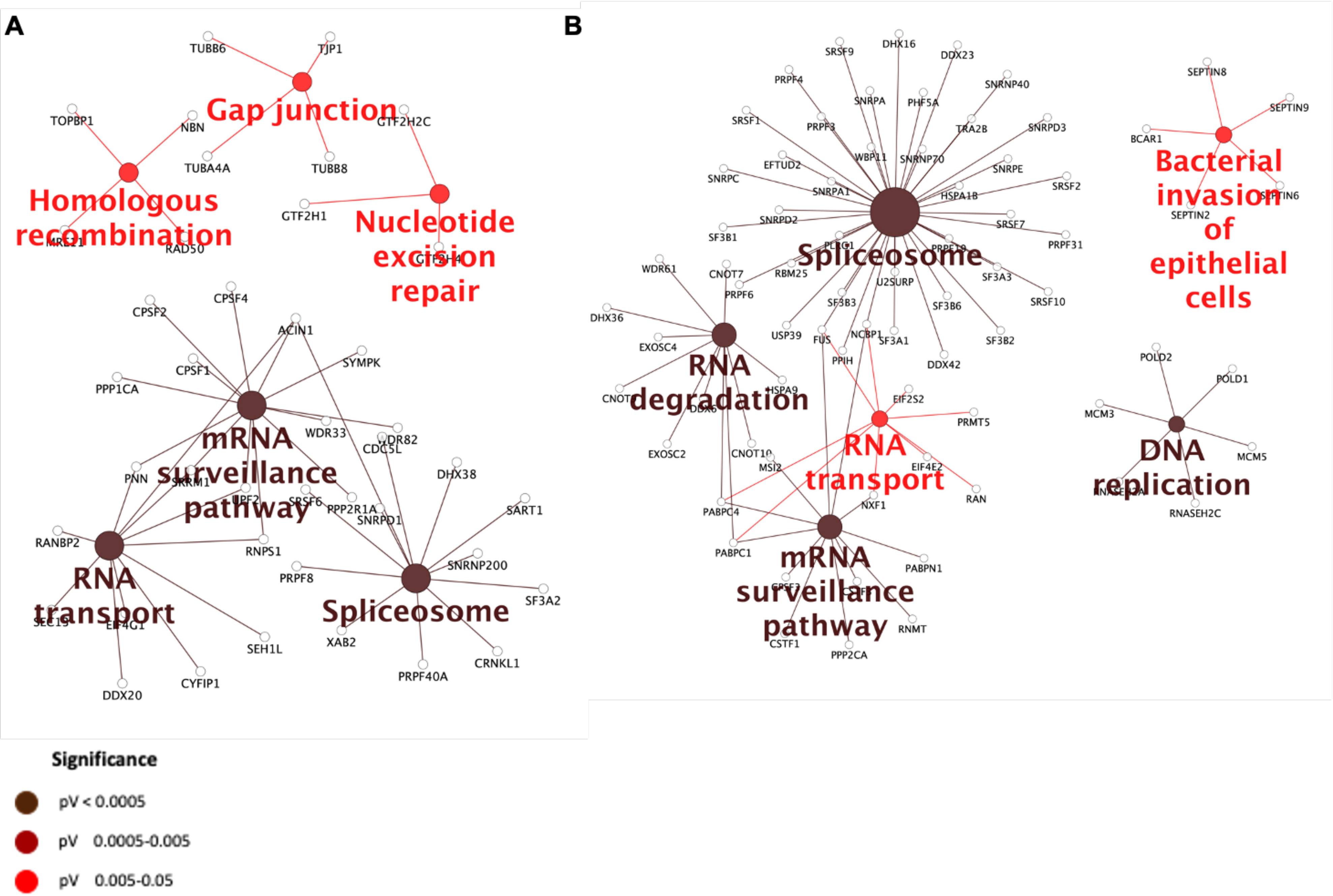

Supplementary Figure S5

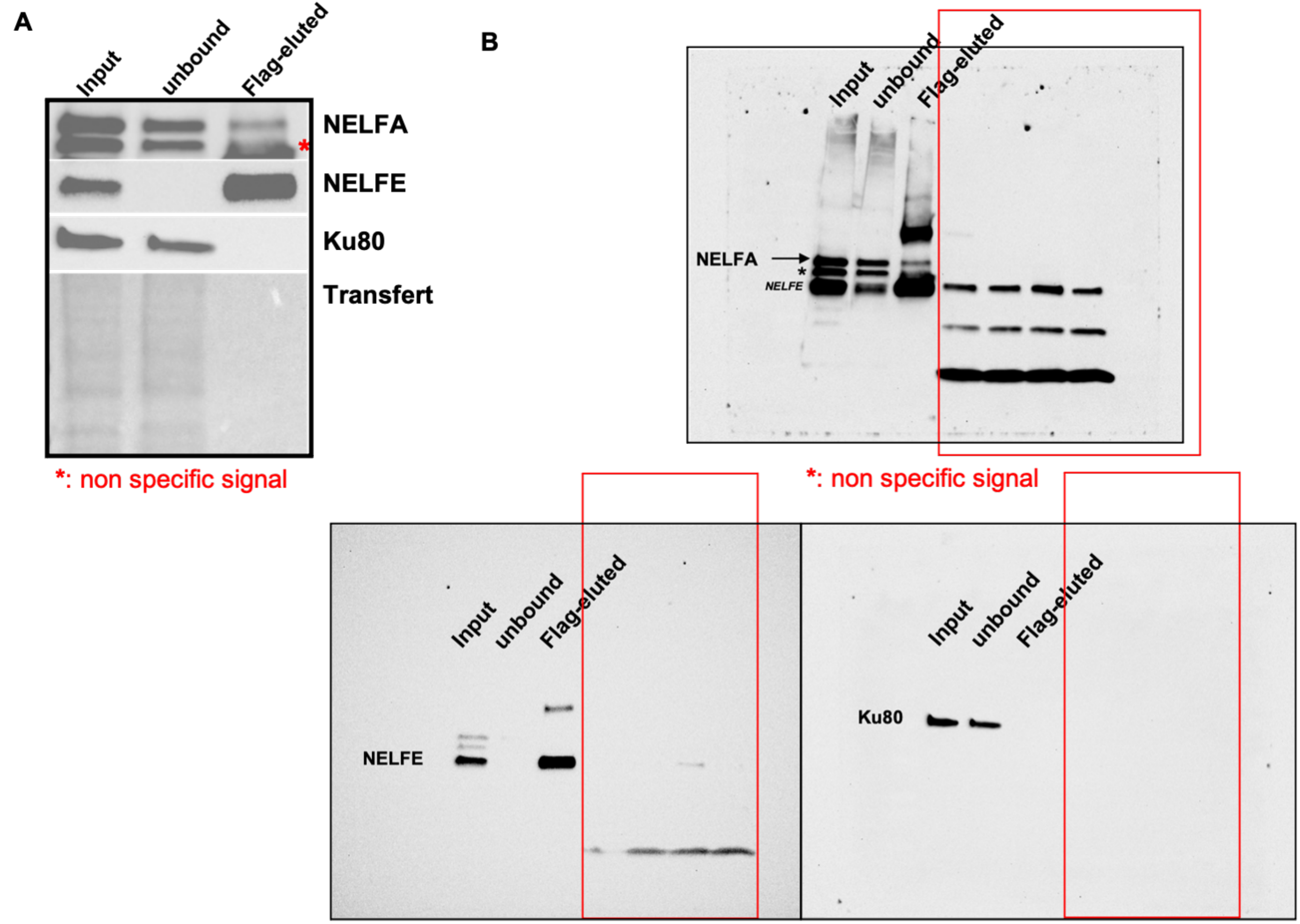

Supplementary Figure S6

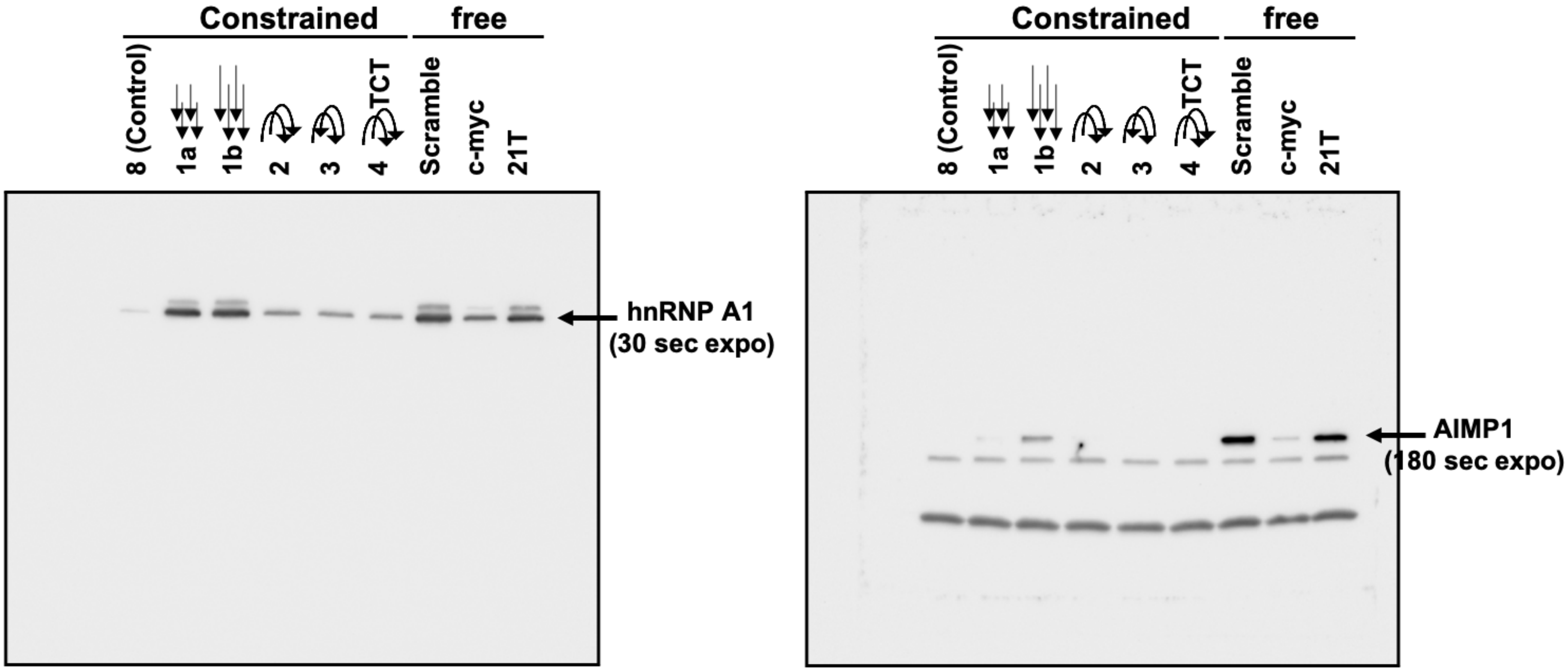

Uncropped and unprocessed versions of western-blot signals from Figure 5.
Chemiluminescence signals were acquired using the ChemyDoc Touching Imaging System (Bio-Rad).
Exposition times are indicated.
When additional signals corresponding to previous hybridizations with other antibodies are present they are indicated in Italics

Supplementary Figure S7A

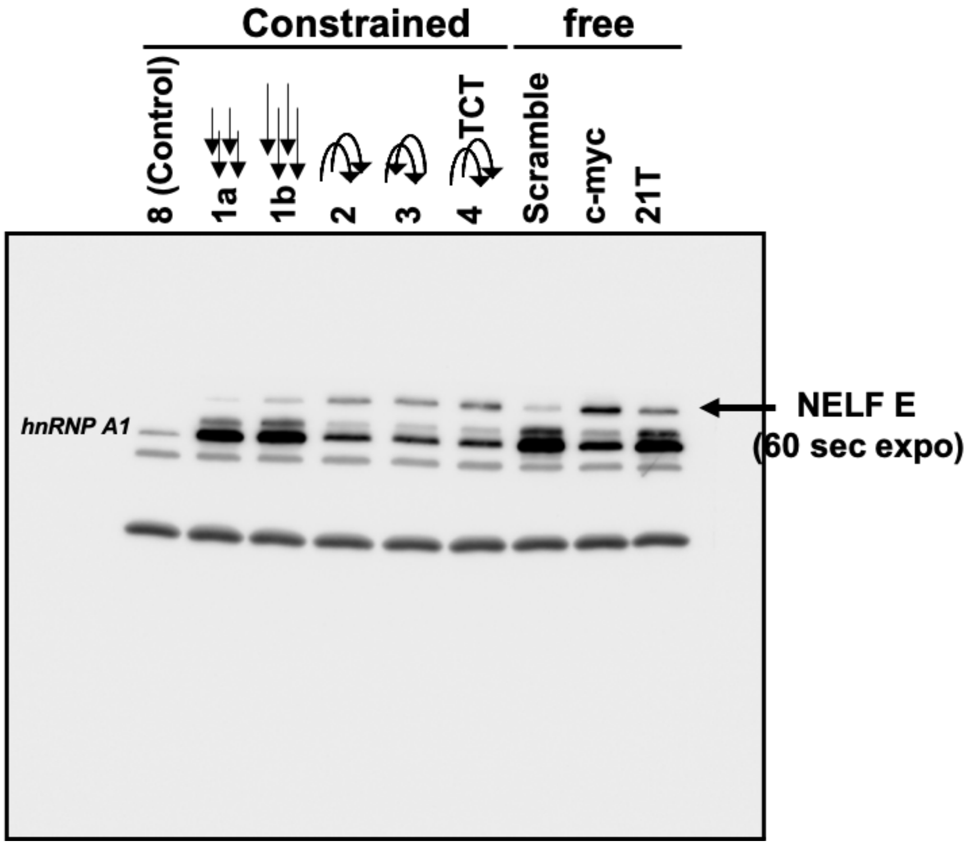

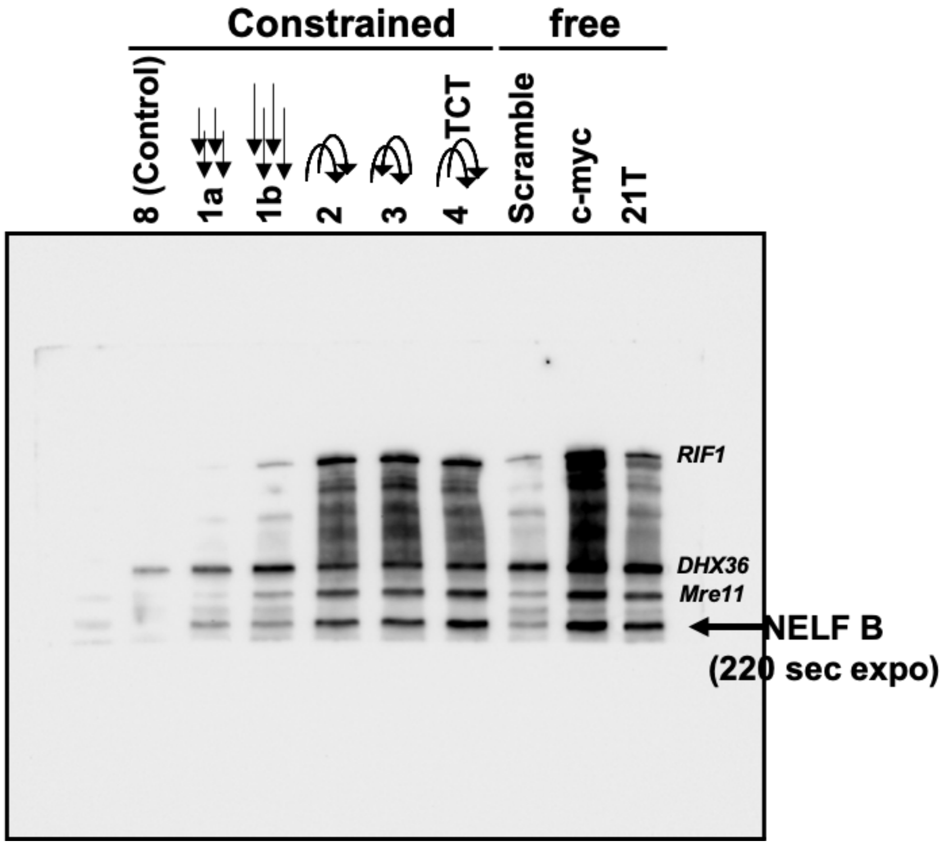

Uncropped and unprocessed versions of western-blot signals from Figure 5.
Chemiluminescence signals were acquired using the ChemyDoc Touching Imaging System (Bio-Rad).
Exposition times are indicated.
When additional signals corresponding to previous hybridizations with other antibodies are present they are indicated in Italics

Supplementary Figure S7B

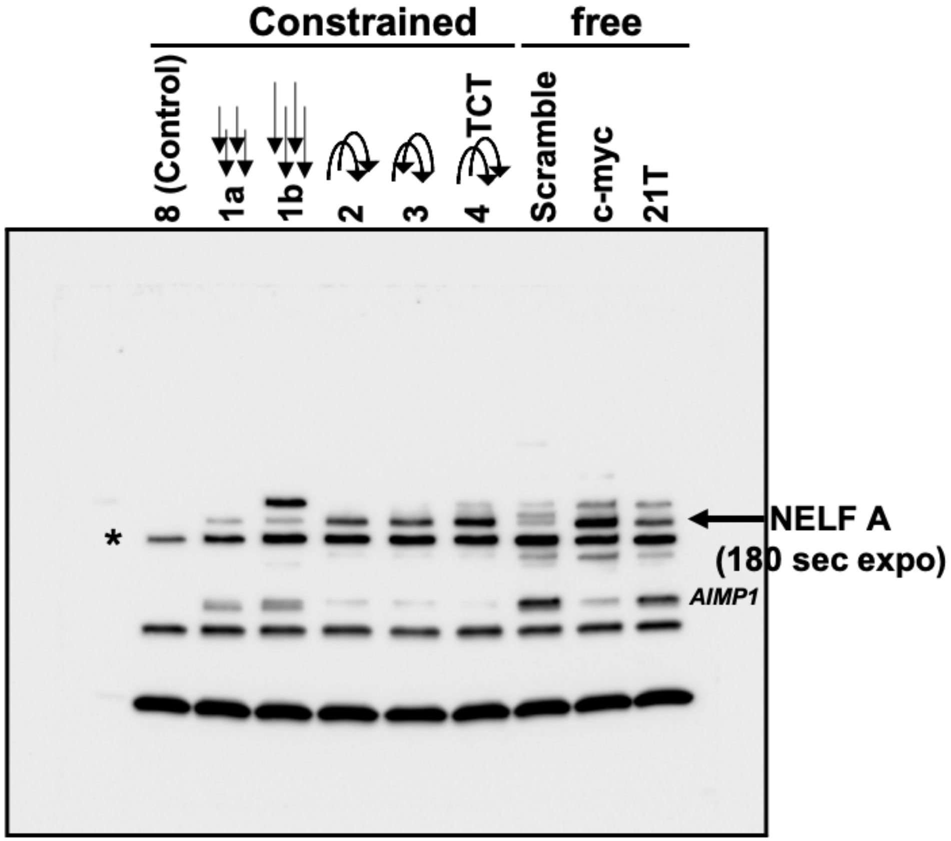

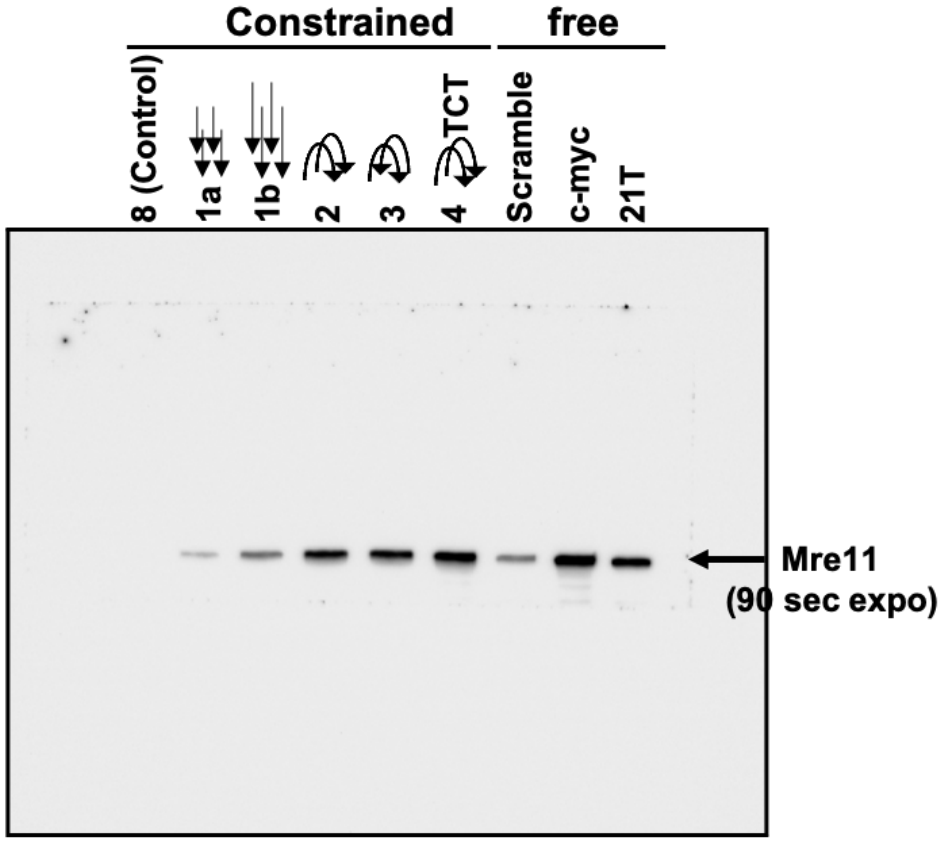

*: non-specific signal

Uncropped and unprocessed versions of western-blot signals from Figure 5.
Chemiluminescence signals were acquired using the ChemyDoc Touching Imaging System (Bio-Rad).
Exposition times are indicated.
When additional signals corresponding to previous hybridizations with other antibodies are present they are indicated in Italics

Supplementary Figure S7C

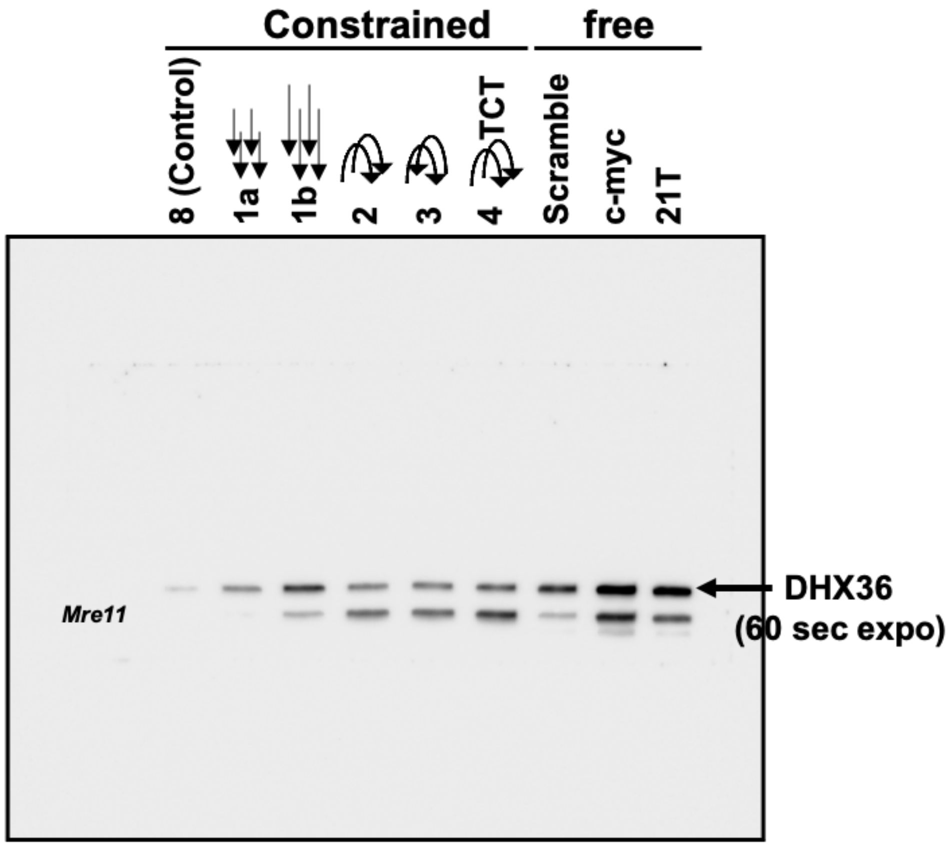

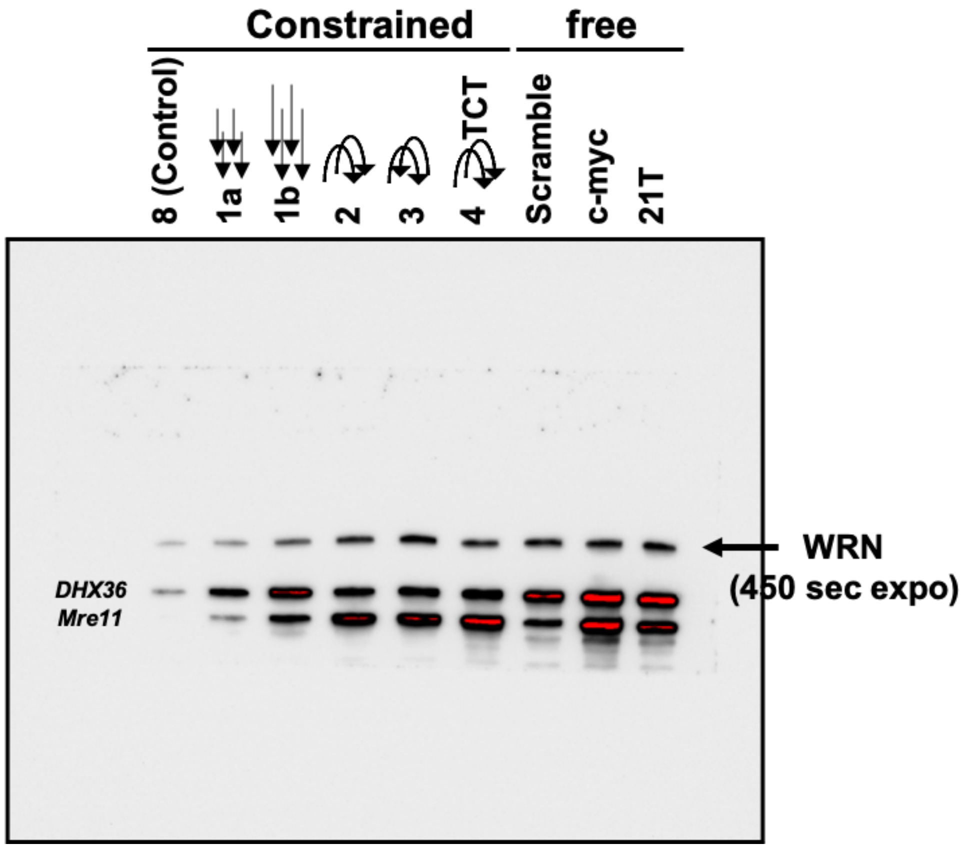

Uncropped and unprocessed versions of western-blot signals from Figure 5.
Chemiluminescence signals were acquired using the ChemyDoc Touching Imaging System (Bio-Rad).
Exposition times are indicated.
When additional signals corresponding to previous hybridizations with other antibodies are present they are indicated in Italics

Supplementary Figure S7D

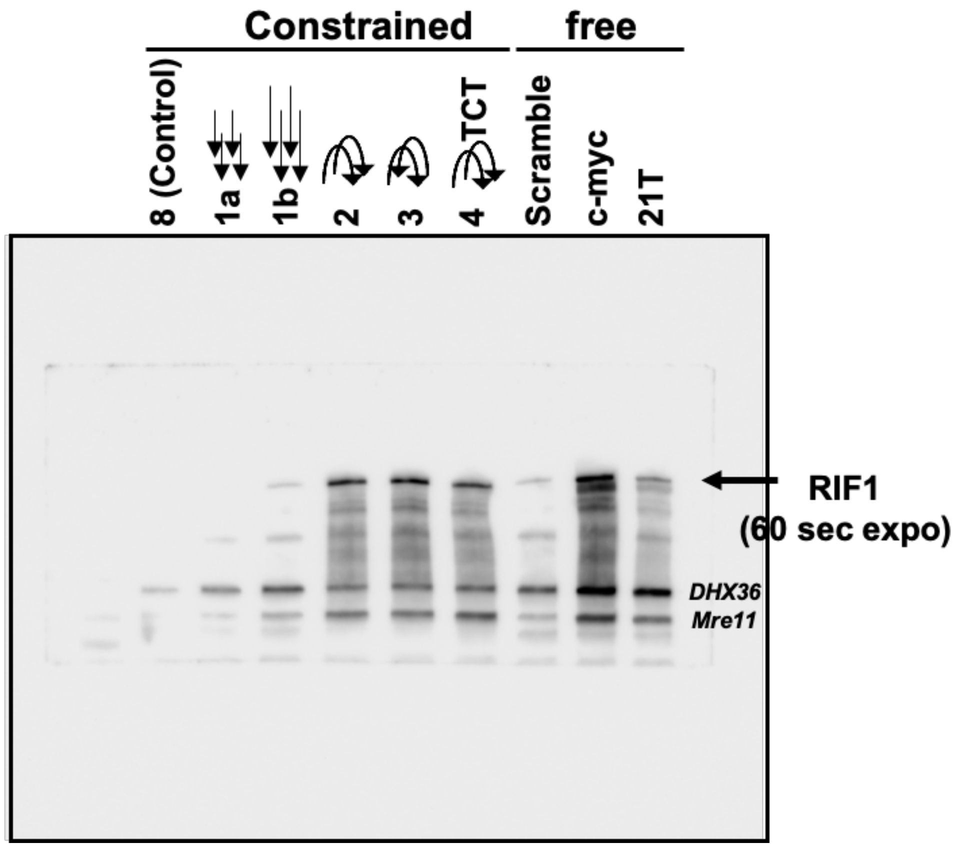

Uncropped and unprocessed versions of western-blot signals from Figure 5.
Chemiluminescence signals were acquired using the ChemyDoc Touching Imaging System (Bio-Rad).
Exposition times are indicated.
When additional signals corresponding to previous hybridizations with other antibodies are present they are indicated Italics

Supplementary Figure S7E

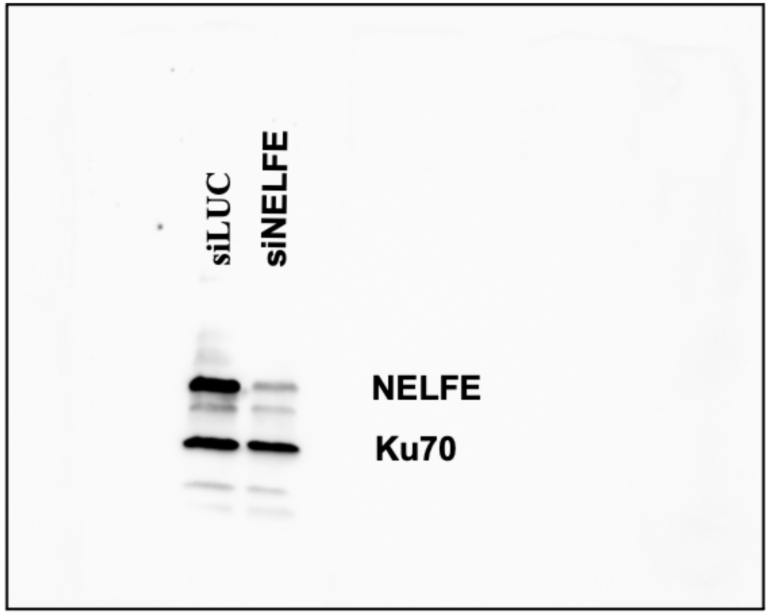

Uncropped and unprocessed versions of western-blot signals from Figure 6C. Chemiluminescence signals were acquired using the ChemyDoc Touching Imaging System (Bio-Rad).

Supplementary Figure S8

Modification of images:

#1: for quantification → DAPI (p. 2): selection of nuclei
→ γH2AX (p. 3): removal of background noise without brightness and contrast modification

#2: for figure → Overlay of nuclei edges (from #1 modification of DAPI) over γH2AX staining (from #1 modification of γH2AX with modified brightness and contrast) (p. 3 only)

✓ Same background noise removal for all images, for all conditions of treatment and for all experiments (n=3)
✓ Same brightness and contrast applied for all images representative of each conditions

	siLUC NT	siLUC PDS	siNELFE NT	siNELFE PDS
# Images	7	19	34	48
Raw images	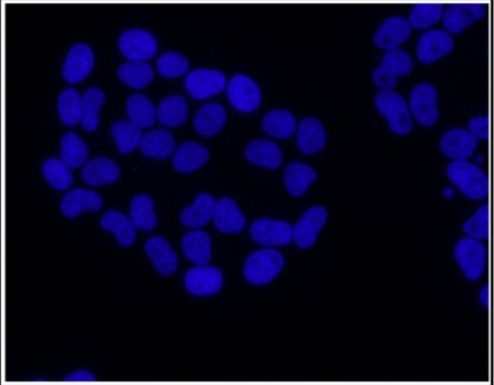	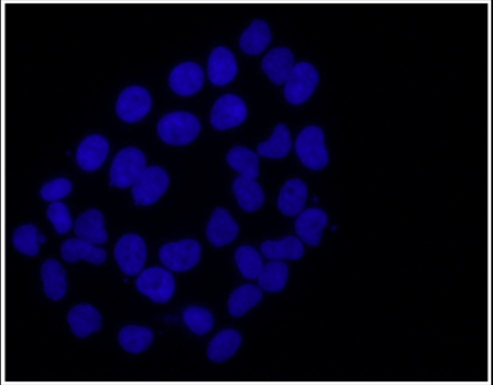	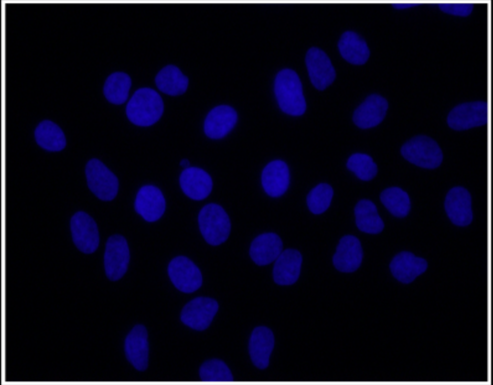	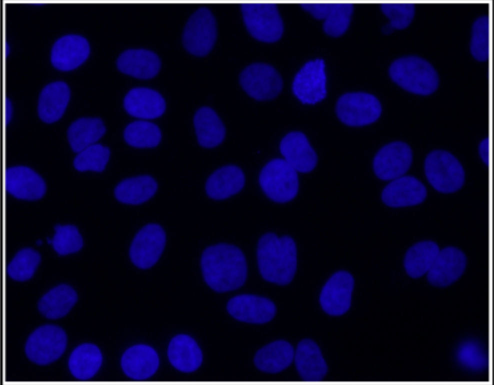
Modified imgs for quantif.	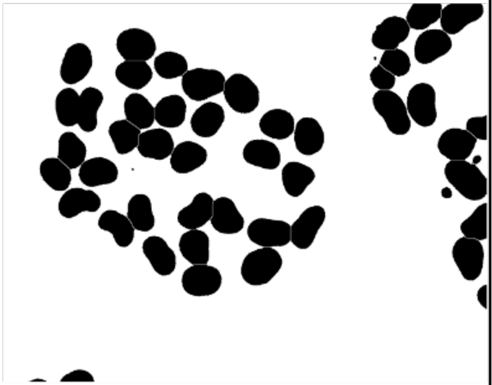	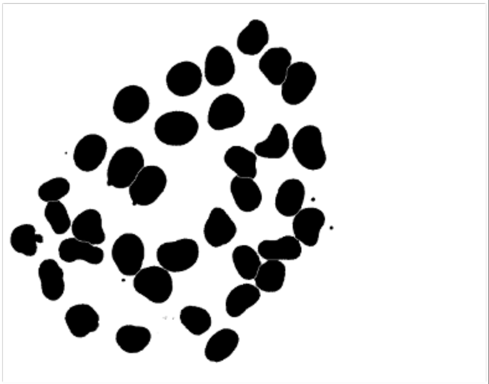	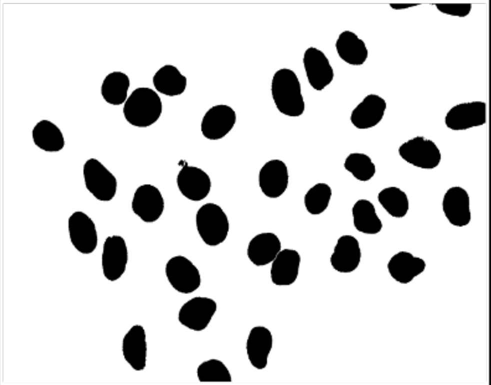	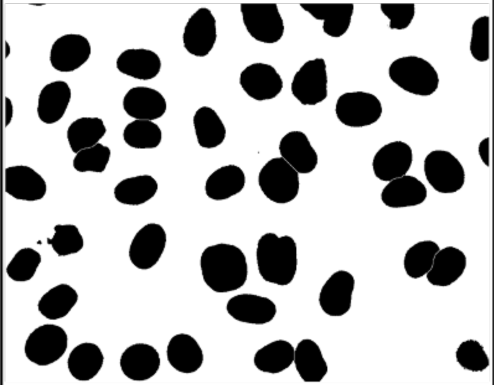

Supplementary Figure S9A

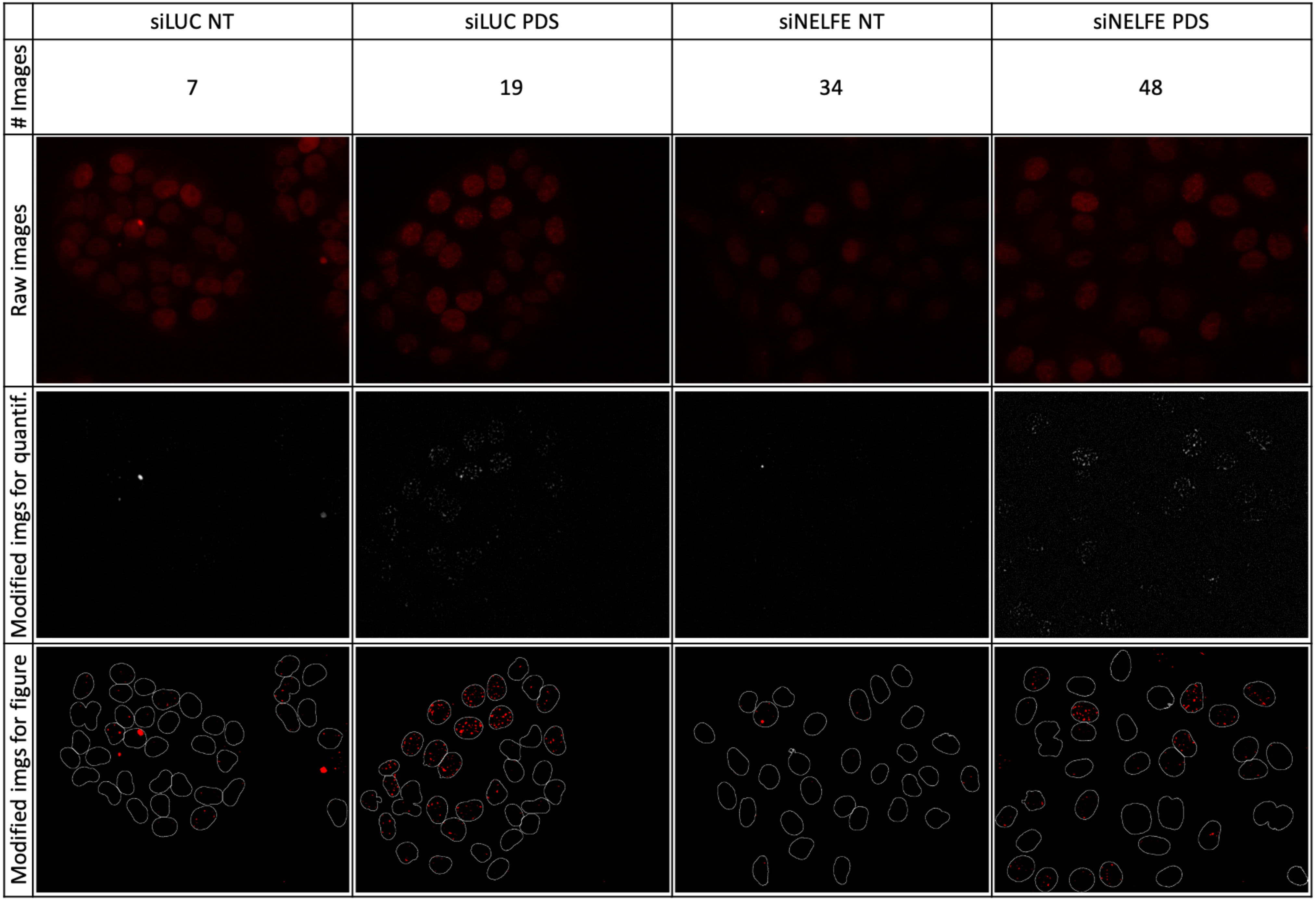

Supplementary Figure S9B

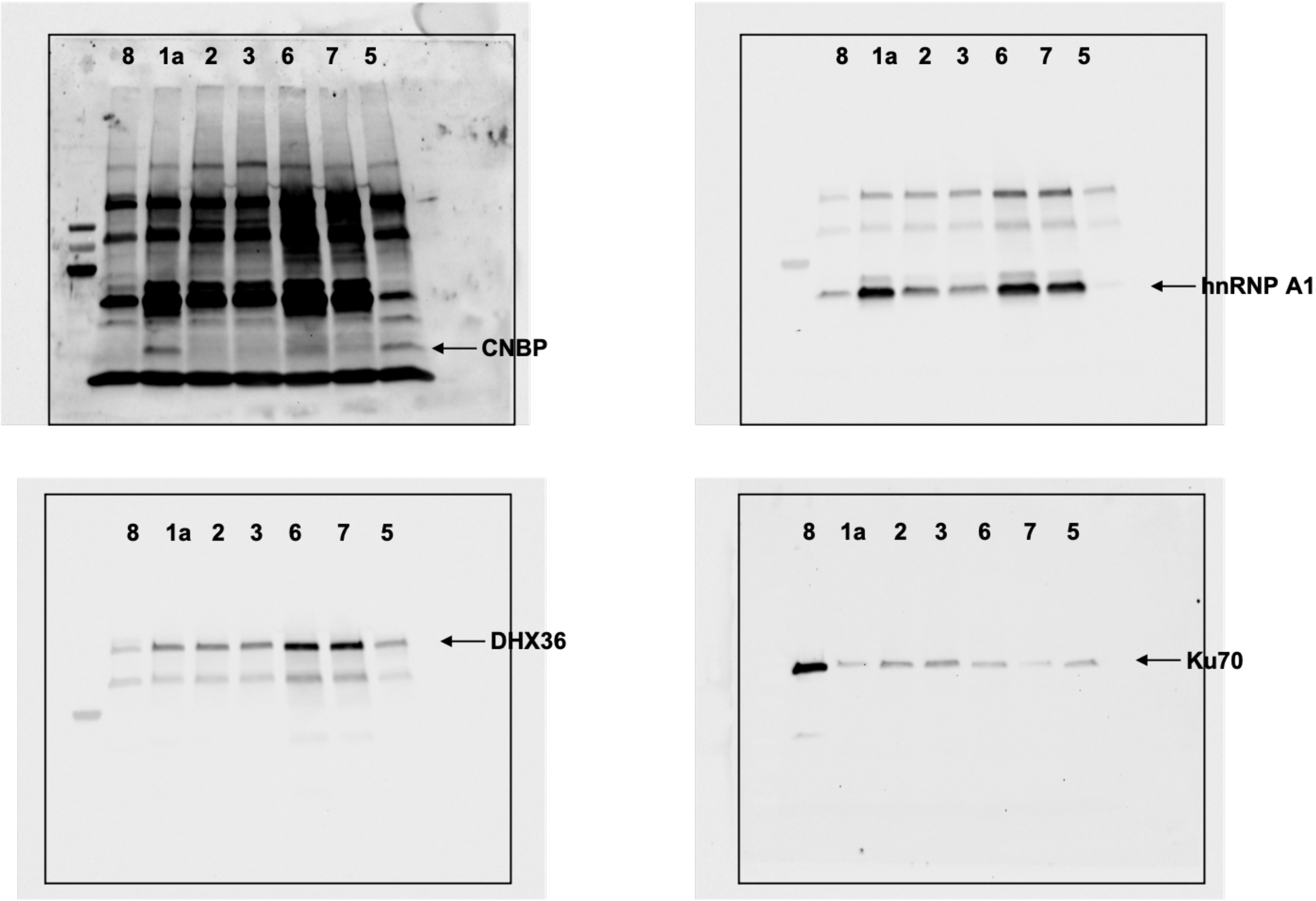

Supplementary Figure S10A

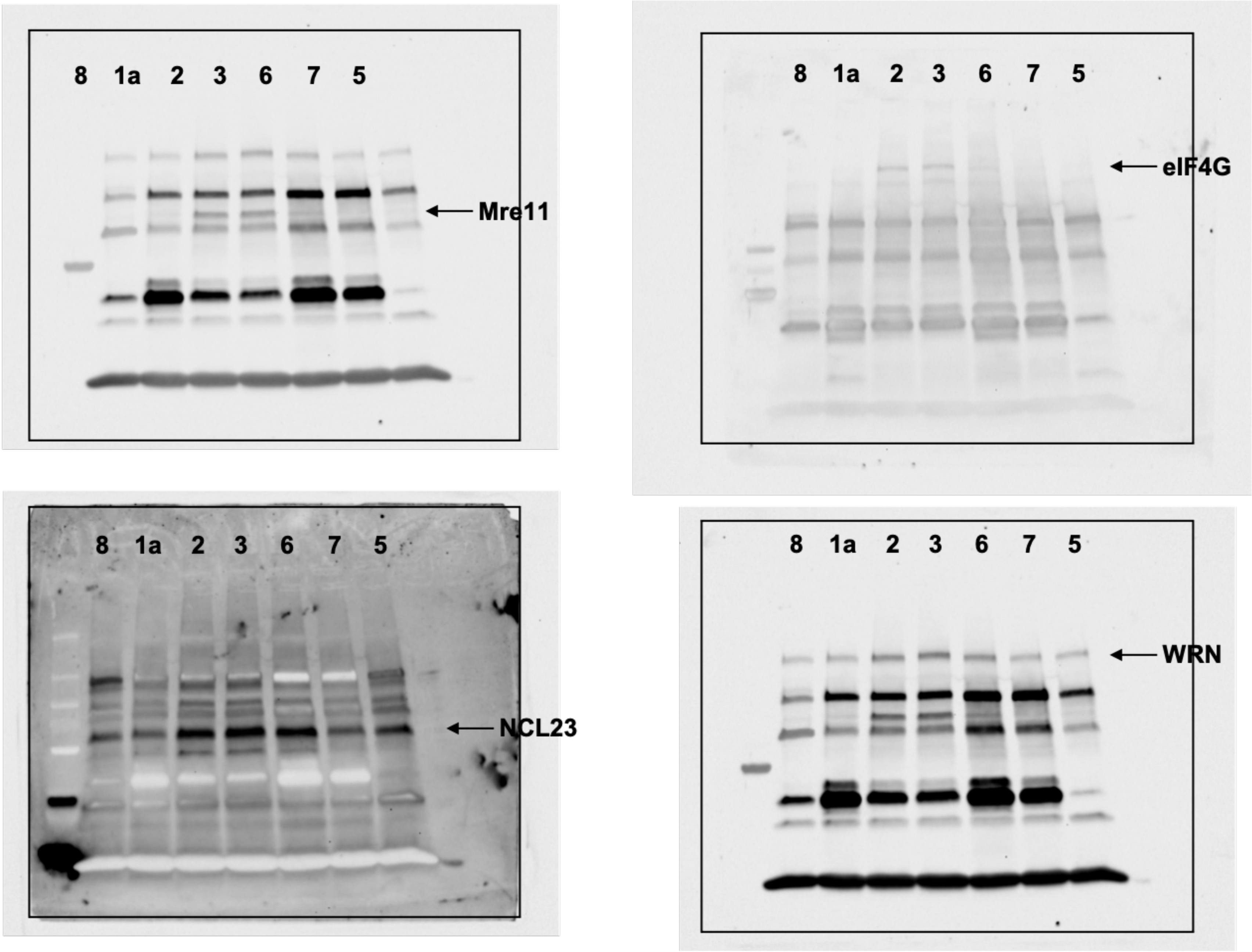

Supplementary Figure S10B
